# Supplementary material for: Feasibility and acceptability of a dietary intervention study to reduce salt intake and increase high-nitrate vegetable consumption among middle-aged and older Malaysian adults with elevated blood pressure: a study protocol
Source: BMJ Open. 2020 Aug 27;10(8):e035453. doi: 10.1136/bmjopen-2019-035453 (PMC7454174; doi:10.1136/bmjopen-2019-035453)
Supplement: Author's manuscript [file bmjopen-2019-035453.draft_revisions.pdf]

BMJ Open is committed to open peer review. As part of this commitment we make the peer review history of every article we publish publicly available.

When an article is published we post the peer reviewers' comments and the authors' responses online. We also post the versions of the paper that were used during peer review. These are the versions that the peer review comments apply to.

The versions of the paper that follow are the versions that were submitted during the peer review process. They are not the versions of record or the final published versions. They should not be cited or distributed as the published version of this manuscript.

BMJ Open is an open access journal and the full, final, typeset and author-corrected version of record of the manuscript is available on our site with no access controls, subscription charges or pay-per-view fees (http://bmjopen.bmj.com).

If you have any questions on BMJ Open's open peer review process please email info.bmjopen@bmj.com

BMJ Open

The feasibility of a mixed-methods, dietary intervention study to reduce salt intake and increase high-nitrate vegetable consumption among middle-aged and older Malaysian adults with elevated blood pressure: a study protocol

Journal:	*BMJ Open*
Manuscript ID	bmjopen-2019-035453
Article Type:	Protocol
Date Submitted by the Author:	01-Nov-2019
Complete List of Authors:	McGrattan, Andrea; Newcastle University, Institute of Health and Society<br>Mohan, Devi; Monash University - Malaysia Campus, Global Public Health, School of Medicine<br>Chua, Pei Wei; Monash University - Malaysia Campus, Global Public Health. Jeffrey Cheah School of Medicine and Health Sciences; South East Asia Community Observatory (SEACO)<br>Mat Hussin, Azizah; Universiti Kuala Lumpur, Kampus Cawangan Institute of Medical Science Technology<br>Soh, Yee Chang; Monash University - Malaysia Campus, Global Public Health; Jeffrey Cheah School of Medicine; South East Asia Community Observatory (SEACO)<br>Alawad, Mawada; Monash University - Malaysia Campus, Global Public Health. Jeffrey Cheah School of Medicine and Health Sciences; South East Asia Community Observatory (SEACO)<br>bin Kassim, Zaid; District Health Office (PKD) Segamat<br>bin Mohd Ghazali, Ahmad Nizal; District Health Office (PKD) Segamat<br>Stephan, Blossom; University of Nottingham, School of Medicine<br>Allotey, Pascale; Monash University - Malaysia Campus, Global Public Health. Jeffrey Cheah School of Medicine and Health Sciences; United Nations University International Institute for Global Health<br>Reidpath, Daniel; Monash University - Malaysia Campus, Global Public Health. Jeffrey Cheah School of Medicine and Health Sciences; South East Asia Community Observatory (SEACO)<br>Robinson, Louise; Newcastle University, Institute of Health and Society<br>Siervo, Mario; University of Nottingham, School of Life Sciences
Keywords:	Dementia < NEUROLOGY, NUTRITION & DIETETICS, PUBLIC HEALTH

SCHOLARONE™
Manuscripts

I, the Submitting Author has the right to grant and does grant on behalf of all authors of the Work (as defined in the below author licence), an exclusive licence and/or a non-exclusive licence for contributions from authors who are: i) UK Crown employees; ii) where BMJ has agreed a CC-BY licence shall apply, and/or iii) in accordance with the terms applicable for US Federal Government officers or employees acting as part of their official duties; on a worldwide, perpetual, irrevocable, royalty-free basis to BMJ Publishing Group Ltd ("BMJ") its licensees and where the relevant Journal is co-owned by BMJ to the co-owners of the Journal, to publish the Work in this journal and any other BMJ products and to exploit all rights, as set out in our licence.

The Submitting Author accepts and understands that any supply made under these terms is made by BMJ to the Submitting Author unless you are acting as an employee on behalf of your employer or a postgraduate student of an affiliated institution which is paying any applicable article publishing charge ("APC") for Open Access articles. Where the Submitting Author wishes to make the Work available on an Open Access basis (and intends to pay the relevant APC), the terms of reuse of such Open Access shall be governed by a Creative Commons licence – details of these licences and which Creative Commons licence will apply to this Work are set out in our licence referred to above.

Other than as permitted in any relevant BMJ Author's Self Archiving Policies, I confirm this Work has not been accepted for publication elsewhere, is not being considered for publication elsewhere and does not duplicate material already published. I confirm all authors consent to publication of this Work and authorise the granting of this licence.

For peer review only

TITLE

The feasibility of a mixed-methods, dietary intervention study to reduce salt intake and increase high-nitrate vegetable consumption among middle-aged and older Malaysian adults with elevated blood pressure: a study protocol

AUTHORS

Andrea M McGrattan[1*], Devi Mohan[2], Pei Wei Chua[2,7], Azizah Mat Hussin[3], Yee Chang Soh[2,7], Mawada Azhari Sidahmed Alawad[2,7], Zaid Bin Kassim[4], Ahmad Nizal Bin Mohd Ghazali[4], Blossom C M Stephan[5], Pascale Allotey[2,6], Daniel D Reidpath[2,7], Louise Robinson[1], Mario Siervo[8]

AFFILIATIONS

[1]Institute of Health and Society, Newcastle University, Newcastle, United Kingdom.

[2]Global Public Health. Jeffrey Cheah School of Medicine and Health Sciences, Monash University Malaysia, Selangor, Malaysia.

[3]Universiti Kuala Lumpur, Kampus Cawangan Institute of Medical Science Technology, Kuala Lumpur, Malaysia.

[4]District Health Office (PKD) Segamat, Jalan Gudang Ubat 85000, Segamat Johor, Malaysia

[5] School of Medicine, The University of Nottingham, United Kingdom.

[6] Director of the United Nations University International Institute for Global Health (UNU-IIGH), Jalan Yaacob Latiff, 56000 Cheras, Kuala Lumpur, Malaysia.

[7] South East Asia Community Observatory (SEACO), Segamat, Johor, Malaysia.

[8] School of Life Sciences, The University of Nottingham Medical School, Nottingham, United Kingdom.

*Corresponding author: Dr Andrea McGrattan; Institute of Health and Society, and Institute of Ageing; Newcastle University, Newcastle, United Kingdom; andrea.mcgrattan@newcastle.ac.uk ; +44 (0) 191 208 5467

The material presented in this manuscript is original and it has not been submitted for publication elsewhere while under consideration by BMJ Open

Abstract Word Count: 291

Main Text Word Count: 5,207

References: 49

Tables: 2

Figures: 1

Online supplementary material: 0

For peer review only - http://bmjopen.bmj.com/site/about/guidelines.xhtml

For peer review only

ABSTRACT

Introduction

Global population ageing is one the key factors linked to the projected future rise of dementia incidence. Hence, there is a clear need to identify strategies to overcome this expected health burden and have a meaningful impact on populations' health worldwide. Current evidence supports the role of modifiable dietary and lifestyle risk factors in reducing the risk of cognitive decline. In South-East Asia, changes in individual eating and lifestyle patterns under the influence of westernised habits, have resulted in significant increases in the prevalence of metabolic, cardiovascular and neurodegenerative non-communicable diseases (NCDs). Low vegetable consumption and high sodium intake have been identified as key contributors to the increase in the prevalence of NCDs in these countries. Therefore, nutritional and lifestyle strategies targeting these dietary risk factors are warranted in these populations to prevent cardio-metabolic and neurodegenerative chronic diseases.

Methods and Analysis

The primary aim of this randomised 2*2 factorial trial is to demonstrate the feasibility, acceptability and the potential to deliver a dietary intervention to increase the consumption in high-nitrate green leafy vegetables and reduce salt intake over a 6-month period among Malaysian adults with raised blood pressure. Additional secondary outcomes will be collected including blood pressure, cognitive function, body composition, physical function and biological samples to assess adherence to the dietary intervention. A subgroup of participants will also complete post-intervention focus groups to further explore the feasibility considerations of executing this research, the ability of these individuals to make changes to their diet as well as the barriers and facilitators associated with implementing dietary change.

Ethics and Dissemination

Ethical approval has been obtained from MUHREC (Monash University Human Research Ethics Committee) and MREC (Medical Research and Ethics Committee) of Malaysia. Results of the study will be disseminated via peer-reviewed publications and presentations at national and international conferences. Trial Registration Number: ISRCTN47562685

For peer review only - http://bmjopen.bmj.com/site/about/guidelines.xhtml

ARTICLE SUMMARY

Strengths and limitations of the study

1. First ever 2*2 trial testing single and combined effects of dietary nitrate and salt reduction for the prevention of cognitive decline in developing countries
2. Longest high-nitrate based intervention ever conducted
3. Effective use of population based cohort as a sampling platform for recruitment into clinical trials
4. Use of objective biomarkers for the assessment of adherence to dietary intervention and possibility of internal validation of dietary assessment methods
5. Mixed-method approach to obtain key information on feasibility of study
6. Owing to the small sample size and the feasibility nature of this study, the planned comparative analysis is only exploratory, and therefore, the efficacy of the intervention between groups cannot be determined. However, the results of this study will be instrumental in the design and calculation of the sample size of a larger, follow-on efficacy trial

INTRODUCTION

Dementia is an incurable progressive syndrome characterised by multiple cognitive deficits and loss of independence, and its pathogenesis is linked to multiple distinct neuropathological processes including Alzheimer's disease (AD) and/or vascular pathology.[1] Observational evidence suggests that several of the pathological processes underlying dementia might be delayed or prevented by interventions focussed on dietary and behavioural changes[2]. Hence, in the absence of definite pharmacological treatments, these options represent a key strategy to alleviate its individual and social impact.[3] Optimal control of modifiable cardiovascular factors such as blood pressure, as well as adherence to lifestyle recommendations such as diet and physical activity, have been consistently associated with reduced dementia risk in observational studies.[2-4] The role of nutrition for the prevention of cognitive decline in cognitively healthy individuals and populations with cognitive impairment has been extensively investigated by testing the role that single nutrients, such as n-3 PUFA or B vitamins[5] as well as whole dietary patterns, such as the DASH diet[6], the Mediterranean diet[7 8] and the Mediterranean-DASH diet Intervention for Neurodegeneration Delay (MIND diet).[7 9] There are a small number of RCTs available to support the effect of diet on cognitive function in Mild Cognitive Impairment[10] and among those at risk of cognitive decline or cognitively healthy populations[11]; however the trials have a small sample size and overall modest quality. This body of research evidence has led to the development of risk reduction guidelines for cognitive impairment

For peer review only - http://bmjopen.bmj.com/site/about/guidelines.xhtml

and dementia by the World Health Organisation (WHO) which promotes physical activity and healthy diets rich in fruits, vegetables, wholegrains and reduced salt content. [12]

The prevalence of people with dementia in low and middle income countries (LMICs), particularly in Asia, is rising and is estimated to increase from 60% in 2001 to 71% by 2040.[13] Malaysia is one of many Asian countries experiencing rapid socio-economic and nutritional transitions[14]. Changes in individual eating and lifestyle patterns under the influence of westernised habits, as well as a rise in food availability and financial prosperity of the population, have resulted in significant increases in the prevalence of chronic metabolic, cardiovascular and neurodegenerative non-communicable diseases [NCD][15]. Implementation of effective public health interventions for the prevention of dementia is challenging in countries with limited social and health care resources which are unable to meet the growing demands of ageing populations. Therefore, there is an urgent need to identify protective and risk factors for dementia and design and test the efficacy of interventions for the early prevention of cognitive decline and dementia.

Healthy dietary patterns, such as Mediterranean Diet or Dietary Approach to Stop Hypertension (DASH Diet), have been linked in epidemiological studies[16-21] and a large-scale clinical trial[8 22] to improved cognitive function and reduced dementia risk. These two dietary patterns are characterised by increased consumption of fruit, vegetables and avoidance of “high-risk” foods with high fat, refined sugar and salt content. There is a need however to test ‘complex’ multi-domain strategies targeting different biological mechanisms as well as facilitate the adoption and sustain dietary and lifestyle changes.[2 23] Effective interventions for the prevention of cardiovascular and type 2 diabetes support the efficacy of multi-domain interventions and the recent FINGER (The Finnish Geriatric Intervention Study to Prevent Cognitive Impairment and Disability) trial has successfully pioneered such an approach for dementia prevention.[24 25]

The key nutrients associated with the beneficial effects of increased fruit and vegetables consumption within these dietary patterns are difficult to disentangle, but recent analyses have identified the high content of inorganic nitrate as a potential nutrient involved the improvement of vascular and metabolic health.[26] Inorganic nitrate is closely linked to the metabolism of Nitric Oxide (NO) which is known for its multiple effects on physiological function such as control of blood pressure, brain function and immunity.[27] An increase in inorganic nitrate consumption has been associated with an increase in NO production via complex mechanisms involving oral microbiota, gastric environment and activation of enzymes with a capacity to reduce nitrate into nitrite first, and then finally into NO[28].

For peer review only

Recent evidence from epidemiological studies and clinical trials have demonstrated the association of high-nitrate vegetables and increased nitrate intake with improved blood pressure, metabolic health and cognitive function.[29 30] Beetroot and green leafy vegetables, such as spinach, lettuce and rocket, are a key source of naturally available inorganic nitrate, containing over 250 mg of nitrate per 100 g of fresh vegetable weight.[31] A systematic review and meta-analysis demonstrated that inorganic nitrate and beetroot juice supplementation were associated with a significant decrease in blood pressure. The pooled effect for both interventions showed a reduction in systolic blood pressure of 4.4 mmHg, with a more modest decrease of 1.1 mmHg in diastolic blood pressure.[32] However, the results on cognition are still preliminary, with studies of short intervention durations and among small samples,[27] and studies conducted in developing countries are also limited. Nevertheless, with hypertension highlighted as an important modifiable risk factor for dementia,[3 12] more studies that test the implementation of dietary interventions that aim to improve blood pressure are warranted.

Similarly, these healthy dietary patterns are generally characterised by a low consumption of salt, which is mostly derived as a result of consumption of foods with low salt content, as well as a consumption of fresh, unprocessed food products[33]. The DASH diet has been specifically developed around healthy dietary advice in order to promote consumption of a healthy dietary choices alongside a strict control of sodium intake[34]. Secondary analyses derived from seminal DASH studies showed additional benefits on blood pressure and cardio-metabolic health in individuals with lower salt intake and adherence to a healthy dietary pattern[35]. These analyses have been extended to cognitive function with promising results,[19 21] but the evidence has mostly come from studies conducted in the United States and no studies to our knowledge have been conducted in LMICs. Sodium is the main electrolyte contained in extracellular fluids and involved in regulation of blood pressure and cellular activities. However, an excess sodium intake has been linked to extracellular volume expansion resulting in increased blood pressure and renal sodium excretion.[36] Epidemiological studies have repeatedly proven the association of high salt diets with impaired blood pressure control and increased cardiovascular risk;[37 38] this evidence has been confirmed in several clinical trials testing the effects of salt-reducing intervention and demonstrating the protective effects of salt reduction on cardiovascular health.[39 40] Excessive sodium consumption is recognised as a global health issue[41] with efforts aiming to reduce salt intake at the population level by 30% by 2025, and 75 countries have already implemented national sodium reduction programmes to help meet this target.[42] The strategies used to reduce sodium intake at the population level have used a variety of approaches, to include working with industry to reformulate food products, establishing sodium content targets for foods, revising food labelling schemes, taxation of high-salt foods and development of educational

For peer review only

For peer review only - http://bmjopen.bmj.com/site/about/guidelines.xhtml

interventions.[43] In sum, an increased consumption of dietary nitrate and lower salt intake may therefore have additional benefits on health outcomes such as blood pressure and cognition, mediated by an increase in NO generation (i.e., nitrate) and control of vascular tone and cellular metabolism (i.e., sodium). However, this hypothesis has never been tested in previous interventions which represents the main aim of the research study proposed in this protocol.

The Global Health Dementia Prevention and Enhanced Care (DePEC) project is a National Institute for Health Research (NIHR) funded research study with a key goal to reduce the personal and societal burden of dementia, by developing approaches to reduce future numbers of people developing dementia and develop more efficient post-diagnostic care. The project brings together: dementia experts in public health/primary care (Newcastle University), global epidemiological 10/66 dementia research group from King's College London (KCL), with a strong existing LMICs network and new LMICs partners, one from each economic category (upper LMIC-Malaysia, LMIC-India and least developed Tanzania). The DePEC-Nutrition study (presented here) is one of five work streams within the overall DePEC project. In preparation for the DePEC-Nutrition study, we have recently conducted a systematic review on the effects of nutritional interventions on cognitive function in South-East Asian countries[11]. To date, 22 randomised controlled trials investigated the effect of nutritional interventions on cognitive performance in developing economies of East Asia. The quality of the studies was mixed. Four different types of interventions could be identified, namely interventions based on: micronutrient supplementation; nutritional and lifestyle counselling; essential fatty acids supplementation; and chicken essence supplementation. The majority of the studies showed some significant beneficial effects of the interventions on cognitive performance. However, these effects were not all consistent across all of the neuropsychological tests used in each study, and, as a result, significant beneficial effects for a specific intervention were often not seen in meta-analysis. No studies used complex interventions focused on promotion of healthy dietary patterns and no studies has tested the effects of either nitrate or salt reduction on cognitive function either alone or combined. This therefore provides rationale in support of the conduction of the pilot feasibility study presented in this protocol.

Purpose and Aims

The overall objective of the DePEC-Nutrition study is to demonstrate the feasibility, acceptability and the potential to deliver a dietary intervention to increase the consumption in high-nitrate green leafy vegetables and reduce salt intake over a 6-month period among Malaysian adults with raised blood pressure. The objectives focus on the core areas of an intervention study that need to work for the

For peer review only - http://bmjopen.bmj.com/site/about/guidelines.xhtml

study to succeed procedurally and gather information on the effect size of the intervention on cognition and blood pressure, which will be instrumental in the design and calculation of the sample size of a follow-on efficacy trial.

METHODS AND ANALYSIS

Study design

The DePEC-Nutrition study is a 6 month randomised 2*2 factorial trial including four parallel arms: (1) control, (2) high-nitrate vegetable consumption, (3) low salt consumption and (4) combined high-nitrate vegetable consumption plus low salt consumption. The trial will be conducted among 120 middle-aged and older Malaysian adults. Sponsor and grant number: GHR Group: 16/137/62 – NIHR Global Health Research Group on Dementia Prevention and Enhanced Care (DePEC), Newcastle University, United Kingdom. This trial has been registered with ISRCTN (ISRCTN47562685). The SPIRIT reporting guidelines were used to guide the preparation of this protocol.[44]

Participants

This study is embedded within the South East Asia Community Observatory (SEACO) population (http://www.seaco.asia/). SEACO is a health and demographic surveillance site (HDSS); a unique research platform in population health and wellbeing focused on a middle-income community in Segamat, Johor in the southern tip of the Peninsular Malaysia. The district of Segamat comprises urban, rural and plantation areas with an ethnic mix including Malays, Chinese and Indian, close to national proportions. SEACO runs its HDSS research platform in five sub-districts (mukim) of the Segamat district: Bekok, Chaah, Gemereh, Jabi and Sungai Segamat. The SEACO population was established by a baseline census (March 2012 to February 2013) that included information on sociodemographic status and self-reported health conditions. Participants underwent detailed health profile assessment in the first health round in 2013-2014. The latest census was done in 2017, with a total number of 40,015 participants completing the assessment. Individuals have been re-seen after a year (in 2018) for the second health round, which collected a detailed health profile of all the consenting participants. Individuals aged 50 years and over, who are living in Segamat, Johor and have been assessed as part of SEACO in its previous health round will form the study population for the DePEC-Nutrition study. The baseline assessment will be carried out in the Klink Kesihatans (KK) in Sungai Segamat sub–district (a community health clinic in Segamat, Johor). Hence, only participants from within 5 km radius of KK Segamat will be included in the study population.

For peer review only - http://bmjopen.bmj.com/site/about/guidelines.xhtml

Recruitment

The community will be informed about this current study before it is initiated. Participants will be randomly identified by the application of the inclusion and exclusion criteria to the SEACO health surveillance database. Potential participants will be approached via home visit and will be provided with information about the study. They will be given an opportunity to read an information sheet, containing detailed information about the study procedures and will have the opportunity to ask questions. Thereafter, their interest in participating to the study will be evaluated. If they agree to take part, a screening assessment will be completed. If eligible, participants will be invited to attend a baseline study visit at the health facilities within the community.

Eligibility criteria

To be eligible for inclusion in this study, participants must report as:

1. Male and female participants with an age between 50 and 75 years from the participants of SEACO.
2. Pre-hypertensive, stage 1 hypertensive or diagnosed hypertensive: Any person who has a self-reported history of hypertension (on or not on medication) or those who had a blood pressure recording of systolic blood pressure 120-159 mmHg or diastolic blood pressure 80-99 mmHg.
3. Not severely cognitively impaired (defined as a Mini Mental Examination Status [MMSE] score of 19 or over).

Participants will be excluded from the study if they:

1. Are participating in other research clinical studies
2. Are unable to provide consent
3. Have systolic blood pressure more than or equal to 160 mmHg and /or diastolic blood pressure more than or equal to 100 mmHg
4. Adhere to any therapeutic diet such as weight loss treatments or a gluten-free diet
5. Are strictly vegan (likely to have a high intake of vegetables). Since Indian participants are more likely to follow a vegetarian or semi-vegetarian diet, in order to maintain representativeness across ethnic groups included in the study, only participants on strictly vegetarian diet (i.e., vegans) will be excluded from the study
6. Participants with a body mass index (BMI) less than 18.5 kg/m^2
7. Have a history of active cancer or any diagnosis of cancer in the last 5 years
8. Have a history of excessive alcohol intake (>21 units of alcohol per week)

For peer review only - http://bmjopen.bmj.com/site/about/guidelines.xhtml

For peer review only

9. Have a history of brain damage, significant head trauma (including loss of consciousness as a result)
10. Have a diagnosis of acute and chronic medical conditions interfering with the study outcomes such as systemic infections (tuberculosis, hepatitis B, HIV/AIDS), severe liver and kidney diseases, inflammatory bowel diseases, coronary heart diseases, cerebrovascular diseases or diabetes on insulin therapy
11. Have had any major surgical procedures (in the past six weeks or planned in next one year) that could interfere with the study outcomes
12. Have current diagnosis of moderate or severe depression, or other serious mental or brain disorder and currently on psychiatric medication (antidepressants, sedatives, antipsychotics)
13. Have systemic use of sodium-altering drugs (angiotensin-converting-enzyme (ACE) inhibitors, angiotensin receptor blockers (ARB), corticosteroids, diuretics, hormonal therapies (oestrogens, thyroxin, and progesterone), non-steroidal anti-inflammatory drugs (NSAIDs), proton-pump inhibitors), organic nitrates
14. Have a change in anti-hypertensive medication regimen for the previous 3 months
15. Have limited mobility due to any reason
16. Are planning to move house in next one year

Study procedure

Data collection will be recorded directly on an electronic hand held device (Samsung Galaxy Tab 3v – Survey CTO). Questionnaire items will be read out-loud by the data collector and participant responses will be recorded directly onto the electronic tablet. The flow of participants through the DePEC-Nutrition study is illustrated in Figure 1. At the screening visit (Visit 1), written informed consent will be first obtained, after which participants will complete a screening questionnaire to collect information on demographics, co-morbidities, medication history, cognitive function (MMSE), body weight, height and resting blood pressure. If eligible, participants will attend a baseline measurement appointment at Klinik Kesihatan, Segamat (Visit 2) and undergo their baseline assessments, to include: dietary assessment (24-hour recall and food frequency questionnaire (FFQ)), cognitive assessment and collection of biological samples (blood, dried blood spots, 24-urine and spot urine samples, saliva samples, and salivary strips). An interim two-month visit (Visit 3) will take place for those in the intervention groups only, to discuss behavioural strategies and with the aim to enhance compliance to the intervention. In addition, a brief adherence questionnaire will be administered, body weight, resting blood pressure will be measured and salivary strip and spot urine samples will be collected. At an interim four-month visit (Visit 4), resting blood pressure and body weight will be measured and a

For peer review only - http://bmjopen.bmj.com/site/about/guidelines.xhtml

short adherence questionnaire will be administered for the participants allocated to the intervention arms. Salivary strip and spot urine samples will also be collected at Visit 4. Participants in the control arm will have their weight and blood pressure measured at visit four only. The visit will also provide the opportunity to remind participants of the importance of attending the final study visit. The end of study visit (Visit 5 at Klinik Kesihatan, Segamat) will include the conduction of the measurements collected at baseline (Visit 2), including cognitive assessment, dietary assessment, and collection of biological samples (blood, dried blood spots, 24-urine and spot urine samples, saliva samples, salivary strips). In addition, an exit group session (focus group evaluation) and a self-administered feedback questionnaire will be scheduled to evaluate the adherence to the interventions and obtain detailed feedback on the overall study protocol.

Randomisation and blinding

Block randomisation will be carried out to assign eligible participants into one of the four arms. Block sizes of four participants will be used in randomisation and will be generated by a member of the research team not involved in the data collection. The interviewers will be blinded to the participant assignment to intervention arms. Participant blinding to the intervention allocation will not be possible due to the nature of the dietary intervention study.

Intervention and control arms

This dietary and behavioural intervention will focus on key components that are related to changing local dietary patterns influenced by nutritional transition trends in Malaysia. It aims to target two key components that have protective (dietary nitrate) and negative (salt intake) effects on cardiovascular and cognitive health. The intervention will target individuals with a higher CVD risk to adopt a tailored strategy to enhance daily consumption of green leafy vegetables and / or reduce salt intake. Health education counselling sessions with a nutritionist or medical doctor will be conducted at baseline to provide sustainable and effective strategies to improve dietary habits as well as monitor and address key barriers to changes, and then to monitor compliance in group and individual sessions during the intermediate visits (2 and 4 months, respectively). All intervention groups will receive written information focussed on health benefits, sources, recommended intakes and practical tips related to one of the following dietary interventions; (1) to increase dietary nitrate (2) to reduce salt intake (3) to increase dietary nitrate and to reduce salt intake. Those who are randomised to the salt intervention (either alone or combined) will receive a measuring spoon as an additional strategy to support individuals to understand portion size and measure salt intake, as successfully tested by an intervention study in China.[45] The intervention groups will also receive bi-weekly text message

For peer review only - http://bmjopen.bmj.com/site/about/guidelines.xhtml

reminders to provide reinforcement of the key dietary behaviour change messages relevant for their group allocation. Those allocated to the control group (4) will receive written information on general healthy eating and lifestyle advice based on the Malaysian dietary guidelines.[46]

Outcome measures

Outcome assessments will be conducted at baseline, month 4 and month 6 for all groups, with an additional assessment visit at month 2 for those in the intervention groups. Assessments at baseline and month 6 will be conducted in clinic, with remaining assessments completed in the participants own home. The same instruments will be used for measurements at each time point. Assessors are trained prior to the assessments to follow a standardised protocol. A timeline of the study procedures can be found in Table 1.

For peer review only

Table 1: Timeline of study procedures

**Procedures**	**Consent/Screening**	**Baseline Week 1**	**Week 2**	**Week 4**	**Week 6**	**Week 8**	**Week 10**	**Week 12**	**Week 16**	**Week 18**	**Week 20**	**Week 22**	**End of study Week 24**
**Inclusion/Exclusion**	✓												
**Consent**	✓												
**Activities for intervention group**													
Dietary education		✓											
Reinforcement at home visit		✓				✓			✓				
Group education counselling session		✓											
Home visit						✓			✓				
SMS messages			✓	✓	✓	✓	✓	✓	✓	✓	✓	✓	✓
**Activities for control group**													
General health promotion message		✓											
Home visit									✓				
**Study Measurements**													
Sociodemographic		✓											
Concomitant medication check list		✓				✓							✓
Any medical events		✓				✓							✓
Physical activity (PASE)		✓											✓
Depression (GDS)		✓											✓
Food frequency questionnaire (FFQ)		✓											✓
24hr dietary recall		✓											✓
Nitrate: Urine		✓**a**				✓***b**			✓**b**				✓**a**
Salt: Urine		✓**a**				✓***b**			✓**b**				✓**a**
Cognitive performance		✓											✓
Resting blood pressure		✓				✓*			✓				✓
Body composition		✓				✓***c**			✓**c**				✓
Physical function		✓											✓
Blood Sample and dried blood spot		✓											✓
Saliva sample and salivary strips		✓				✓*‡			✓‡				✓

a: 24-urine and spot urine samples; b: spot urine sample only; c: body weight only; * intervention groups only; ‡ saliva strips only

For peer review only - http://bmjopen.bmj.com/site/about/guidelines.xhtml

Primary outcome

The primary outcome measures are related to feasibility and will be collected via questionnaire, review of the study procedures and by review of data collector field notes. Patient feedback, acceptance and adherence issues will be additionally assessed through qualitative focus groups conducted post-intervention. The specific feasibility considerations that will be assessed in the study have been outlined in Table 2.

Table 2: Primary outcome: Feasibility considerations to be assessed

**Feasibility Considerations**	
**Recruitment**	1. Recruitment rates<br>2. Time taken for the recruitment of participants<br>3. Characteristics of recruited sample as against expected<br>4. Non-response rates<br>5. Reasons for non-response
**Retention and Completion**	1. Retention rates<br>2. Number of drops and reasons for withdrawal<br>3. Follow-up response rates (2 months and 4 months follow-ups)
**Data Collection methods**<br>Feasibility of measurement tools	1. Time taken to fill in questionnaires<br>2. Missing data from questionnaires<br>3. Understanding level of procedures and measurement protocols<br>4. Suitability of outcome measures<br>5. Sensitivity of outcome measures to change<br>6. Internal consistency of outcome measures - validation of data collection methods (24 hours vs spot urine; saliva samples vs salivary strips)
Resource Capacity	1. Data collection and measurement equipment<br>2. Field support (transportation, IT, etc.)<br>3. Personnel (were the right numbers of people with the right skills available when they were needed)<br>4. Duration of each phase
**Acceptability and adherence to intervention**	1. Barriers to participation (collected at screening interview)<br>2. Burden (reasons for not taking part/discontinuation or dropping out)<br>3. Qualitative enquiry/feedback from participants and research staff<br>4. Focus group evaluation - This part of the feasibility trial will separately examine the acceptability and suitability of: intervention materials; intervention timing; intervention procedures; measurement and sample collection; adherence to intervention and barriers and facilitators to dietary and lifestyle change.

For peer review only - http://bmjopen.bmj.com/site/about/guidelines.xhtml

Secondary outcomes

Cognitive assessment

A validated version of Montreal Cognitive Assessment test (MoCA), auditory verbal learning test, categorical verbal fluency and trail making test (TMT) part B will be conducted at baseline and end of study visits.

Blood pressure

Three consecutive measurements of resting blood pressure readings will be recorded in sitting position using a calibrated OMRON monitor. The median measure will be used to estimate the blood pressure. Blood pressure will be measured at all outcome assessment visits.

Anthropometry and body composition

Body weight will be recorded in kilograms using calibrated, electronic scales to the nearest 0.1kg where possible. Weight will be measured at all outcome assessment visits. Height will be recorded in metres using a stadiometer and measured to the nearest 0.01m where possible. The recordings for weight and height will be subsequently used to calculate BMI by dividing weight (kg) by height (m^2). BMI will be calculated at baseline and end of study visits. Body composition will be measured by bioelectrical impedance. A Tanita Body Composition analyser will be used to measure body fat percentage and classification, segmental subcutaneous fat and skeletal muscle percentage (whole body, trunk, legs and arms), resting metabolism, visceral fat level and classification and body age. Body composition will be measured at baseline and end of study visits.

Physical assessments

The following physical assessments will be conducted at baseline and end of study visits. **(1)** Muscle strength will be measured by a hand grip-strength dynamometer. Using their right arm, the participant will be asked to make three measurements and an average calculated. The same process will be repeated for the left arm. **(2)** A 4 meter walk test will be done to assess the gait speed. Two trials will be conducted and the average measure of time in minutes will be used to assess the gait speed. **(3)** In the Timed Up and Go (TUG) test, the time taken by participants while rising from an armless chair (46 cm height), for walking 3m, turning, walking back and sitting down will be measured. The TUG test will be performed twice consecutively, and the average of the two scores will be used. **(4)** The Geriatric Depression Scale (GDS) (short form) will be used to assess depressive symptoms[47] – scores range from zero to 15, with a score of zero to five indicating a normal score and a score greater than five

For peer review only

For peer review only - http://bmjopen.bmj.com/site/about/guidelines.xhtml

suggesting depression. The international physical activity questionnaire (IPAQ)[48] will be used to assess physical activity.

Dietary Assessment

Dietary assessment data will be collected by two methods: 24 hour recall and FFQ. Participants will complete a one day 24 hour recall facilitated by a trained data collector. The FFQ is a validated questionnaire used in previous dietary research in Malaysia.[49] Information will be entered into Nutrition Pro Software (v7.5) and daily intake will be calculated.

Biological Sample Collection

The following bio-specimen will be collected in this study

1. Whole venous blood (15 ml)
2. 24 hour urine sample
3. Spot urine sample
4. Saliva
5. Capillary blood

All samples will be used only for the purpose of the study. The venous blood will first be centrifuged, separated into aliquots and then stored at -20°C at the Klinik Kesihatan Segamat; and then transported to Monash University Sunway campus for storage. Other samples will be stored at -20°C storage. The freezer room for the stored samples will be under lock and key with restricted access.

Blood sampling

Whole blood samples will be analysed to measure changes in nitrite concentrations during the intervention. Samples will also be analysed to assess for biomarkers of cardio-vascular risk (C-reactive protein, glycated haemoglobin, nitro-tyrosine) and direct brain measures such as plasma BDNF, plasma amyloid β42 and amyloid β40. A capillary blood sample will be taken and analysed for the concentration of glucose in the blood using a portable glucometer. Dried blood spot samples will be analysed to measure changes in nitrate concentrations during the intervention. Blood spots will be first processed using a standardised elution protocol to obtain a liquid solution of the samples and then analysed for nitrate concentrations using ozone-based chemiluminescence, which is the reference method for the analysis on nitrate in biological fluids. All blood sampling assessment methods will be conducted at baseline and end of study visits.

For peer review only

For peer review only - http://bmjopen.bmj.com/site/about/guidelines.xhtml

24 hour and spot urine sampling

Urinary sodium will be determined using the ion-selective electrode method. For nitrate, samples will be diluted 1:100 and then analysed for nitrate concentrations using ozone-based chemiluminescence, which is the reference method for the analysis on nitrate in biological fluids.

Saliva sampling

To measure nitrate, samples will be diluted 1:100 and then analysed for nitrate concentrations using ozone-based chemiluminescence. For the salivary strips, a dedicated app freely available to download on mobile devices will be used to provide a quantitative reading of the salivary nitrite concentrations as a surrogate marker of dietary nitrate intake.

Patient and public involvement - Post-intervention qualitative evaluation

Although participants will not be involved in the recruitment and conduct of this study, participants will be invited to provide feedback on their participation intervention study via focus groups discussions. The focus group topic guide will also be piloted among a random sample of participants prior to implementation to allow feedback, refinement and tailoring prior to implementation. Two focus group discussions will be conducted within each arm of trial (total 8). Purposive sampling will be used to capture broad variation in age, ethnicity, house location and for better understanding of the phenomenon being studied. All focus groups discussions will be audio recorded and the topics covered will include: **(1)** Access to the food items - financial (affordability), physical factors; **(2)** Cultural acceptability of the recommended food items; **(3)** Family support to adhere to the dietary changes; **(4)** Adherence to the instructions - facilitators and barriers, methods to self-monitor; **(5)** Perceived benefit and side effects of intervention; **(6)** Study context - education material, frequency of visit, location of visits, data collection methods and tools; **(7)** Provision of biomedical samples - barriers, cultural acceptability, difficulty; **(8)** Likes and dislikes to the new dietary options and **(9)** Preference to the trial arm. This qualitative element will allow the feedback and recommendations from participants to directly inform the refinement of a follow-on efficacy trial.

Sample size

A formal sample size calculation has not been performed considering the pilot, feasibility nature of the study as well as the qualitative nature of the primary outcomes focussed on retention rates to the trial and adherence to the interventions. A sample size of 30 per group was based on the estimated effect size provided by the guidelines by Whitehead et al[50], which outlines the sample size calculation for pilot studies with the aim to maximise resources and avoid occurrence of a type II error. Specifically, a sample size greater than 25 individuals per group would provide in a 90% powered main

For peer review only - http://bmjopen.bmj.com/site/about/guidelines.xhtml

trial the ability to detect a small effect size between 0.1 and 0.3. More than 100 participants are expected to complete the trial; i.e., an anticipated drop-out rate ≤20%.

Statistical Analysis

The main aims from the feasibility study are to show that the two interventions can be followed with high adherence during the trial. Secondary aims are largely descriptive, aiming to provide bounds for key parameters to inform the main trial. The recruitment and retention rate in each arm will be described. It is unlikely that statistically significant differences in retention rates will be discovered, but upper and lower bounds will be estimated, and reasons for groups with apparently low recruitment or retention rates will be explored qualitatively. Normality of the distribution of the variables and appropriate transformations (LogX, 1/X, Xn) will be performed if necessary. Summary data will be expressed as mean (SD) or frequency (percent). General linear models for repeated measures will be used to detect significant differences between the intervention groups with and without adjustment for baseline levels. Chi square test will be used for categorical variables. An interaction term (time x group) will be built to assess between-group interactions in changes in the measured outcomes during the interventions. Dietary data will be analysed using the Nutritionist Pro software (v7.5).

Data collection supervision and training

SEACO is an ISO certified research platform for its operations and hence training and supervision are carried out in accordance with SEACO's standard operating procedures. All data collectors are required to be fluent in the local language used for interview (Bahasa Melayu or Malay language or Mandarin) and have at least a working knowledge of English. To ensure data quality and consistency across interviewers, the field supervisor will undertake random concurrent supervisory visits to observe practice. The data collectors will also be periodically observed by the project leader and the field manager. Based on the scoring completed during these observation sessions, onsite training or retaining will be carried out as appropriate. Data collection is undertaken on Samsung Galaxy tablets. The data are encrypted on the tablet to ensure they are secure and inaccessible in the unlikely event that they are mislaid by data collectors. Transfer of data from the tablet to the remote server is done on a weekly basis. The data are also encrypted on the server and backed up by servers at Monash Sunway Campus and at Monash Clayton. Research staff have limited access to anonymised data.

For peer review only - http://bmjopen.bmj.com/site/about/guidelines.xhtml

411 **Data monitoring**

412 All expected and unexpected adverse events reported by participants will be recorded in an events
413 register and reported to the Human Research Ethics Committee. Due to the nature of the treatment
414 products, i.e. commercially available food products, no adverse events are expected. However, if
415 participants feel in anyway adversely affected by any foods or the principal investigator feels an
416 adverse event necessitates cessation, the participant would be advised not to continue and the
417 appropriate measures will be taken (i.e. record in field notes, contact research nurse and principal
418 investigator if deemed necessary).

419

420 **Ethics and Dissemination**

421 The procedures outlined in this protocol, pertaining to the conduct, evaluation, and documentation
422 of this study, are designed to ensure that the sponsor and investigator abide by GCP Guidelines and
423 under the guiding principles detailed in the Declaration of Helsinki. DM and YCS have obtained GCP
424 certification from Ministry of Health, Malaysia. Ethical approval has been granted by Monash
425 University Human Research Ethics Committee (MUHREC) (17864) and the Malaysian Medical Research
426 Ethics Committee (MREC) (#NMRR-19-617-45916). Results of the study will be disseminated via peer-
427 reviewed publications and presentations at national and international conferences.

428

429 **DISCUSSION**

430 This study will demonstrate the feasibility of a dietary intervention to increase the consumption in
431 high-nitrate green leafy vegetables and reduce salt intake over a 6-month period among Malaysian
432 adults with raised blood pressure. The design of this feasibility study has taken into account the
433 evidence collected in developed countries on the efficacy of complex interventions for the prevention
434 of cognitive decline and evidence on the major transitions in dietary patterns occurring in developing
435 countries as a result of nutrition transition trends. An increased consumption of vegetables and
436 reduction of salt intake are key dietary recommendations for the prevention of cardio-metabolic
437 diseases. Our hypotheses are that a targeted, educational approach, to encourage healthy dietary
438 choices focused on these key elements of the diet, would be linked to greater long-term adherence to
439 the interventions and improved cognitive function with prospective, projected reduced risk of
440 dementia in these populations. This mixed-method feasibility study will provide key quantitative and
441 qualitative information on the delivery of a novel dietary intervention and will aim to estimate the
442 effect size of the single and combined interventions on cognitive function and blood pressure, which
443 will be instrumental in the design and calculation of the sample size of a larger, follow-on efficacy trial.

444

For peer review only - http://bmjopen.bmj.com/site/about/guidelines.xhtml

AUTHOR CONTRIBUTORS

MS, DDR and DM were involved in conception and trial design. MS, AMG and AMH contributed to the intervention development and design. AMG wrote the initial manuscript draft and MS and DM contributed to writing the final manuscript and provided critical comments during revisions. All authors contributed to a critical review of the paper. DM and CPW will be responsible for overseeing the recruitment, data collection and intervention delivery.

FUNDING

Sponsor and grant number: GHR Group: 16/137/62 – NIHR Global Health Research Group on Dementia Prevention and Enhanced Care (DePEC), Newcastle University, United Kingdom. Sponsor - Monash University, Malaysia Jeffrey Cheah Sch of Med & HS, Selangor, 47500, Malaysia. Tel: +60 (0)7 2190 600 / Institute of Health and Society, and Institute of Ageing; Newcastle University, Newcastle, United Kingdom.

COMPETING INTEREST STATEMENT

"All authors have completed the ICMJE uniform disclosure form at www.icmje.org/coi_disclosure.pdf and declare: no support from any organisation for the submitted work; LR reports grants from National Institute of Health Research Senior Investigator award during the conduct of the study; no other relationships or activities that could appear to have influenced the submitted work."

For peer review only - http://bmjopen.bmj.com/site/about/guidelines.xhtml

REFERENCES

1. Robinson L, Tang E, Taylor J-P. Dementia: timely diagnosis and early intervention. *BMJ : British Medical Journal* 2015;350:h3029. doi: 10.1136/bmj.h3029
2. Scarmeas N, Anastasiou CA, Yannakoulia M. Nutrition and prevention of cognitive impairment. *The Lancet Neurology* 2018;17(11):1006-15. doi: https://doi.org/10.1016/S1474-4422(18)30338-7
3. Livingston G, Sommerlad A, Orgeta V, *et al.* Dementia prevention, intervention, and care. *The Lancet* 2017;390(10113):2673-734. doi: https://doi.org/10.1016/S0140-6736(17)31363-6
4. Norton S, Matthews FE, Barnes DE, *et al.* Potential for primary prevention of Alzheimer's disease: an analysis of population-based data. *The Lancet Neurology* 2014;13(8):788-94. doi: https://doi.org/10.1016/S1474-4422(14)70136-X
5. Forbes SC, Holroyd-Leduc JM, Poulin MJ, *et al.* Effect of Nutrients, Dietary Supplements and Vitamins on Cognition: a Systematic Review and Meta-Analysis of Randomized Controlled Trials. *Can Geriatr J* 2015;18(4):231-45. doi: 10.5770/cgj.18.189
6. Smith PJ, Blumenthal JA, Babyak MA, *et al.* Effects of the dietary approaches to stop hypertension diet, exercise, and caloric restriction on neurocognition in overweight adults with high blood pressure. *Hypertension (Dallas, Tex : 1979)* 2010;55(6):1331-38. doi: 10.1161/HYPERTENSIONAHA.109.146795 [published Online First: 2010/03/19]
7. McEvoy CT, Guyer H, Langa KM, *et al.* Neuroprotective Diets Are Associated with Better Cognitive Function: The Health and Retirement Study. *J Am Geriatr Soc* 2017;65(8):1857-62. doi: 10.1111/jgs.14922 [published Online First: 2017/04/25]
8. Valls-Pedret C, Sala-Vila A, Serra-Mir M, *et al.* Mediterranean Diet and Age-Related Cognitive Decline: A Randomized Clinical TrialMediterranean Diet and Cognitive DeclineMediterranean Diet and Cognitive Decline. *JAMA Internal Medicine* 2015;175(7):1094-103. doi: 10.1001/jamainternmed.2015.1668
9. Morris MC, Tangney CC, Wang Y, *et al.* MIND diet associated with reduced incidence of Alzheimer's disease. *Alzheimers Dement* 2015;11(9):1007-14. doi: 10.1016/j.jalz.2014.11.009 [published Online First: 2015/02/11]
10. McGrattan AM, McEvoy CT, McGuinness B, *et al.* Effect of dietary interventions in mild cognitive impairment: a systematic review. *Br J Nutr* 2018;120(12):1388-405. doi: 10.1017/S0007114518002945 [published Online First: 2018/11/09]
11. McGrattan AM, van Aller C, Narytnyk A, *et al.* Nutritional Interventions for the Prevention of Cognitive Impairment and Dementia in Developing Economies of East Asia: Systematic Review and Meta-analysis. Federation of European Nutrition Societies (FENS). Dublin, 2019.
12. WHO. Risk reduction of cognitive decline and dementia: WHO guidelines. Geneva. . World Health Organization;, 2019:Licence: CC BY-NC-SA 3.0 IGO.
13. International. AsD. World Alzheimer Report 2015: The Global Impact of Dementia. London, 2015.
14. Matthew K. The Nutrition Transition in Developing Asia: Dietary Change, Drivers and Health Impacts. In: Jackson P, Spiess WEL, Sultana F, eds. Eating, Drinking: Surviving: The International Year of Global Understanding - IYGU. Cham: Springer International Publishing 2016:83-90.
15. Dans A, Ng N, Varghese C, *et al.* The rise of chronic non-communicable diseases in southeast Asia: time for action. *The Lancet* 2011;377(9766):680-89. doi: https://doi.org/10.1016/S0140-6736(10)61506-1
16. Galbete C, Toledo E, Toledo JB, *et al.* Mediterranean diet and cognitive function: The sun project. *The journal of nutrition, health & aging* 2015;19(3):305-12. doi: 10.1007/s12603-015-0441-z
17. Gu Y, Brickman AM, Stern Y, *et al.* Mediterranean diet and brain structure in a multiethnic elderly cohort. *Neurology* 2015;85(20):1744-51. doi: 10.1212/WNL.0000000000002121

For peer review only - http://bmjopen.bmj.com/site/about/guidelines.xhtml

18. Luciano M, Corley J, Cox SR, *et al.* Mediterranean-type diet and brain structural change from 73 to 76 years in a Scottish cohort. *Neurology* 2017;88(5):449-55. doi: 10.1212/WNL.0000000000003559
19. Wengreen H, Munger RG, Cutler A, *et al.* Prospective study of Dietary Approaches to Stop Hypertension– and Mediterranean-style dietary patterns and age-related cognitive change: the Cache County Study on Memory, Health and Aging. *The American Journal of Clinical Nutrition* 2013;98(5):1263-71. doi: 10.3945/ajcn.112.051276
20. Koyama A, Houston DK, Simonsick EM, *et al.* Association between the Mediterranean diet and cognitive decline in a biracial population. *J Gerontol A Biol Sci Med Sci* 2015;70(3):354-59. doi: 10.1093/gerona/glu097 [published Online First: 2014/07/03]
21. Tangney CC. DASH and Mediterranean-type Dietary Patterns to Maintain Cognitive Health. *Curr Nutr Rep* 2014;3(1):51-61. doi: 10.1007/s13668-013-0070-2 [published Online First: 2014/01/05]
22. Martínez-González MA, Salas-Salvadó J, Estruch R, *et al.* Benefits of the Mediterranean Diet: Insights From the PREDIMED Study. *Progress in Cardiovascular Diseases* 2015;58(1):50-60. doi: https://doi.org/10.1016/j.pcad.2015.04.003
23. Solfrizzi V, Agosti P, Lozupone M, *et al.* Nutritional interventions and cognitive-related outcomes in patients with late-life cognitive disorders: A systematic review. *Neuroscience & Biobehavioral Reviews* 2018;95:480-98. doi: https://doi.org/10.1016/j.neubiorev.2018.10.022
24. Kivipelto M, Mangialasche F, Ngandu T. Lifestyle interventions to prevent cognitive impairment, dementia and Alzheimer disease. *Nature Reviews Neurology* 2018;14(11):653-66. doi: 10.1038/s41582-018-0070-3
25. Ngandu T, Lehtisalo J, Solomon A, *et al.* A 2 year multidomain intervention of diet, exercise, cognitive training, and vascular risk monitoring versus control to prevent cognitive decline in at-risk elderly people (FINGER): a randomised controlled trial. *Lancet* 2015;385(9984):2255-63. doi: 10.1016/s0140-6736(15)60461-5 [published Online First: 2015/03/17]
26. Hord NG, Tang Y, Bryan NS. Food sources of nitrates and nitrites: the physiologic context for potential health benefits. *The American journal of clinical nutrition* 2009;90(1):1-10. doi: 10.3945/ajcn.2008.27131 [published Online First: 2009/05/15]
27. Clifford T, Babateen A, Shannon OM, *et al.* Effects of inorganic nitrate and nitrite consumption on cognitive function and cerebral blood flow: A systematic review and meta-analysis of randomized clinical trials. *Critical Reviews in Food Science and Nutrition* 2018:1-11. doi: 10.1080/10408398.2018.1453779
28. Lundberg JO, Carlstrom M, Weitzberg E. Metabolic Effects of Dietary Nitrate in Health and Disease. *Cell metabolism* 2018;28(1):9-22. doi: 10.1016/j.cmet.2018.06.007 [published Online First: 2018/07/05]
29. Clifford T, Babateen A, Shannon OM, *et al.* Effects of inorganic nitrate and nitrite consumption on cognitive function and cerebral blood flow: A systematic review and meta-analysis of randomized clinical trials. 2018:1-11. doi: 10.1080/10408398.2018.1453779
30. Babateen AM, Fornelli G, Donini LM, *et al.* Assessment of dietary nitrate intake in humans: a systematic review. *The American journal of clinical nutrition* 2018;108(4):878-88. doi: 10.1093/ajcn/nqy108 [published Online First: 2018/10/16]
31. Hord NG, Tang Y, Bryan NS. Food sources of nitrates and nitrites: the physiologic context for potential health benefits. *The American Journal of Clinical Nutrition* 2009;90(1):1-10. doi: 10.3945/ajcn.2008.27131
32. Siervo M, Lara J, Ogbonmwan I, *et al.* Inorganic Nitrate and Beetroot Juice Supplementation Reduces Blood Pressure in Adults: A Systematic Review and Meta-Analysis. *The Journal of Nutrition* 2013;143(6):818-26. doi: 10.3945/jn.112.170233

For peer review only - http://bmjopen.bmj.com/site/about/guidelines.xhtml

33. Petersen KS, Flock MR, Richter CK, *et al.* Healthy Dietary Patterns for Preventing Cardiometabolic Disease: The Role of Plant-Based Foods and Animal Products. *Current Developments in Nutrition* 2017;1(12) doi: 10.3945/cdn.117.001289
34. Vogt TM, Appel LJ, Obarzanek E, *et al.* Dietary Approaches to Stop Hypertension: rationale, design, and methods. DASH Collaborative Research Group. *Journal of the American Dietetic Association* 1999;99(8 Suppl):S12-8. [published Online First: 1999/08/18]
35. Obarzanek E, Proschan MA, Vollmer WM, *et al.* Individual blood pressure responses to changes in salt intake: results from the DASH-Sodium trial. *Hypertension (Dallas, Tex : 1979)* 2003;42(4):459-67. doi: 10.1161/01.hyp.0000091267.39066.72 [published Online First: 2003/09/04]
36. Farquhar WB, Edwards DG, Jurkovitz CT, *et al.* Dietary sodium and health: more than just blood pressure. *J Am Coll Cardiol* 2015;65(10):1042-50. doi: 10.1016/j.jacc.2014.12.039
37. Mente A, O'Donnell M, Rangarajan S, *et al.* Urinary sodium excretion, blood pressure, cardiovascular disease, and mortality: a community-level prospective epidemiological cohort study. *The Lancet* 2018;392(10146):496-506. doi: 10.1016/S0140-6736(18)31376-X
38. Strazzullo P, D'Elia L, Kandala NB, *et al.* Salt intake, stroke, and cardiovascular disease: meta-analysis of prospective studies. *Bmj* 2009;339:b4567. doi: 10.1136/bmj.b4567 [published Online First: 2009/11/26]
39. He FJ, Li J, MacGregor GA. Effect of longer term modest salt reduction on blood pressure: Cochrane systematic review and meta-analysis of randomised trials. *BMJ : British Medical Journal* 2013;346:f1325. doi: 10.1136/bmj.f1325
40. He FJ, MacGregor GA. Role of salt intake in prevention of cardiovascular disease: controversies and challenges. *Nat Rev Cardiol* 2018;15(6):371-77. doi: 10.1038/s41569-018-0004-1
41. Afshin A, Sur PJ, Fay KA, *et al.* Health effects of dietary risks in 195 countries, 1990–2017: a systematic analysis for the Global Burden of Disease Study 2017. *The Lancet* 2019;393(10184):1958-72. doi: 10.1016/S0140-6736(19)30041-8
42. Trieu K, Neal B, Hawkes C, *et al.* Salt Reduction Initiatives around the World – A Systematic Review of Progress towards the Global Target. *PLOS ONE* 2015;10(7):e0130247. doi: 10.1371/journal.pone.0130247
43. Dong OM. Excessive dietary sodium intake and elevated blood pressure: a review of current prevention and management strategies and the emerging role of pharmaconutrigenetics. *BMJ Nutrition, Prevention & Health* 2018:bmjnph-2018-000004. doi: 10.1136/bmjnph-2018-000004
44. Chan A-W, Tetzlaff JM, Altman DG, *et al.* SPIRIT 2013 statement: defining standard protocol items for clinical trials. *Ann Intern Med* 2013;158(3):200-07. doi: 10.7326/0003-4819-158-3-201302050-00583
45. Chen J, Tian Y, Liao Y, *et al.* Salt-restriction-spoon improved the salt intake among residents in China. *PloS one* 2013;8(11):e78963-e63. doi: 10.1371/journal.pone.0078963
46. Malaysia MoH. Malaysian Dietary Guidelines. In: Malaysia MoH, ed. Malaysia: National Coordinating Committee on Food and Nutrition, 2010:1-261.
47. Sheikh JI, Yesavage JA. Geriatric Depression Scale (GDS): Recent evidence and development of a shorter version. US: Haworth Press, 1986:165-73.
48. Craig CL, Marshall AL, Sjöström M, *et al.* International Physical Activity Questionnaire: 12-Country Reliability and Validity. *Medicine & Science in Sports & Exercise* 2003;35(8):1381-95. doi: 10.1249/01.Mss.0000078924.61453.Fb
49. (IPH) IfPH. National Health and Morbidity Survey 2014: Malaysian Adult Nutrition Survey. Vol. 1: Methodology and General Findings. 2014:1-108.
50. Whitehead AL, Julious SA, Cooper CL, *et al.* Estimating the sample size for a pilot randomised trial to minimise the overall trial sample size for the external pilot and main trial for a continuous outcome variable. *Statistical methods in medical research* 2016;25(3):1057-73. doi: 10.1177/0962280215588241 [published Online First: 2015/06/21]

For peer review only

For peer review only - http://bmjopen.bmj.com/site/about/guidelines.xhtml

For peer review only

For peer review only - http://bmjopen.bmj.com/site/about/guidelines.xhtml

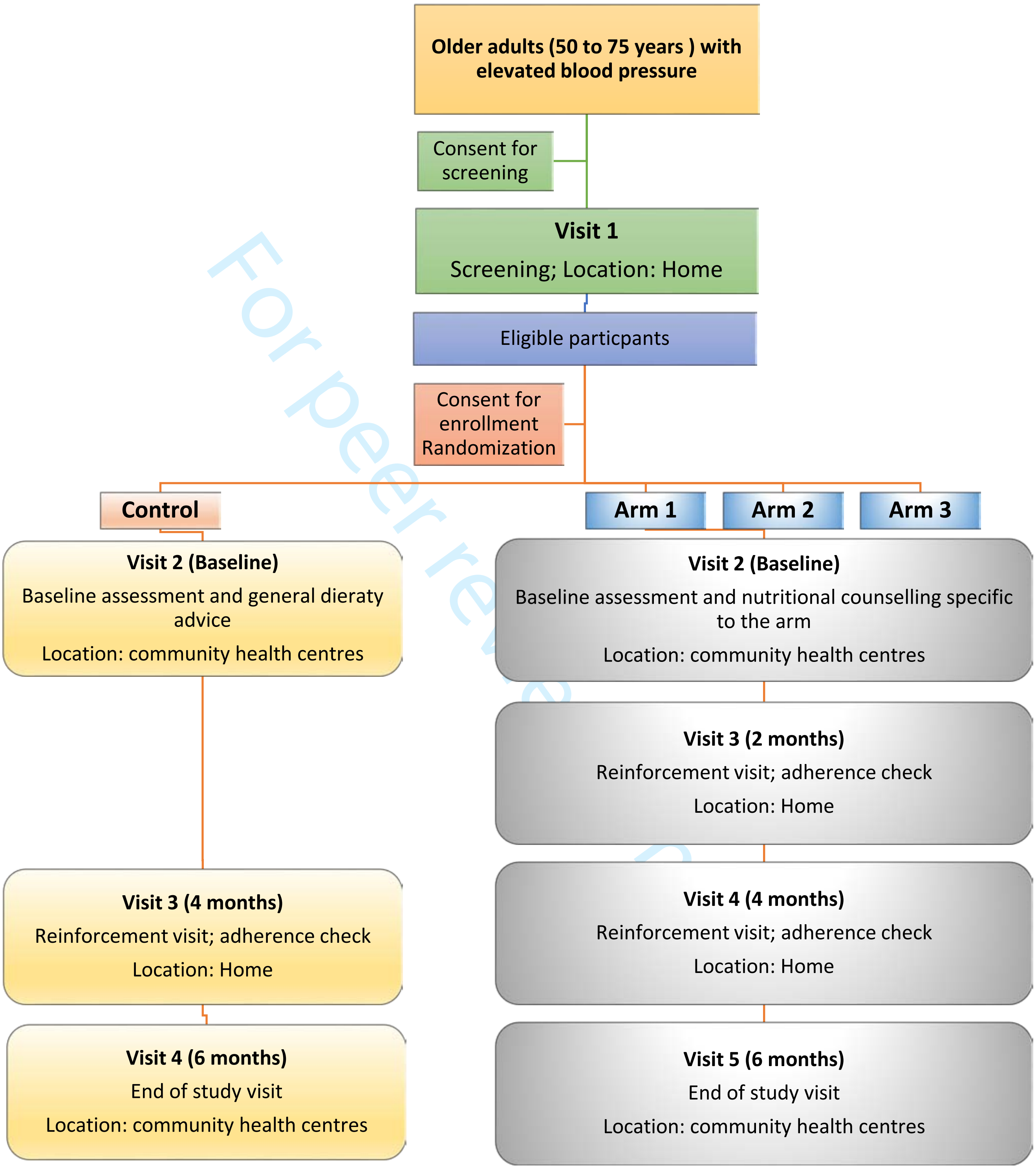

Figure 1: Participant flow through the study

Reporting checklist for protocol of a clinical trial.

Based on the SPIRIT guidelines.

Instructions to authors

Complete this checklist by entering the page numbers from your manuscript where readers will find each of the items listed below.

Your article may not currently address all the items on the checklist. Please modify your text to include the missing information. If you are certain that an item does not apply, please write "n/a" and provide a short explanation.

Upload your completed checklist as an extra file when you submit to a journal.

In your methods section, say that you used the SPIRITreporting guidelines, and cite them as:

Chan A-W, Tetzlaff JM, Altman DG, Laupacis A, Gøtzsche PC, Krleža-Jerić K, Hróbjartsson A, Mann H, Dickersin K, Berlin J, Doré C, Parulekar W, Summerskill W, Groves T, Schulz K, Sox H, Rockhold FW, Rennie D, Moher D. SPIRIT 2013 Statement: Defining standard protocol items for clinical trials. Ann Intern Med. 2013;158(3):200-207

		Reporting Item	Page Number
**Administrative information**			
Title	#1	Descriptive title identifying the study design, population, interventions, and, if applicable, trial acronym	1

For peer review only

Trial registration	#2a	Trial identifier and registry name. If not yet registered, name of intended registry	1
Trial registration: data set	#2b	All items from the World Health Organization Trial Registration Data Set	
Protocol version	#3	Date and version identifier	DePEC protocol v1.0 22/10/2019
Funding	#4	Sources and types of financial, material, and other support	19
Roles and responsibilities: contributorship	#5a	Names, affiliations, and roles of protocol contributors	1 & 19
Roles and responsibilities: sponsor contact information	#5b	Name and contact information for the trial sponsor	19
Roles and responsibilities: sponsor and funder	#5c	Role of study sponsor and funders, if any, in study design; collection, management, analysis, and interpretation of data; writing of the report; and the decision to submit the report for publication, including whether they will have ultimate authority over any of these activities	19

For peer review only

Roles and responsibilities: committees	#5d	Composition, roles, and responsibilities of the coordinating centre, steering committee, endpoint adjudication committee, data management team, and other individuals or groups overseeing the trial, if applicable (see Item 21a for data monitoring committee)	n/a
**Introduction**			
Background and rationale	#6a	Description of research question and justification for undertaking the trial, including summary of relevant studies (published and unpublished) examining benefits and harms for each intervention	4-7
Background and rationale: choice of comparators	#6b	Explanation for choice of comparators	4-7
Objectives	#7	Specific objectives or hypotheses	7
Trial design	#8	Description of trial design including type of trial (eg, parallel group, crossover, factorial, single group), allocation ratio, and framework (eg, superiority, equivalence, non-inferiority, exploratory)	11
**Methods: Participants, interventions, and outcomes**			

For peer review only

Study setting	#9	Description of study settings (eg, community clinic, academic hospital) and list of countries where data will be collected. Reference to where list of study sites can be obtained	8
Eligibility criteria	#10	Inclusion and exclusion criteria for participants. If applicable, eligibility criteria for study centres and individuals who will perform the interventions (eg, surgeons, psychotherapists)	8-9
Interventions: description	#11a	Interventions for each group with sufficient detail to allow replication, including how and when they will be administered	11
Interventions: modifications	#11b	Criteria for discontinuing or modifying allocated interventions for a given trial participant (eg, drug dose change in response to harms, participant request, or improving / worsening disease)	n/a
Interventions: adherance	#11c	Strategies to improve adherence to intervention protocols, and any procedures for monitoring adherence (eg, drug tablet return; laboratory tests)	n/a
Interventions: concomitant care	#11d	Relevant concomitant care and interventions that are permitted or prohibited during the trial	n/a
Outcomes	#12	Primary, secondary, and other outcomes, including the specific measurement variable (eg, systolic blood pressure), analysis metric (eg, change from baseline,	11-16

For peer review only

		final value, time to event), method of aggregation (eg, median, proportion), and time point for each outcome. Explanation of the clinical relevance of chosen efficacy and harm outcomes is strongly recommended	
Participant timeline	#13	Time schedule of enrolment, interventions (including any run-ins and washouts), assessments, and visits for participants. A schematic diagram is highly recommended (see Figure)	10 & 12
Sample size	#14	Estimated number of participants needed to achieve study objectives and how it was determined, including clinical and statistical assumptions supporting any sample size calculations	16
Recruitment	#15	Strategies for achieving adequate participant enrolment to reach target sample size	8
**Methods: Assignment of interventions (for controlled trials)**			
Allocation: sequence generation	#16a	Method of generating the allocation sequence (eg, computer-generated random numbers), and list of any factors for stratification. To reduce predictability of a random sequence, details of any planned restriction (eg, blocking) should be provided in a	11

For peer review only

		separate document that is unavailable to those who enrol participants or assign interventions	
Allocation concealment mechanism	#16b	Mechanism of implementing the allocation sequence (eg, central telephone; sequentially numbered, opaque, sealed envelopes), describing any steps to conceal the sequence until interventions are assigned	11
Allocation: implementation	#16c	Who will generate the allocation sequence, who will enrol participants, and who will assign participants to interventions	11
Blinding (masking)	#17a	Who will be blinded after assignment to interventions (eg, trial participants, care providers, outcome assessors, data analysts), and how	11
Blinding (masking): emergency unblinding	#17b	If blinded, circumstances under which unblinding is permissible, and procedure for revealing a participant's allocated intervention during the trial	11
**Methods: Data collection, management, and analysis**			
Data collection plan	#18a	Plans for assessment and collection of outcome, baseline, and other trial data, including any related processes to promote data quality (eg, duplicate measurements, training of assessors) and a	17

For peer review only

		description of study instruments (eg, questionnaires, laboratory tests) along with their reliability and validity, if known. Reference to where data collection forms can be found, if not in the protocol	
Data collection plan: retention	#18b	Plans to promote participant retention and complete follow-up, including list of any outcome data to be collected for participants who discontinue or deviate from intervention protocols	17
Data management	#19	Plans for data entry, coding, security, and storage, including any related processes to promote data quality (eg, double data entry; range checks for data values). Reference to where details of data management procedures can be found, if not in the protocol	17
Statistics: outcomes	#20a	Statistical methods for analysing primary and secondary outcomes. Reference to where other details of the statistical analysis plan can be found, if not in the protocol	17
Statistics: additional analyses	#20b	Methods for any additional analyses (eg, subgroup and adjusted analyses)	17
Statistics: analysis population and missing data	#20c	Definition of analysis population relating to protocol non-adherence (eg, as randomised analysis), and any statistical methods to handle missing data (eg, multiple imputation)	17

For peer review only

Methods: Monitoring

Data monitoring: formal committee	#21a	Composition of data monitoring committee (DMC); summary of its role and reporting structure; statement of whether it is independent from the sponsor and competing interests; and reference to where further details about its charter can be found, if not in the protocol. Alternatively, an explanation of why a DMC is not needed	n/a
Data monitoring: interim analysis	#21b	Description of any interim analyses and stopping guidelines, including who will have access to these interim results and make the final decision to terminate the trial	n/a
Harms	#22	Plans for collecting, assessing, reporting, and managing solicited and spontaneously reported adverse events and other unintended effects of trial interventions or trial conduct	17
Auditing	#23	Frequency and procedures for auditing trial conduct, if any, and whether the process will be independent from investigators and the sponsor	17

Ethics and dissemination

Research ethics approval	#24	Plans for seeking research ethics committee / institutional review board (REC / IRB) approval	18

For peer review only

Protocol amendments	#25	Plans for communicating important protocol modifications (eg, changes to eligibility criteria, outcomes, analyses) to relevant parties (eg, investigators, REC / IRBs, trial participants, trial registries, journals, regulators)	n/a
Consent or assent	#26a	Who will obtain informed consent or assent from potential trial participants or authorised surrogates, and how (see Item 32)	8
Consent or assent: ancillary studies	#26b	Additional consent provisions for collection and use of participant data and biological specimens in ancillary studies, if applicable	n/a
Confidentiality	#27	How personal information about potential and enrolled participants will be collected, shared, and maintained in order to protect confidentiality before, during, and after the trial	18
Declaration of interests	#28	Financial and other competing interests for principal investigators for the overall trial and each study site	1
Data access	#29	Statement of who will have access to the final trial dataset, and disclosure of contractual agreements that limit such access for investigators	17-18
Ancillary and post trial care	#30	Provisions, if any, for ancillary and post-trial care, and for compensation to those who suffer harm from trial participation	17-18

For peer review only

Dissemination policy: trial results	#31a	Plans for investigators and sponsor to communicate trial results to participants, healthcare professionals, the public, and other relevant groups (eg, via publication, reporting in results databases, or other data sharing arrangements), including any publication restrictions	17-18
Dissemination policy: authorship	#31b	Authorship eligibility guidelines and any intended use of professional writers	n/a
Dissemination policy: reproducible research	#31c	Plans, if any, for granting public access to the full protocol, participant-level dataset, and statistical code	n/a
**Appendices**			
Informed consent materials	#32	Model consent form and other related documentation given to participants and authorised surrogates	n/a
Biological specimens	#33	Plans for collection, laboratory evaluation, and storage of biological specimens for genetic or molecular analysis in the current trial and for future use in ancillary studies, if applicable	n/a

None The SPIRIT checklist is distributed under the terms of the Creative Commons Attribution License CC-BY-ND 3.0. This checklist can be completed online using https://www.goodreports.org/, a tool made by the EQUATOR Network in collaboration with Penelope.ai

For peer review only

BMJ Open

The feasibility and acceptability of a dietary intervention study to reduce salt intake and increase high-nitrate vegetable consumption among middle-aged and older Malaysian adults with elevated blood pressure: a study protocol

Journal:	*BMJ Open*
Manuscript ID	bmjopen-2019-035453.R1
Article Type:	Protocol
Date Submitted by the Author:	21-Apr-2020
Complete List of Authors:	McGrattan, Andrea; Newcastle University, Population Health Sciences Institute<br>Mohan, Devi; Monash University - Malaysia Campus, Global Public Health, School of Medicine<br>Chua, Pei Wei; Monash University - Malaysia Campus, Global Public Health. Jeffrey Cheah School of Medicine and Health Sciences; South East Asia Community Observatory (SEACO)<br>Mat Hussin, Azizah; Universiti Kuala Lumpur, Kampus Cawangan Institute of Medical Science Technology<br>Soh, Yee Chang; Monash University - Malaysia Campus, Global Public Health; Jeffrey Cheah School of Medicine; South East Asia Community Observatory (SEACO)<br>Alawad, Mawada; Monash University - Malaysia Campus, Global Public Health. Jeffrey Cheah School of Medicine and Health Sciences; South East Asia Community Observatory (SEACO)<br>bin Kassim, Zaid; District Health Office (PKD) Segamat<br>bin Mohd Ghazali, Ahmad Nizal; District Health Office (PKD) Segamat<br>Stephan, Blossom; University of Nottingham, School of Medicine<br>Allotey, Pascale; Monash University - Malaysia Campus, Global Public Health. Jeffrey Cheah School of Medicine and Health Sciences; United Nations University International Institute for Global Health<br>Reidpath, Daniel; Monash University - Malaysia Campus, Global Public Health. Jeffrey Cheah School of Medicine and Health Sciences; South East Asia Community Observatory (SEACO)<br>Robinson, Louise; Newcastle University, Population Health Sciences Institute<br>Siervo, Mario; University of Nottingham, School of Life Sciences
**Primary Subject Heading**:	Public health
Secondary Subject Heading:	Nutrition and metabolism, Global health, Research methods
Keywords:	Dementia < NEUROLOGY, NUTRITION & DIETETICS, PUBLIC HEALTH

SCHOLARONE™
Manuscripts

I, the Submitting Author has the right to grant and does grant on behalf of all authors of the Work (as defined in the below author licence), an exclusive licence and/or a non-exclusive licence for contributions from authors who are: i) UK Crown employees; ii) where BMJ has agreed a CC-BY licence shall apply, and/or iii) in accordance with the terms applicable for US Federal Government officers or employees acting as part of their official duties; on a worldwide, perpetual, irrevocable, royalty-free basis to BMJ Publishing Group Ltd ("BMJ") its licensees and where the relevant Journal is co-owned by BMJ to the co-owners of the Journal, to publish the Work in this journal and any other BMJ products and to exploit all rights, as set out in our licence.

The Submitting Author accepts and understands that any supply made under these terms is made by BMJ to the Submitting Author unless you are acting as an employee on behalf of your employer or a postgraduate student of an affiliated institution which is paying any applicable article publishing charge ("APC") for Open Access articles. Where the Submitting Author wishes to make the Work available on an Open Access basis (and intends to pay the relevant APC), the terms of reuse of such Open Access shall be governed by a Creative Commons licence – details of these licences and which Creative Commons licence will apply to this Work are set out in our licence referred to above.

Other than as permitted in any relevant BMJ Author's Self Archiving Policies, I confirm this Work has not been accepted for publication elsewhere, is not being considered for publication elsewhere and does not duplicate material already published. I confirm all authors consent to publication of this Work and authorise the granting of this licence.

For peer review only

DePEC protocol R1. 10/04/2020

TITLE

The feasibility and acceptability of a dietary intervention study to reduce salt intake and increase high-nitrate vegetable consumption among middle-aged and older Malaysian adults with elevated blood pressure: a study protocol

AUTHORS

Andrea M McGrattan[1*], Devi Mohan[2], Chua Pei Wei[2,7], Azizah Mat Hussin[3], Yee Chang Soh[2,7], Mawada Azhari Sidahmed Alawad[2,7], Zaid Bin Kassim[4], Ahmad Nizal Bin Mohd Ghazali[4], Blossom C M Stephan[5], Pascale Allotey[2,6], Daniel D Reidpath[2,7], Louise Robinson[1], Mario Siervo[8]

AFFILIATIONS

[1]Population Health Sciences Institute, Newcastle University, Newcastle, United Kingdom.

[2]Global Public Health. Jeffrey Cheah School of Medicine and Health Sciences, Monash University Malaysia, Selangor, Malaysia.

[3]Universiti Kuala Lumpur, Kampus Cawangan Institute of Medical Science Technology, Kuala Lumpur, Malaysia.

[4]District Health Office (PKD) Segamat, Jalan Gudang Ubat 85000, Segamat Johor, Malaysia

[5] School of Medicine, The University of Nottingham, United Kingdom.

[6] Director of the United Nations University International Institute for Global Health (UNU-IIGH), Jalan Yaacob Latiff, 56000 Cheras, Kuala Lumpur, Malaysia.

[7] South East Asia Community Observatory (SEACO), Segamat, Johor, Malaysia.

[8] School of Life Sciences, The University of Nottingham Medical School, Nottingham, United Kingdom.

*Corresponding author: Dr Andrea McGrattan; Population Health Sciences Institute; Newcastle University, Newcastle, United Kingdom; andrea.mcgrattan@newcastle.ac.uk ; +44 (0) 191 208 5467

The material presented in this manuscript is original and it has not been submitted for publication elsewhere while under consideration by BMJ Open

Abstract Word Count: 305

Main Text Word Count: 5,827

References: 48

Tables: 2

Figures: 1

Online supplementary material: 0

For peer review only - http://bmjopen.bmj.com/site/about/guidelines.xhtml

ABSTRACT

Introduction

Global population ageing is one the key factors linked to the projected rise of dementia incidence. Hence, there is a clear need to identify strategies to overcome this expected health burden and have a meaningful impact on populations' health worldwide. Current evidence supports the role of modifiable dietary and lifestyle risk factors in reducing the risk of dementia. In South-East Asia, changes in individual eating and lifestyle patterns under the influence of westernised habits, have resulted in significant increases in the prevalence of metabolic, cardiovascular and neurodegenerative non-communicable diseases (NCDs). Low vegetable consumption and high sodium intake have been identified as key contributors to the increased prevalence of NCDs in these countries. Therefore, nutritional and lifestyle strategies targeting these dietary risk factors are warranted in these populations to prevent cardio-metabolic and neurodegenerative chronic diseases.

Methods and Analysis

The primary aim of this randomised feasibility trial is to demonstrate the acceptability of a dietary intervention to increase the consumption of high-nitrate green leafy vegetables and reduce salt intake over a 6-month period among Malaysian adults with raised blood pressure. Secondary outcomes will include blood pressure, cognitive function, body composition and physical function (including muscle strength and gait speed). Adherence to the dietary intervention will be assessed through collection of biological samples, 24 hour recall and food frequency questionnaire. A subgroup of participants will also complete post-intervention focus groups to further explore the feasibility considerations of executing a larger trial, the ability of these individuals to make dietary changes and the barriers and facilitators associated with implementing these changes.

Ethics and Dissemination

Ethical approval has been obtained from MUHREC (Monash University Human Research Ethics Committee) and MREC (Medical Research and Ethics Committee) of Malaysia. Results of the study will be disseminated via peer-reviewed publications and presentations at national and international conferences. Trial Registration Number: ISRCTN47562685

For peer review only - http://bmjopen.bmj.com/site/about/guidelines.xhtml

For peer review only

ARTICLE SUMMARY

Strengths and limitations of the study

1. Feasibility of the first 2*2 trial testing single and combined effects of dietary nitrate and salt reduction for the prevention of cognitive decline in developing countries
2. Longest high-nitrate based intervention ever conducted
3. Use of objective biomarkers for the assessment of adherence to the dietary intervention and internal validation of the dietary assessment methods used (Urinary sodium – 24 hr urine *vs* spot urine / Nitrate levels in blood - dried blood spots *vs* plasma, and ELISA *vs* gold standard / Nitrate in saliva – salivary strip (point of care test) *vs* ELISA using saliva samples)
4. Mixed-method approach to obtain key information on the feasibility of the dietary intervention
5. Owing to the small sample size and the feasibility nature of this study, the planned comparative analysis is only exploratory, and therefore, the efficacy of the intervention between groups cannot be determined. However, the results of this study will be instrumental in the design and calculation of the sample size of a larger, follow-on efficacy trial

INTRODUCTION

Dementia is an incurable progressive syndrome characterised by multiple cognitive deficits and loss of independence, and its pathogenesis is linked to multiple distinct neuropathological processes including Alzheimer's disease (AD) and/or vascular pathology.[1] Observational evidence suggests that several of the pathological processes underlying dementia might be delayed or prevented by interventions focussed on dietary and behavioural changes[2]. Hence, in the absence of definite pharmacological treatments, these options represent a key strategy to alleviate its individual and social impact.[3] Optimal control of modifiable cardiovascular factors such as blood pressure, as well as adherence to lifestyle recommendations such as a healthy diet and physical activity, have been consistently associated with reduced dementia risk in observational studies.[2-4] The role of nutrition for the prevention of cognitive decline in cognitively healthy individuals and populations with cognitive impairment has been extensively investigated by testing the role that single nutrients, such as n-3 PUFA or B vitamins[5] as well as whole dietary patterns, such as the DASH diet[6] and the Mediterranean diet[7 8]. There are a small number of RCTs available to support the effect of diet on cognitive function in Mild Cognitive Impairment[9] and among those at risk of cognitive decline or cognitively healthy populations[10]; however the trials have a small sample size and overall modest quality.

Malaysia is one of many Asian countries experiencing rapid socio-economic and nutritional transitions[11]. Changes in individual eating and lifestyle patterns under the influence of westernised

For peer review only - http://bmjopen.bmj.com/site/about/guidelines.xhtml

habits, as well as a rise in food availability and financial prosperity of the population, have resulted in significant increases in the prevalence of chronic metabolic, cardiovascular and neurodegenerative non-communicable diseases [NCD][12]. Implementation of effective public health interventions for the prevention of dementia is challenging in countries with limited social and health care resources which are unable to meet the growing demands of ageing populations. In a systematic review of 22 randomised controlled trials, the effect of nutritional interventions on cognitive performance in developing economies of East Asia was investigated. The majority of the studies showed some significant beneficial effects of the nutritional interventions on cognitive performance. However, these effects were not consistent across all of the neuropsychological tests used in each study, and, as a result, significant beneficial effects for a specific intervention were often not seen in the meta-analysis. [10] Therefore, there is a need for further well-designed nutritional intervention studies to test the efficacy of interventions for the early prevention of cognitive decline and dementia.

Recent analyses have identified inorganic nitrate as a potential nutrient involved the improvement of vascular and metabolic health.[13] Beetroot and green leafy vegetables, such as spinach, lettuce and rocket, are a key source of naturally available inorganic nitrate, containing over 250 mg of nitrate per 100 g of fresh vegetable weight.[14] Inorganic nitrate is closely linked to the metabolism of Nitric Oxide (NO) which is known for its multiple effects on physiological function such as control of blood pressure, brain function and immunity.[15] An increase in inorganic nitrate consumption has been associated with an increase in NO production via complex mechanisms involving oral microbiota, gastric environment and activation of enzymes[16]. Recent evidence from epidemiological studies and clinical trials have demonstrated the association of increased nitrate intake with improved blood pressure, metabolic health and cognitive function.[17 18] A 15-year prospective cohort study investigated the association of consumption of high nitrate vegetables and risk of atherosclerotic vascular disease (ASVD) mortality. Results showed that a consumption of approximately 30 mg/day higher nitrate intake from vegetables was associated with a 21% lower risk of ASVD mortality. This nitrate amount equates to approximately 10–30 g/day of nitrate-rich vegetables.[19] Furthermore, a systematic review and meta-analysis demonstrated that inorganic nitrate and beetroot juice supplementation were associated with a significant decrease in blood pressure. The pooled effect for both interventions showed a reduction in systolic blood pressure of 4.4 mmHg, with a more modest decrease of 1.1 mmHg in diastolic blood pressure.[20] However, the impact on cognition is still unknown with studies of short intervention durations and among small samples, and limited studies conducted in developing countries.[14 17] Nevertheless, with hypertension highlighted as an important modifiable risk factor for dementia,[3 21]

For peer review only - http://bmjopen.bmj.com/site/about/guidelines.xhtml

more studies that test the implementation of dietary interventions that aim to improve blood pressure are warranted.

Sodium is the main electrolyte contained in extracellular fluids and involved in regulation of blood pressure and cellular activities. However, an excess sodium intake has been linked to extracellular volume expansion resulting in increased blood pressure and renal sodium excretion.[22] The DASH diet has been specifically developed to promote consumption of healthy dietary choices alongside strictly controlled sodium intake.[23] Secondary analyses derived from seminal DASH studies showed additional benefits on blood pressure and cardio-metabolic health in individuals with lower salt intake and adherence to a healthy dietary pattern[24]. These analyses have been extended to cognitive function with promising results,[25 26] but the evidence has mostly come from studies conducted in the United States and no studies to our knowledge have been conducted in Low and Middle Income Countries (LMICs). Epidemiological studies have repeatedly proven the association of high salt diets with impaired blood pressure control and increased cardiovascular risk;[27 28] this evidence has been confirmed in several clinical trials testing the effects of salt-reducing interventions and demonstrating the protective effects of salt reduction on cardiovascular health.[29 30] A prospective study looked at the relationship between a reduction in salt intake with blood pressure, mortality from stroke and ischaemic heart disease (IHD). [31] A decrease in salt intake by 1.4 g/day, as measured by 24 hour urinary sodium, contributed to a reduction in stroke and IHD mortality and in addition, among individuals who were not on antihypertensive medication, there was significant a fall in systolic blood pressure of 2.7±0.34mmHg.[31] Excessive sodium consumption is recognised as a global health issue[32] with efforts aiming to reduce salt intake at the population level by 30% by 2025. Seventy-five countries have already implemented national sodium reduction programmes to help meet this target.[33] The strategies to reduce sodium intake at the population level have used a variety of approaches, including working with industry to reformulate food products, establishing sodium content targets for foods, revising food labelling schemes, taxation of high-salt foods and development of educational interventions.[34]

In sum, an increased consumption of dietary nitrate and lower salt intake may have additional benefits on health outcomes such as blood pressure and cognition, mediated by an increase in NO generation (i.e., nitrate) and control of vascular tone and cellular metabolism (i.e., sodium). However, this hypothesis has never been tested in previous interventions which represents the main aim of the feasibility study proposed in this protocol. The Global Health Dementia Prevention and Enhanced Care (DePEC) project is a National Institute for Health Research (NIHR) funded research study with a key

For peer review only - http://bmjopen.bmj.com/site/about/guidelines.xhtml

goal to reduce the personal and societal burden of dementia, by developing approaches to reduce future numbers of people developing dementia and develop more efficient post-diagnostic care. The project brings together: dementia experts in public health/primary care (Newcastle University), global epidemiological 10/66 dementia research group from King's College London (KCL), with a strong existing LMICs network and new LMICs partners, one from each economic category (upper LMIC-Malaysia, LMIC-India and least developed Tanzania). The DePEC-Nutrition feasibility study (presented here) is one of five work streams within the overall DePEC project.

Purpose and Aims

The overall objective of the DePEC-Nutrition feasibility study is to demonstrate the acceptability of a dietary intervention to increase the consumption in high-nitrate green leafy vegetables and reduce salt intake over a 6-month period among Malaysian adults with raised blood pressure. The objectives focus on the core areas of a trial that need to work for the study to succeed procedurally, such as recruitment ability and participant retention, data collection procedures and assessment methods used, potential to deliver the dietary intervention and resource requirement. Information on the effect size of the intervention on cognition and blood pressure will also be determined, which will be instrumental in the design and calculation of the sample size of a follow-on efficacy trial.

METHODS AND ANALYSIS

Study design

The DePEC-Nutrition feasibility study is a 6 month randomised 2*2 factorial trial including four parallel arms: (1) control, (2) high-nitrate vegetable consumption, (3) low salt consumption and (4) combined high-nitrate vegetable consumption plus low salt consumption. The trial will be conducted among 120 middle-aged and older Malaysian adults. Sponsor and grant number: GHR Group: 16/137/62 – NIHR Global Health Research Group on Dementia Prevention and Enhanced Care (DePEC), Newcastle University, United Kingdom. This trial has been registered with ISRCTN (ISRCTN47562685). The SPIRIT reporting guidelines were used to guide the preparation of this protocol.[35]

Participants

This study is embedded within the South East Asia Community Observatory (SEACO) population (http://www.seaco.asia/). SEACO is a health and demographic surveillance site (HDSS); a unique research platform in population health and wellbeing focused on a middle-income community in Segamat, Johor in the southern tip of the Peninsular Malaysia. The district of Segamat comprises urban, rural and plantation areas with an ethnic mix including Malays, Chinese and Indian, in similar

For peer review only - http://bmjopen.bmj.com/site/about/guidelines.xhtml

proportions to the national population. SEACO runs its HDSS research platform in five sub-districts (mukim) of the Segamat district: Bekok, Chaah, Gemereh, Jabi and Sungai Segamat. The SEACO population was established using a baseline census (March 2012 to February 2013) that included information on sociodemographic status and self-reported health conditions. Consenting participants underwent detailed health profile assessments in the first health round in 2013-2014. The next census was done in 2017, with a total number of 40,015 participants completing the assessment. Individuals were re-seen after one year (in 2018) for the second health round. Individuals aged 50 years and over, who are living in Segamat, Johor and have been assessed as part of SEACO during the health round in 2018 will form the study population for the DePEC-Nutrition feasibility study. The baseline assessment will be carried out in the Klink Kesihatans (KK) in Sungai Segamat sub–district (a community health clinic in Segamat, Johor). Hence, only participants from within 5 km radius of KK Segamat will be included in the study population. A total of 2433 participants have been identified on the database as potentially eligible for this study by broad application of the study criteria (mean age (standard deviation) - 61.27 (6.77) years and number of males – n=1078 (44.3%). This subset of particiapnts will be screened in detail, and then will be informed of the study and invited for a screening visit as outlined in the study procedures.

Recruitment

The community will be informed about this study via a local Community Engagement Committee (CEC) who meet regularly to distribute information on projects planned within SEACO. Participants will be randomly identified by the application of the inclusion and exclusion criteria to the SEACO health surveillance database. Potential participants will be approached via a home visit and will be provided with information about the study. They will be given an opportunity to read detailed information about the study procedures and can ask questions. Thereafter, their interest of participating in the study will be evaluated. If they agree to take part, a screening assessment will be completed. If eligible, participants will be invited to attend a baseline study visit at the health facilities within the community.

Eligibility criteria

To be eligible for inclusion in this study, participants must report as:

1. Male and female participants with an age between 50 and 75 years from the SEACO database
2. Pre-hypertensive, stage 1 hypertensive or diagnosed hypertensive: Any person with a self-reported history of hypertension (on or not on medication) or with elevated blood pressure (SBP 120-159 mmHg or DBP 80-99 mmHg)

For peer review only - http://bmjopen.bmj.com/site/about/guidelines.xhtml

3. Not severely cognitively impaired (defined as a Mini Mental Examination Status [MMSE] score of 19 or over)

Participants will be excluded from the study if they:

1. Are participating in other research clinical studies
2. Are unable to provide consent
3. Have systolic blood pressure more than or equal to 160 mmHg and / or diastolic blood pressure more than or equal to 100 mmHg
4. Adhere to any therapeutic diet such as weight loss treatments or a gluten-free diet
5. Are vegan (these individuals are likely to have a high intake of vegetables). Since Indian participants are more likely to follow a vegetarian or semi-vegetarian diet, in order to maintain representativeness across ethnic groups included in the study, only participants who are vegan will be excluded from the study
6. Participants with a body mass index (BMI) less than 18.5 kg/m^2
7. Have a history of active cancer or any diagnosis of cancer in the last 5 years
8. Have a history of excessive alcohol intake (>21 units of alcohol per week)
9. Have a history of brain damage or significant head trauma (resulting in a loss of consciousness)
10. Have a diagnosis of acute and chronic medical conditions interfering with the study outcomes such as systemic infections (tuberculosis, hepatitis B, HIV/AIDS), severe liver and kidney diseases, inflammatory bowel diseases, coronary heart diseases, cerebrovascular diseases or is a diabetic on insulin therapy
11. Have had any major surgical procedures (in the past six weeks or planned in the next year) that could interfere with the study outcomes
12. Have current diagnosis of moderate or severe depression, or other serious mental or brain disorder and / or are currently on psychiatric medication (antidepressants, sedatives, antipsychotics)
13. Regularly uses sodium-altering drugs (angiotensin-converting-enzyme (ACE) inhibitors, angiotensin receptor blockers (ARB), corticosteroids, diuretics, hormonal therapies (oestrogens, thyroxin, and progesterone), non-steroidal anti-inflammatory drugs (NSAIDs), proton-pump inhibitors), organic nitrates
14. Has changed in anti-hypertensive medication regimen for the previous 3 months
15. Have limited mobility due to any reason
16. Are planning to move house within a year

For peer review only - http://bmjopen.bmj.com/site/about/guidelines.xhtml

Study procedure

Data collection will be undertaken directly on an electronic hand held device (Samsung Galaxy Tab 3v – Survey CTO). Questionnaire items will be read out-loud by the data collector and participant responses will be recorded directly onto the electronic tablet. The flow of participants through the DePEC-Nutrition feasibility study is illustrated in Figure 1. At the screening visit, written informed consent will be first obtained, after which participants will complete a screening questionnaire to collect information on demographics, co-morbidities, medication history, cognitive function (MMSE), body weight, height and resting blood pressure. If eligible, participants will undergo baseline assessments, consisting of two parts; a home visit (part 1), followed by an appointment at KK, Segamat (part 2). Baseline assessments will include: obtaining respondent's signed informed consent for study participation, eligibility assessment (medical and medication history), dietary assessment (24-hour recall and food frequency questionnaire (FFQ)), cognitive assessment, collection of biological samples (blood, dried blood spots, 24-urine and spot urine samples, saliva samples, and salivary strips), measurements of hand grip and gait speed, physical activity (IPAQ) and depression (GDS).

An interim two-month visit will take place for those in the intervention groups only, to provide reinforcement messages relating to the allocated dietary intervention and with a view to enhancing compliance with the intervention. In addition, a brief adherence questionnaire will be administered, body weight and resting blood pressure will be measured and salivary strip and spot urine samples will be collected. The same measurements and further dietary reinforcement messages will be conducted at an interim four-month visit for the participants allocated to the intervention arms. Participants in the control arm will only have one interim visit at month four and will have their weight and blood pressure measured. The end of study visit (at KK, Segamat) will include the conduction of the measurements collected at baseline, including cognitive assessment, dietary assessment, and collection of biological samples (blood, dried blood spots, 24-urine and spot urine samples, saliva samples, salivary strips). In addition, an exit group session (focus group evaluation) and a self-administered feedback questionnaire will be scheduled to evaluate the adherence to the interventions and obtain detailed feedback on the overall study protocol. Finally, the participants will be offered a token of appreciation to acknowledge their participation in the study and to compensate for travel expenses. This is usually to the value of RM 25.

Randomisation and blinding

The SEACO health round database will serve as the sampling frame, from which eligible participants are identified. Block randomisation will be carried out to assign eligible participants into

For peer review only - http://bmjopen.bmj.com/site/about/guidelines.xhtml

one of the four arms. Block sizes of four participants will be used in the randomisation and will be generated by a member of the research team not involved in the data collection. The interviewers will be blinded to the participant assignment to intervention arms. Participant blinding to the intervention allocation will not be possible due to the nature of the dietary intervention study.

Intervention and control arms

This dietary and behavioural intervention will focus on key components that are related to changing local dietary patterns influenced by nutritional transition trends in Malaysia. It aims to target two key components that have protective (dietary nitrate) and negative (salt intake) effects on cardiovascular and cognitive health. The intervention will target individuals with a higher CVD risk to adopt a tailored strategy to enhance daily consumption of green leafy vegetables and / or reduce salt intake. Nutritional counselling sessions with a medical doctor will be conducted at baseline to provide sustainable and effective strategies to improve dietary habits as well as monitor and address key barriers to dietary change. These sessions will be delivered in a group setting using PowerPoint slides and practical activities to educate the group on the allocated dietary intervention. All intervention groups will receive written information focussed on health benefits, sources, recommended intakes and practical tips related to one of the following dietary interventions; (1) to increase dietary nitrate (2) to reduce salt intake (3) to increase dietary nitrate and to reduce salt intake. Those who are randomised to the salt intervention (either alone or combined) will receive a measuring spoon as an additional strategy to support individuals to understand portion size and measure salt intake, as successfully tested by an intervention study in China.[36] Furthermore, the Ministry for Health Malaysia set out in their Salt Reduction Strategy 2015-2020 how reducing the amount of salt and sauces consumed in the home is a key priority.[37] This provides justification for inclusion of the salt restriction spoon as an intervention strategy. The intervention groups will also receive bi-weekly text message reminders to provide reinforcement of the key dietary behaviour change messages relevant for their group allocation. Compliance will be monitored during the intermediate visits (2 and 4 months, respectively) where participants will also receive reinforcement video messages to remind participants of the key dietary advice that was discussed during the counselling sessions at baseline. Those allocated to the control group (4) will receive written information on general healthy eating and lifestyle advice based on the Malaysian dietary guidelines.[38]

Outcome measures

Outcome assessments will be conducted at baseline, month four and month six for all groups, with an additional assessment visit at month two for those in the intervention groups. Assessments at baseline

For peer review only - http://bmjopen.bmj.com/site/about/guidelines.xhtml

and month 6 will be conducted in clinic / at home, with remaining assessments completed in the participants own home. The same instruments will be used for measurements at each time point. Assessors are trained prior to the assessments to follow a standardised protocol. A timeline of the study procedures can be found in Table 1.

For peer review only

For peer review only - http://bmjopen.bmj.com/site/about/guidelines.xhtml

Table 1: Timeline of study procedures

**Procedures**	**Consent/Screening**	**Baseline Week 1**	**Week 2**	**Week 4**	**Week 6**	**Week 8**	**Week 10**	**Week 12**	**Week 16**	**Week 18**	**Week 20**	**Week 22**	**End of study Week 24**
**Inclusion/Exclusion**	✓												
**Consent**	✓												
**Activities for intervention group**													
Dietary education		✓											
Reinforcement at home visit		✓				✓			✓				
Group education counselling session		✓											
Home visit						✓			✓				
SMS messages			✓	✓	✓	✓	✓	✓	✓	✓	✓	✓	✓
**Activities for control group**													
General health promotion message		✓											
Home visit									✓				
**Study Measurements**													
Sociodemographic		✓											
Concomitant medication check list		✓				✓							✓
Any medical events		✓				✓							✓
Physical activity (IPAQ)		✓											✓
Depression (GDS)		✓											✓
Food frequency questionnaire (FFQ)		✓											✓
24hr dietary recall		✓											✓
Nitrate: Urine		✓**a**				✓***b**			✓**b**				✓**a**
Salt: Urine		✓**a**				✓***b**			✓**b**				✓**a**
Cognitive performance		✓											✓
Resting blood pressure		✓				✓*			✓				✓
Body composition		✓				✓***c**			✓**c**				✓
Physical function		✓											✓
Blood Sample and dried blood spot		✓											✓
Saliva sample and salivary strips		✓				✓*‡			✓‡				✓

a: 24-urine and spot urine samples; b: spot urine sample only; c: body weight only; * intervention groups only; ‡ saliva strips only

Primary outcome

The primary outcome measures are related to feasibility and will be collected via questionnaire, review of the study procedures and by review of data collector field notes. Patient feedback, acceptance and adherence issues will be additionally assessed through qualitative focus groups conducted post-intervention. The specific feasibility considerations that will be assessed in the study have been outlined in Table 2. The CONSORT 2010 statement[39] will be used to guide the reporting of the feasibility results.

Table 2: Primary outcome: Feasibility considerations to be assessed

**Feasibility Considerations**	
**Recruitment**	1. Recruitment rates<br>2. Time taken for the recruitment of participants<br>3. Characteristics of recruited sample as against expected<br>4. Non-response rates<br>5. Reasons for non-response
**Retention and Completion**	1. Retention rates<br>2. Number of drops and reasons for withdrawal<br>3. Follow-up response rates (2 months and 4 months follow-ups)
**Data Collection methods**<br>Feasibility of measurement tools	1. Time taken to fill in questionnaires<br>2. Missing data from questionnaires<br>3. Understanding level of procedures and measurement protocols<br>4. Suitability of outcome measures<br>5. Sensitivity of outcome measures to change<br>6. Internal consistency of outcome measures - validation of data collection methods (24 hours vs spot urine; saliva samples vs salivary strips)
Resource Capacity	1. Data collection and measurement equipment<br>2. Field support (transportation, IT, etc.)<br>3. Personnel (were the right numbers of people with the right skills available when they were needed)<br>4. Duration of each phase
**Acceptability and adherence to intervention**	1. Barriers to participation (collected at screening interview)<br>2. Burden (reasons for not taking part/discontinuation or dropping out)<br>3. Qualitative enquiry/feedback from participants and research staff<br>4. Focus group evaluation - This part of the feasibility trial will separately examine the acceptability and suitability of: intervention materials; intervention timing; intervention procedures; measurement and sample collection; adherence to intervention and barriers and facilitators to dietary and lifestyle change.

For peer review only - http://bmjopen.bmj.com/site/about/guidelines.xhtml

Secondary outcomes

Cognitive assessment

A validated version of Montreal Cognitive Assessment test (MoCA), auditory verbal learning test, categorical verbal fluency and trail making test (TMT) part B will be conducted at baseline and end of study visits.

Blood pressure

Three consecutive measurements of resting blood pressure readings will be recorded in a sitting position using a calibrated OMRON monitor. The median measure will be used to estimate the blood pressure. Blood pressure will be measured at all outcome assessment visits.

Anthropometry and body composition

Body weight will be recorded in kilograms using calibrated, electronic scales to the nearest 0.1kg where possible. Weight will be measured at all outcome assessment visits. Height will be recorded in metres using a stadiometer and measured to the nearest 0.01m where possible. The recordings for weight and height will be subsequently used to calculate BMI by dividing weight (kg) by height (m^2). BMI will be calculated at baseline and end of study visits. Body composition will be measured by bioelectrical impedance. A Tanita Body Composition analyser will be used to measure body fat percentage and classification, segmental subcutaneous fat and skeletal muscle percentage (whole body, trunk, legs and arms), resting metabolism, visceral fat level and classification and body age. Body composition will be measured at baseline and end of study visits.

Physical assessments

The following physical assessments will be conducted at baseline and end of study visits. **(1)** Muscle strength will be measured by a hand grip-strength dynamometer. Using their right arm, the participant will be asked to make three measurements and an average calculated. The same process will be repeated for the left arm. **(2)** A 4 meter walk test will be done to assess the gait speed. Two trials will be conducted and the average measure of time in minutes will be used to assess the gait speed. **(3)** In the Timed Up and Go (TUG) test, the time taken for participants to rise from an armless chair (46 cm height), to walk 3 metres, turn, walk back and sit down will be measured. The TUG test will be performed twice consecutively, and the average of the two scores will be used. **(4)** The Geriatric Depression Scale (GDS) (short form) will be used to assess depressive symptoms[40] – scores range from zero to 15, with a score of zero to five indicating a normal score and a score greater than five

For peer review only - http://bmjopen.bmj.com/site/about/guidelines.xhtml

suggesting depression. The international physical activity questionnaire (IPAQ)[41] will be used to assess physical activity.

Dietary Assessment

Conventional approaches to assessing dietary intake are associated with measurement error. There is a growing consensus that combining the use of self-report instruments (like FFQ and 24 hour recall) together with biomarker analyses, could increase the accuracy of individual intake estimates, especially for episodically consumed foods.[42 43] Thus, dietary assessment data will be collected by two methods: 24 hour recall and FFQ. Participants will complete a one day 24 hour recall facilitated by a trained data collector. The FFQ is a validated questionnaire used in previous dietary research in Malaysia.[44] Information will be entered into Nutrition Pro Software (v7.5) and daily intake will be calculated.

Biological Sample Collection

The following bio-specimen will be collected in this study:

1. Whole venous blood (15 ml)
2. 24 hour urine sample
3. Spot urine sample
4. Saliva
5. Capillary blood

All samples will be used only for the purpose of the study. The venous blood will first be centrifuged, separated into aliquots and then stored at -20°C at the KK Segamat and then transported to Monash University Sunway campus for storage. Other samples will be stored at -20°C storage. The freezer room for the stored samples will be under lock and key with restricted access.

Blood sampling

Whole blood samples will be analysed to measure changes in nitrite concentrations during the intervention. Samples will also be analysed to assess for biomarkers of cardio-vascular risk (C-reactive protein, glycated haemoglobin, nitro-tyrosine) and direct brain measures such as plasma BDNF, plasma amyloid β42 and amyloid β40. A capillary blood sample will be taken and analysed for the concentration of glucose in the blood using a portable glucometer. Dried blood spot samples will be analysed to measure changes in nitrate concentrations during the intervention. Blood spots will be first processed using a standardised elution protocol to obtain a liquid solution of the samples and

For peer review only - http://bmjopen.bmj.com/site/about/guidelines.xhtml

then analysed for nitrate concentrations using ozone-based chemiluminescence, which is the reference method for the analysis on nitrate in biological fluids. All blood sampling assessment methods will be conducted at baseline and end of study visits. Venous blood samples will be performed by a qualified medical attendant or staff nurse practising at KK Segamat and dried blood spot samples will be collected by a trained data collector.

24 hour and spot urine sampling

Both 24 hour urine and spot urine samples will be collected. 24 hour urine collection has been associated with a having high respondent burden owing to the time consuming nature of this method, particularly in a community and LMIC setting, where 24 hour urine sampling may be logistically difficult. Spot urine sampling is potentially a more convenient and affordable alternative. However, there are still a number of questions about the reliability of spot urine collection as a means of monitoring intervention adherence. Therefore, by using these two methods of urine collection, the authors hope to explore the feasibility of implementing these collection methods within the target population.[45]

Eligible participants will be provided with a kit to collect a 24 hour urine sample during their baseline home visit (part 1). This will be provided two days before their clinic visit (part 2). The kit includes: (1) A participant information booklet with written instructions on how to collect the sample; (2) A '24 hour urine collection record' to note essential information about the urine collection; (3) Urine-collecting equipment: (a) 2.5 litre screw-capped plastic container for storing the 24 hour urine sample, (b) 1 litre plastic jug which the urine will be voided into, (c) 1 litre screw-capped plastic container either as backup container when the 2.5 litre storage container is full, or for temporal collections of urine made outside the home; (4) A permanent marker pen to note the start and finish times of urine collection in the 2.5 litre container. Trained data collectors will verbally explain the method of 24 hour urine collection to participants. The 24 hour urine collection will be initiated one day before their clinic visit and end in the morning of their appointment. Participants will also collect a spot urine sample on the morning of their clinic visit. They will be provided with a 60 ml screw-capped plastic bottle for the spot urine collection. Participants will be instructed by a trained data collector to provide a spot urine sample using the midstream clean-catch technique.

The 24 hour urine samples collected will be assessed for completeness using assessment of the duration of urine collection, the total urine volume and 24 hour urinary creatinine excretion. The urine samples will be excluded from analysis if the time of the collection falls outside the range of 22–26 hours, if the total 24 hour urine volume is less than 500 ml or greater than 6000 ml, and if 24 hour creatinine excretion is less than 3 mmol, or greater than 25 mmol in women, or less than 6 mmol, or

For peer review only - http://bmjopen.bmj.com/site/about/guidelines.xhtml

greater than 30 mmol in men. Urinary sodium will be determined using the ion-selective electrode method. For nitrate, samples will be diluted 1:100 and then analysed for nitrate concentrations using ozone-based chemiluminescence, which is the reference method for the analysis on nitrate in biological fluids.

Saliva sampling

Whole saliva will be collected using the passive drool technique. 1 ml of will be collected into one collection tube. Participants will be asked to generate some saliva in their mouth and when ready, the participant should hold the adaptor with collection tube attached to their mouth and pass saliva into the tube. This process is repeated until desired amount is collected. To measure nitrate, samples will be diluted 1:100 and then analysed for nitrate concentrations using ozone-based chemiluminescence. For the salivary strips, a dedicated app freely available to download on mobile devices will be used to provide a quantitative reading of the salivary nitrite concentrations as a surrogate marker of dietary nitrate intake.

Patient and public involvement - Post-intervention qualitative evaluation

Participants will be invited to provide feedback on their participation in the intervention study via focus groups discussions. The focus group topic guide will also be piloted among a random sample of participants prior to implementation to allow feedback, refinement and tailoring prior to implementation. Two focus group discussions will be conducted within each arm of trial (total 8). Purposive sampling will be used to capture broad variation in age, ethnicity, house location and for better understanding of the phenomenon being studied. All focus groups discussions will be audio recorded and the topics covered will include: **(1)** Access to the food items - financial (affordability), physical factors; **(2)** Cultural acceptability of the recommended food items; **(3)** Family support to adhere to the dietary changes; **(4)** Adherence to the instructions - facilitators and barriers, methods to self-monitor; **(5)** Perceived benefit and side effects of intervention; **(6)** Study context - education material, frequency of visit, location of visits, data collection methods and tools; **(7)** Provision of biomedical samples - barriers, cultural acceptability, difficulty; **(8)** Likes and dislikes in relation to the new dietary options and **(9)** Preferred intervention arm. Finally, a behaviour change theoretical framework will be used to guide the qualitative analysis.[46 47] This will allow the feedback and recommendations from participants to directly inform refinements and tailoring of the intervention for a follow-on efficacy trial as well as to understand the barriers and facilitators to dietary change among this target group.

For peer review only - http://bmjopen.bmj.com/site/about/guidelines.xhtml

Sample size

A formal sample size calculation has not been performed considering the pilot, feasibility nature of the study. A sample size of 30 per group was based on the estimated effect size provided by the guidelines by Whitehead et al[48], which outlines the sample size calculation for pilot studies with the aim to maximise resources and avoid occurrence of a type II error. Specifically, a sample size greater than 25 individuals per group would provide in a 90% powered main trial the ability to detect a small effect size between 0.1 and 0.3. More than 100 participants are expected to complete the trial; i.e., an anticipated drop-out rate ≤20%.

Statistical Analysis

As the main aims of this feasibility study relate to the feasibility, acceptability and the potential to deliver a dietary intervention, these data will be reported narratively illustrated with descriptive statistics. The CONSORT 2010 statement will be used to guide the reporting of this information.[39] Secondary aims are largely descriptive, aiming to provide bounds for key parameters to inform the main trial. Intention-to-treat analysis will be used to include all randomised participants and determine the key outcome measures. The recruitment and retention rate in each arm will be described. It is unlikely that statistically significant differences in retention rates will be discovered, but upper and lower bounds will be estimated, and reasons for groups with apparently low recruitment or retention rates will be explored qualitatively. Normality of the distribution of the variables and appropriate transformations (LogX, 1/X, Xn) will be performed if necessary. Summary data will be expressed as mean (SD) or frequency (percent). General linear models for repeated measures will be used to detect significant differences between the intervention groups with and without adjustment for baseline levels. Chi square test will be used for categorical variables. An interaction term (time x group) will be built to assess between-group interactions in changes in the measured outcomes during the interventions. Dietary data will be analysed using the Nutritionist Pro software (v7.5).

Data collection supervision and training

SEACO is an ISO certified research platform for its operations, therefore training and supervision are carried out in accordance with SEACO's standard operating procedures. All data collectors are required to be fluent in the local language (Bahasa Melayu or Malay language or Mandarin) and have at least a working knowledge of English. To ensure data quality and consistency across interviewers, the field supervisor will undertake random concurrent supervisory visits to observe practice. The data collectors will also be periodically observed by the project leader and the field manager. Based on the

For peer review only - http://bmjopen.bmj.com/site/about/guidelines.xhtml

scoring completed during these observation sessions, onsite training or re-training will be carried out as appropriate. Data collection is undertaken on Samsung Galaxy tablets. The data are encrypted on the tablet to ensure they are secure and inaccessible in the unlikely event that they are mislaid by data collectors. Transfer of data from the tablet to the remote server is done on a weekly basis. The data are also encrypted on the server and backed up by servers at Monash Sunway Campus and at Monash Clayton. Research staff have limited access to anonymised data.

Data monitoring

All expected and unexpected adverse events reported by participants will be recorded in an events register and reported to the Human Research Ethics Committee. Due to the nature of the treatment products, i.e. commercially available food products, no adverse events are expected. However, if participants feel in anyway adversely affected by any foods or the principal investigator feels an adverse event necessitates cessation, the participant would be advised not to continue and the appropriate measures will be taken (i.e. record in field notes, contact research nurse and principal investigator if deemed necessary).

Ethics and Dissemination

The procedures outlined in this protocol, pertaining to the conduct, evaluation, and documentation of this study, are designed to ensure that the sponsor and investigator abide by GCP Guidelines and under the guiding principles detailed in the Declaration of Helsinki. DM and YCS have obtained GCP certification from Ministry of Health, Malaysia. Ethical approval has been granted by Monash University Human Research Ethics Committee (MUHREC) (17864) and the Malaysian Medical Research Ethics Committee (MREC) (#NMRR-19-617-45916). Results of the study will be disseminated via peer-reviewed publications and presentations at national and international conferences.

DISCUSSION

This study will demonstrate the feasibility of a dietary intervention to increase the consumption of high-nitrate green leafy vegetables and reduce salt intake over a 6-month period among Malaysian adults with raised blood pressure. The design of this feasibility study has taken into account the evidence collected in developed countries on the efficacy of complex interventions for the prevention of cognitive decline and evidence on the major transitions in dietary patterns occurring in developing countries as a result of nutrition transition trends. An increased consumption of vegetables and reduction of salt intake are key dietary recommendations for the prevention of cardio-metabolic diseases. Our hypotheses are that a targeted, educational approach, to encourage healthy dietary

For peer review only - http://bmjopen.bmj.com/site/about/guidelines.xhtml

choices focused on these key elements of the diet, would be linked to greater long-term adherence to the interventions and improved cognitive function with prospective, projected reduced risk of dementia in these populations. This mixed-method feasibility study will provide key quantitative and qualitative information on the delivery of a novel dietary intervention and will aim to estimate the effect size of the single and combined interventions on cognitive function and blood pressure, which will be instrumental in the design and calculation of the sample size of a larger, follow-on efficacy trial.

For peer review only

AUTHOR CONTRIBUTIONS

MS, DDR and DM were involved in conception and trial design. MS, AMG and AMH contributed to the intervention development and design. AMG wrote the initial manuscript draft and MS and DM contributed to writing the final manuscript and provided critical comments during revisions. All authors contributed to a critical review of the paper (AMG, DM, CPW, AMH, YCS, MASA, ZBK, ANBMG, BCMS, PA, DDR, LR and MS). DM and CPW will be responsible for overseeing the recruitment, data collection and intervention delivery.

FUNDING

This study is funded by the National Institute for Health Research (NIHR) [GHR Group: 16/137/62 – NIHR Global Health Research Group on Dementia Prevention and Enhanced Care (DePEC), Newcastle University, United Kingdom using UK aid from the UK Government to support global health research. The views expressed are those of the author(s) and not necessarily those of the NIHR or the Department of Health and Social Care. Sponsor - Monash University, Malaysia Jeffrey Cheah Sch of Med & HS, Selangor, 47500, Malaysia. Tel: +60 (0)7 2190 600 / Population Health Sciences Institute; Newcastle University, Newcastle, United Kingdom].

COMPETING INTEREST STATEMENT

"All authors have completed the ICMJE uniform disclosure form at www.icmje.org/coi_disclosure.pdf and declare: no support from any organisation for the submitted work; LR reports grants from National Institute of Health Research Senior Investigator award during the conduct of the study; no other relationships or activities that could appear to have influenced the submitted work."

For peer review only - http://bmjopen.bmj.com/site/about/guidelines.xhtml

REFERENCES

1. Robinson L, Tang E, Taylor J-P. Dementia: timely diagnosis and early intervention. *BMJ : British Medical Journal* 2015;350:h3029. doi: 10.1136/bmj.h3029
2. Scarmeas N, Anastasiou CA, Yannakoulia M. Nutrition and prevention of cognitive impairment. *The Lancet Neurology* 2018;17(11):1006-15. doi: https://doi.org/10.1016/S1474-4422(18)30338-7
3. Livingston G, Sommerlad A, Orgeta V, et al. Dementia prevention, intervention, and care. *The Lancet* 2017;390(10113):2673-734. doi: https://doi.org/10.1016/S0140-6736(17)31363-6
4. Norton S, Matthews FE, Barnes DE, et al. Potential for primary prevention of Alzheimer's disease: an analysis of population-based data. *The Lancet Neurology* 2014;13(8):788-94. doi: https://doi.org/10.1016/S1474-4422(14)70136-X
5. Forbes SC, Holroyd-Leduc JM, Poulin MJ, et al. Effect of Nutrients, Dietary Supplements and Vitamins on Cognition: a Systematic Review and Meta-Analysis of Randomized Controlled Trials. *Can Geriatr J* 2015;18(4):231-45. doi: 10.5770/cgj.18.189
6. Smith PJ, Blumenthal JA, Babyak MA, et al. Effects of the dietary approaches to stop hypertension diet, exercise, and caloric restriction on neurocognition in overweight adults with high blood pressure. *Hypertension (Dallas, Tex : 1979)* 2010;55(6):1331-38. doi: 10.1161/HYPERTENSIONAHA.109.146795 [published Online First: 2010/03/19]
7. McEvoy CT, Guyer H, Langa KM, et al. Neuroprotective Diets Are Associated with Better Cognitive Function: The Health and Retirement Study. *J Am Geriatr Soc* 2017;65(8):1857-62. doi: 10.1111/jgs.14922 [published Online First: 2017/04/25]
8. Valls-Pedret C, Sala-Vila A, Serra-Mir M, et al. Mediterranean Diet and Age-Related Cognitive Decline: A Randomized Clinical TrialMediterranean Diet and Cognitive DeclineMediterranean Diet and Cognitive Decline. *JAMA Internal Medicine* 2015;175(7):1094-103. doi: 10.1001/jamainternmed.2015.1668
9. McGrattan AM, McEvoy CT, McGuinness B, et al. Effect of dietary interventions in mild cognitive impairment: a systematic review. *Br J Nutr* 2018;120(12):1388-405. doi: 10.1017/S0007114518002945 [published Online First: 2018/11/09]
10. McGrattan AM, van Aller C, Narytnyk A, et al. Nutritional Interventions for the Prevention of Cognitive Impairment and Dementia in Developing Economies of East Asia: Systematic Review and Meta-analysis. Federation of European Nutrition Societies (FENS). Dublin, 2019.
11. Matthew K. The Nutrition Transition in Developing Asia: Dietary Change, Drivers and Health Impacts. In: Jackson P, Spiess WEL, Sultana F, eds. Eating, Drinking: Surviving: The International Year of Global Understanding - IYGU. Cham: Springer International Publishing 2016:83-90.
12. Dans A, Ng N, Varghese C, et al. The rise of chronic non-communicable diseases in southeast Asia: time for action. *The Lancet* 2011;377(9766):680-89. doi: https://doi.org/10.1016/S0140-6736(10)61506-1
13. Hord NG, Tang Y, Bryan NS. Food sources of nitrates and nitrites: the physiologic context for potential health benefits. *The American journal of clinical nutrition* 2009;90(1):1-10. doi: 10.3945/ajcn.2008.27131 [published Online First: 2009/05/15]
14. Hord NG, Tang Y, Bryan NS. Food sources of nitrates and nitrites: the physiologic context for potential health benefits. *The American Journal of Clinical Nutrition* 2009;90(1):1-10. doi: 10.3945/ajcn.2008.27131
15. Clifford T, Babateen A, Shannon OM, et al. Effects of inorganic nitrate and nitrite consumption on cognitive function and cerebral blood flow: A systematic review and meta-analysis of randomized clinical trials. *Critical Reviews in Food Science and Nutrition* 2018:1-11. doi: 10.1080/10408398.2018.1453779

For peer review only - http://bmjopen.bmj.com/site/about/guidelines.xhtml

16. Lundberg JO, Carlstrom M, Weitzberg E. Metabolic Effects of Dietary Nitrate in Health and Disease. *Cell metabolism* 2018;28(1):9-22. doi: 10.1016/j.cmet.2018.06.007 [published Online First: 2018/07/05]
17. Clifford T, Babateen A, Shannon OM, et al. Effects of inorganic nitrate and nitrite consumption on cognitive function and cerebral blood flow: A systematic review and meta-analysis of randomized clinical trials. 2018:1-11. doi: 10.1080/10408398.2018.1453779
18. Babateen AM, Fornelli G, Donini LM, et al. Assessment of dietary nitrate intake in humans: a systematic review. *The American journal of clinical nutrition* 2018;108(4):878-88. doi: 10.1093/ajcn/nqy108 [published Online First: 2018/10/16]
19. Blekkenhorst LC, Bondonno CP, Lewis JR, et al. Association of dietary nitrate with atherosclerotic vascular disease mortality: a prospective cohort study of older adult women. *The American Journal of Clinical Nutrition* 2017;106(1):207-16. doi: 10.3945/ajcn.116.146761
20. Siervo M, Lara J, Ogbonmwan I, et al. Inorganic Nitrate and Beetroot Juice Supplementation Reduces Blood Pressure in Adults: A Systematic Review and Meta-Analysis. *The Journal of Nutrition* 2013;143(6):818-26. doi: 10.3945/jn.112.170233
21. WHO. Risk reduction of cognitive decline and dementia: WHO guidelines. Geneva. . World Health Organization;, 2019:Licence: CC BY-NC-SA 3.0 IGO.
22. Farquhar WB, Edwards DG, Jurkovitz CT, et al. Dietary sodium and health: more than just blood pressure. *J Am Coll Cardiol* 2015;65(10):1042-50. doi: 10.1016/j.jacc.2014.12.039
23. Vogt TM, Appel LJ, Obarzanek E, et al. Dietary Approaches to Stop Hypertension: rationale, design, and methods. DASH Collaborative Research Group. *Journal of the American Dietetic Association* 1999;99(8 Suppl):S12-8. [published Online First: 1999/08/18]
24. Obarzanek E, Proschan MA, Vollmer WM, et al. Individual blood pressure responses to changes in salt intake: results from the DASH-Sodium trial. *Hypertension (Dallas, Tex : 1979)* 2003;42(4):459-67. doi: 10.1161/01.hyp.0000091267.39066.72 [published Online First: 2003/09/04]
25. Wengreen H, Munger RG, Cutler A, et al. Prospective study of Dietary Approaches to Stop Hypertension– and Mediterranean-style dietary patterns and age-related cognitive change: the Cache County Study on Memory, Health and Aging. *The American Journal of Clinical Nutrition* 2013;98(5):1263-71. doi: 10.3945/ajcn.112.051276
26. Tangney CC. DASH and Mediterranean-type Dietary Patterns to Maintain Cognitive Health. *Curr Nutr Rep* 2014;3(1):51-61. doi: 10.1007/s13668-013-0070-2 [published Online First: 2014/01/05]
27. Mente A, O'Donnell M, Rangarajan S, et al. Urinary sodium excretion, blood pressure, cardiovascular disease, and mortality: a community-level prospective epidemiological cohort study. *The Lancet* 2018;392(10146):496-506. doi: 10.1016/S0140-6736(18)31376-X
28. Strazzullo P, D'Elia L, Kandala NB, et al. Salt intake, stroke, and cardiovascular disease: meta-analysis of prospective studies. *Bmj* 2009;339:b4567. doi: 10.1136/bmj.b4567 [published Online First: 2009/11/26]
29. He FJ, Li J, MacGregor GA. Effect of longer term modest salt reduction on blood pressure: Cochrane systematic review and meta-analysis of randomised trials. *BMJ : British Medical Journal* 2013;346:f1325. doi: 10.1136/bmj.f1325
30. He FJ, MacGregor GA. Role of salt intake in prevention of cardiovascular disease: controversies and challenges. *Nat Rev Cardiol* 2018;15(6):371-77. doi: 10.1038/s41569-018-0004-1
31. He FJ, Pombo-Rodrigues S, MacGregor GA. Salt reduction in England from 2003 to 2011: its relationship to blood pressure, stroke and ischaemic heart disease mortality. *BMJ Open* 2014;4(4):e004549. doi: 10.1136/bmjopen-2013-004549
32. Afshin A, Sur PJ, Fay KA, et al. Health effects of dietary risks in 195 countries, 1990–2017: a systematic analysis for the Global Burden of Disease Study 2017. *The Lancet* 2019;393(10184):1958-72. doi: 10.1016/S0140-6736(19)30041-8

For peer review only - http://bmjopen.bmj.com/site/about/guidelines.xhtml

33. Trieu K, Neal B, Hawkes C, et al. Salt Reduction Initiatives around the World – A Systematic Review of Progress towards the Global Target. *PLOS ONE* 2015;10(7):e0130247. doi: 10.1371/journal.pone.0130247
34. Dong OM. Excessive dietary sodium intake and elevated blood pressure: a review of current prevention and management strategies and the emerging role of pharmaconutrigenetics. *BMJ Nutrition, Prevention & Health* 2018:bmjnph-2018-000004. doi: 10.1136/bmjnph-2018-000004
35. Chan A-W, Tetzlaff JM, Altman DG, et al. SPIRIT 2013 statement: defining standard protocol items for clinical trials. *Ann Intern Med* 2013;158(3):200-07. doi: 10.7326/0003-4819-158-3-201302050-00583
36. Chen J, Tian Y, Liao Y, et al. Salt-restriction-spoon improved the salt intake among residents in China. *PloS one* 2013;8(11):e78963-e63. doi: 10.1371/journal.pone.0078963
37. Malaysia MoH. Salt Reduction Strategy: To Prevent and Control NCD for Malaysia 2015-2020. In: Division NDC, ed. MOH Malaysia, 2015.
38. Malaysia MoH. Malaysian Dietary Guidelines. In: Malaysia MoH, ed. Malaysia: National Coordinating Committee on Food and Nutrition, 2010:1-261.
39. Eldridge SM, Chan CL, Campbell MJ, et al. CONSORT 2010 statement: extension to randomised pilot and feasibility trials. *BMJ* 2016;355:i5239. doi: 10.1136/bmj.i5239
40. Sheikh JI, Yesavage JA. Geriatric Depression Scale (GDS): Recent evidence and development of a shorter version. US: Haworth Press, 1986:165-73.
41. Craig CL, Marshall AL, Sjöström M, et al. International Physical Activity Questionnaire: 12-Country Reliability and Validity. *Medicine & Science in Sports & Exercise* 2003;35(8):1381-95. doi: 10.1249/01.Mss.0000078924.61453.Fb
42. Freedman LS, Midthune D, Arab L, et al. Combining a Food Frequency Questionnaire With 24-Hour Recalls to Increase the Precision of Estimation of Usual Dietary Intakes—Evidence From the Validation Studies Pooling Project. *American Journal of Epidemiology* 2018;187(10):2227-32. doi: 10.1093/aje/kwy126
43. Gormley IC, Bai Y, Brennan L. Combining biomarker and self-reported dietary intake data: A review of the state of the art and an exposition of concepts. *Statistical methods in medical research* 2020;29(2):617-35. doi: 10.1177/0962280219837698 [published Online First: 2019/04/05]
44. (IPH) IfPH. National Health and Morbidity Survey 2014: Malaysian Adult Nutrition Survey. Vol. 1: Methodology and General Findings. 2014:1-108.
45. Campbell NRC, He FJ, Tan M, et al. The International Consortium for Quality Research on Dietary Sodium/Salt (TRUE) position statement on the use of 24-hour, spot, and short duration (<24 hours) timed urine collections to assess dietary sodium intake. *The Journal of Clinical Hypertension* 2019;21(6):700-09. doi: 10.1111/jch.13551
46. Michie S, van Stralen MM, West R. The behaviour change wheel: a new method for characterising and designing behaviour change interventions. *Implement Sci* 2011;6:42-42. doi: 10.1186/1748-5908-6-42
47. Atkins L, Francis J, Islam R, et al. A guide to using the Theoretical Domains Framework of behaviour change to investigate implementation problems. *Implementation Science* 2017;12(1):77. doi: 10.1186/s13012-017-0605-9
48. Whitehead AL, Julious SA, Cooper CL, et al. Estimating the sample size for a pilot randomised trial to minimise the overall trial sample size for the external pilot and main trial for a continuous outcome variable. *Statistical methods in medical research* 2016;25(3):1057-73. doi: 10.1177/0962280215588241 [published Online First: 2015/06/21]

For peer review only - http://bmjopen.bmj.com/site/about/guidelines.xhtml

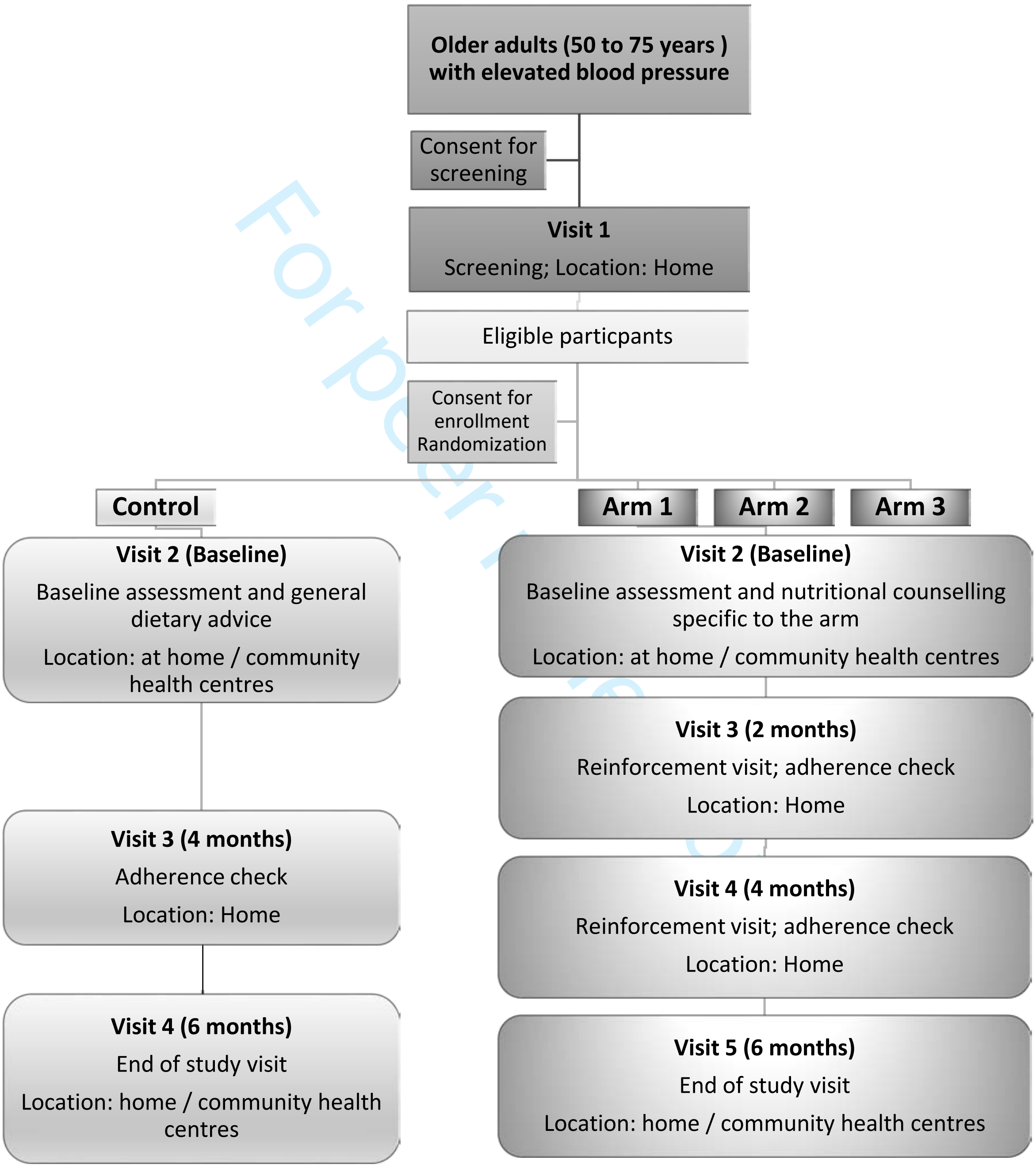

Figure 1: A flow diagram providing an overview of the study procedures

Reporting checklist for protocol of a clinical trial.

Based on the SPIRIT guidelines.

Instructions to authors

Complete this checklist by entering the page numbers from your manuscript where readers will find each of the items listed below.

Your article may not currently address all the items on the checklist. Please modify your text to include the missing information. If you are certain that an item does not apply, please write "n/a" and provide a short explanation.

Upload your completed checklist as an extra file when you submit to a journal.

In your methods section, say that you used the SPIRITreporting guidelines, and cite them as:

Chan A-W, Tetzlaff JM, Altman DG, Laupacis A, Gøtzsche PC, Krleža-Jerić K, Hróbjartsson A, Mann H, Dickersin K, Berlin J, Doré C, Parulekar W, Summerskill W, Groves T, Schulz K, Sox H, Rockhold FW, Rennie D, Moher D. SPIRIT 2013 Statement: Defining standard protocol items for clinical trials. Ann Intern Med. 2013;158(3):200-207

		Reporting Item	Page Number
**Administrative information**			
Title	#1	Descriptive title identifying the study design, population, interventions, and, if applicable, trial acronym	1

For peer review only

Trial registration	#2a	Trial identifier and registry name. If not yet registered, name of intended registry	1
Trial registration: data set	#2b	All items from the World Health Organization Trial Registration Data Set	
Protocol version	#3	Date and version identifier	DePEC protocol v1.0 22/10/2019
Funding	#4	Sources and types of financial, material, and other support	19
Roles and responsibilities: contributorship	#5a	Names, affiliations, and roles of protocol contributors	1 & 19
Roles and responsibilities: sponsor contact information	#5b	Name and contact information for the trial sponsor	19
Roles and responsibilities: sponsor and funder	#5c	Role of study sponsor and funders, if any, in study design; collection, management, analysis, and interpretation of data; writing of the report; and the decision to submit the report for publication, including whether they will have ultimate authority over any of these activities	19

For peer review only

Roles and responsibilities: committees	#5d	Composition, roles, and responsibilities of the coordinating centre, steering committee, endpoint adjudication committee, data management team, and other individuals or groups overseeing the trial, if applicable (see Item 21a for data monitoring committee)	n/a
**Introduction**			
Background and rationale	#6a	Description of research question and justification for undertaking the trial, including summary of relevant studies (published and unpublished) examining benefits and harms for each intervention	4-7
Background and rationale: choice of comparators	#6b	Explanation for choice of comparators	4-7
Objectives	#7	Specific objectives or hypotheses	7
Trial design	#8	Description of trial design including type of trial (eg, parallel group, crossover, factorial, single group), allocation ratio, and framework (eg, superiority, equivalence, non-inferiority, exploratory)	11
**Methods: Participants, interventions, and outcomes**			

For peer review only

Study setting	#9	Description of study settings (eg, community clinic, academic hospital) and list of countries where data will be collected. Reference to where list of study sites can be obtained	8
Eligibility criteria	#10	Inclusion and exclusion criteria for participants. If applicable, eligibility criteria for study centres and individuals who will perform the interventions (eg, surgeons, psychotherapists)	8-9
Interventions: description	#11a	Interventions for each group with sufficient detail to allow replication, including how and when they will be administered	11
Interventions: modifications	#11b	Criteria for discontinuing or modifying allocated interventions for a given trial participant (eg, drug dose change in response to harms, participant request, or improving / worsening disease)	n/a
Interventions: adherance	#11c	Strategies to improve adherence to intervention protocols, and any procedures for monitoring adherence (eg, drug tablet return; laboratory tests)	n/a
Interventions: concomitant care	#11d	Relevant concomitant care and interventions that are permitted or prohibited during the trial	n/a
Outcomes	#12	Primary, secondary, and other outcomes, including the specific measurement variable (eg, systolic blood pressure), analysis metric (eg, change from baseline,	11-16

For peer review only

		final value, time to event), method of aggregation (eg, median, proportion), and time point for each outcome. Explanation of the clinical relevance of chosen efficacy and harm outcomes is strongly recommended	
Participant timeline	#13	Time schedule of enrolment, interventions (including any run-ins and washouts), assessments, and visits for participants. A schematic diagram is highly recommended (see Figure)	10 & 12
Sample size	#14	Estimated number of participants needed to achieve study objectives and how it was determined, including clinical and statistical assumptions supporting any sample size calculations	16
Recruitment	#15	Strategies for achieving adequate participant enrolment to reach target sample size	8
**Methods: Assignment of interventions (for controlled trials)**			
Allocation: sequence generation	#16a	Method of generating the allocation sequence (eg, computer-generated random numbers), and list of any factors for stratification. To reduce predictability of a random sequence, details of any planned restriction (eg, blocking) should be provided in a	11

For peer review only

		separate document that is unavailable to those who enrol participants or assign interventions	
Allocation concealment mechanism	#16b	Mechanism of implementing the allocation sequence (eg, central telephone; sequentially numbered, opaque, sealed envelopes), describing any steps to conceal the sequence until interventions are assigned	11
Allocation: implementation	#16c	Who will generate the allocation sequence, who will enrol participants, and who will assign participants to interventions	11
Blinding (masking)	#17a	Who will be blinded after assignment to interventions (eg, trial participants, care providers, outcome assessors, data analysts), and how	11
Blinding (masking): emergency unblinding	#17b	If blinded, circumstances under which unblinding is permissible, and procedure for revealing a participant's allocated intervention during the trial	11
**Methods: Data collection, management, and analysis**			
Data collection plan	#18a	Plans for assessment and collection of outcome, baseline, and other trial data, including any related processes to promote data quality (eg, duplicate measurements, training of assessors) and a	17

For peer review only

		description of study instruments (eg, questionnaires, laboratory tests) along with their reliability and validity, if known. Reference to where data collection forms can be found, if not in the protocol	
Data collection plan: retention	#18b	Plans to promote participant retention and complete follow-up, including list of any outcome data to be collected for participants who discontinue or deviate from intervention protocols	17
Data management	#19	Plans for data entry, coding, security, and storage, including any related processes to promote data quality (eg, double data entry; range checks for data values). Reference to where details of data management procedures can be found, if not in the protocol	17
Statistics: outcomes	#20a	Statistical methods for analysing primary and secondary outcomes. Reference to where other details of the statistical analysis plan can be found, if not in the protocol	17
Statistics: additional analyses	#20b	Methods for any additional analyses (eg, subgroup and adjusted analyses)	17
Statistics: analysis population and missing data	#20c	Definition of analysis population relating to protocol non-adherence (eg, as randomised analysis), and any statistical methods to handle missing data (eg, multiple imputation)	17

For peer review only

Methods: Monitoring

Data monitoring: formal committee	#21a	Composition of data monitoring committee (DMC); summary of its role and reporting structure; statement of whether it is independent from the sponsor and competing interests; and reference to where further details about its charter can be found, if not in the protocol. Alternatively, an explanation of why a DMC is not needed	n/a
Data monitoring: interim analysis	#21b	Description of any interim analyses and stopping guidelines, including who will have access to these interim results and make the final decision to terminate the trial	n/a
Harms	#22	Plans for collecting, assessing, reporting, and managing solicited and spontaneously reported adverse events and other unintended effects of trial interventions or trial conduct	17
Auditing	#23	Frequency and procedures for auditing trial conduct, if any, and whether the process will be independent from investigators and the sponsor	17

Ethics and dissemination

Research ethics approval	#24	Plans for seeking research ethics committee / institutional review board (REC / IRB) approval	18

For peer review only

Protocol amendments	#25	Plans for communicating important protocol modifications (eg, changes to eligibility criteria, outcomes, analyses) to relevant parties (eg, investigators, REC / IRBs, trial participants, trial registries, journals, regulators)	n/a
Consent or assent	#26a	Who will obtain informed consent or assent from potential trial participants or authorised surrogates, and how (see Item 32)	8
Consent or assent: ancillary studies	#26b	Additional consent provisions for collection and use of participant data and biological specimens in ancillary studies, if applicable	n/a
Confidentiality	#27	How personal information about potential and enrolled participants will be collected, shared, and maintained in order to protect confidentiality before, during, and after the trial	18
Declaration of interests	#28	Financial and other competing interests for principal investigators for the overall trial and each study site	1
Data access	#29	Statement of who will have access to the final trial dataset, and disclosure of contractual agreements that limit such access for investigators	17-18
Ancillary and post trial care	#30	Provisions, if any, for ancillary and post-trial care, and for compensation to those who suffer harm from trial participation	17-18

For peer review only

Dissemination policy: trial results	#31a	Plans for investigators and sponsor to communicate trial results to participants, healthcare professionals, the public, and other relevant groups (eg, via publication, reporting in results databases, or other data sharing arrangements), including any publication restrictions	17-18
Dissemination policy: authorship	#31b	Authorship eligibility guidelines and any intended use of professional writers	n/a
Dissemination policy: reproducible research	#31c	Plans, if any, for granting public access to the full protocol, participant-level dataset, and statistical code	n/a
**Appendices**			
Informed consent materials	#32	Model consent form and other related documentation given to participants and authorised surrogates	n/a
Biological specimens	#33	Plans for collection, laboratory evaluation, and storage of biological specimens for genetic or molecular analysis in the current trial and for future use in ancillary studies, if applicable	n/a

None The SPIRIT checklist is distributed under the terms of the Creative Commons Attribution License CC-BY-ND 3.0. This checklist can be completed online using https://www.goodreports.org/, a tool made by the EQUATOR Network in collaboration with Penelope.ai

For peer review only

BMJ Open

The feasibility and acceptability of a dietary intervention study to reduce salt intake and increase high-nitrate vegetable consumption among middle-aged and older Malaysian adults with elevated blood pressure: a study protocol

Journal:	*BMJ Open*
Manuscript ID	bmjopen-2019-035453.R2
Article Type:	Protocol
Date Submitted by the Author:	04-Jun-2020
Complete List of Authors:	McGrattan, Andrea; Newcastle University, Population Health Sciences Institute<br>Mohan, Devi; Monash University - Malaysia Campus, Global Public Health, School of Medicine<br>Chua, Pei Wei; Monash University - Malaysia Campus, Global Public Health. Jeffrey Cheah School of Medicine and Health Sciences; South East Asia Community Observatory (SEACO)<br>Mat Hussin, Azizah; Universiti Kuala Lumpur, Kampus Cawangan Institute of Medical Science Technology<br>Soh, Yee Chang; Monash University - Malaysia Campus, Global Public Health; Jeffrey Cheah School of Medicine; South East Asia Community Observatory (SEACO)<br>Alawad, Mawada; Monash University - Malaysia Campus, Global Public Health. Jeffrey Cheah School of Medicine and Health Sciences; South East Asia Community Observatory (SEACO)<br>bin Kassim, Zaid; District Health Office (PKD) Segamat<br>bin Mohd Ghazali, Ahmad Nizal; District Health Office (PKD) Segamat<br>Stephan, Blossom; University of Nottingham, School of Medicine<br>Allotey, Pascale; Monash University - Malaysia Campus, Global Public Health. Jeffrey Cheah School of Medicine and Health Sciences; United Nations University International Institute for Global Health<br>Reidpath, Daniel; Monash University - Malaysia Campus, Global Public Health. Jeffrey Cheah School of Medicine and Health Sciences; South East Asia Community Observatory (SEACO)<br>Robinson, Louise; Newcastle University, Population Health Sciences Institute<br>Siervo, Mario; University of Nottingham, School of Life Sciences
**Primary Subject Heading**:	Public health
Secondary Subject Heading:	Nutrition and metabolism, Global health, Research methods
Keywords:	Dementia < NEUROLOGY, NUTRITION & DIETETICS, PUBLIC HEALTH

SCHOLARONE™
Manuscripts

I, the Submitting Author has the right to grant and does grant on behalf of all authors of the Work (as defined in the below author licence), an exclusive licence and/or a non-exclusive licence for contributions from authors who are: i) UK Crown employees; ii) where BMJ has agreed a CC-BY licence shall apply, and/or iii) in accordance with the terms applicable for US Federal Government officers or employees acting as part of their official duties; on a worldwide, perpetual, irrevocable, royalty-free basis to BMJ Publishing Group Ltd ("BMJ") its licensees and where the relevant Journal is co-owned by BMJ to the co-owners of the Journal, to publish the Work in this journal and any other BMJ products and to exploit all rights, as set out in our licence.

The Submitting Author accepts and understands that any supply made under these terms is made by BMJ to the Submitting Author unless you are acting as an employee on behalf of your employer or a postgraduate student of an affiliated institution which is paying any applicable article publishing charge ("APC") for Open Access articles. Where the Submitting Author wishes to make the Work available on an Open Access basis (and intends to pay the relevant APC), the terms of reuse of such Open Access shall be governed by a Creative Commons licence – details of these licences and which Creative Commons licence will apply to this Work are set out in our licence referred to above.

Other than as permitted in any relevant BMJ Author's Self Archiving Policies, I confirm this Work has not been accepted for publication elsewhere, is not being considered for publication elsewhere and does not duplicate material already published. I confirm all authors consent to publication of this Work and authorise the granting of this licence.

For peer review only

DePEC protocol R2. 02/06/2020

TITLE

The feasibility and acceptability of a dietary intervention study to reduce salt intake and increase high-nitrate vegetable consumption among middle-aged and older Malaysian adults with elevated blood pressure: a study protocol

AUTHORS

Andrea M McGrattan[1*], Devi Mohan[2], Chua Pei Wei[2,7], Azizah Mat Hussin[3], Yee Chang Soh[2,7], Mawada Azhari Sidahmed Alawad[2,7], Zaid Bin Kassim[4], Ahmad Nizal Bin Mohd Ghazali[4], Blossom C M Stephan[5], Pascale Allotey[2,6], Daniel D Reidpath[2,7], Louise Robinson[1], Mario Siervo[8]

AFFILIATIONS

[1]Population Health Sciences Institute, Newcastle University, Newcastle, United Kingdom.

[2]Global Public Health. Jeffrey Cheah School of Medicine and Health Sciences, Monash University Malaysia, Selangor, Malaysia.

[3]Universiti Kuala Lumpur, Kampus Cawangan Institute of Medical Science Technology, Kuala Lumpur, Malaysia.

[4]District Health Office (PKD) Segamat, Jalan Gudang Ubat 85000, Segamat Johor, Malaysia

[5] School of Medicine, The University of Nottingham, United Kingdom.

[6] Director of the United Nations University International Institute for Global Health (UNU-IIGH), Jalan Yaacob Latiff, 56000 Cheras, Kuala Lumpur, Malaysia.

[7] South East Asia Community Observatory (SEACO), Segamat, Johor, Malaysia.

[8] School of Life Sciences, The University of Nottingham Medical School, Nottingham, United Kingdom.

*Corresponding author: Dr Andrea McGrattan; Population Health Sciences Institute; Newcastle University, Newcastle, United Kingdom; andrea.mcgrattan@newcastle.ac.uk ; +44 (0) 191 208 5467

The material presented in this manuscript is original and it has not been submitted for publication elsewhere while under consideration by BMJ Open

Abstract Word Count: 300

Main Text Word Count: 5,683

References: 47

Tables: 2

Figures: 1

Online supplementary material: 0

For peer review only - http://bmjopen.bmj.com/site/about/guidelines.xhtml

DePEC protocol R2. 02/06/2020

ABSTRACT

Introduction

Global population ageing is one the key factors linked to the projected rise of dementia incidence. Hence, there is a clear need to identify strategies to overcome this expected health burden and have a meaningful impact on populations' health worldwide. Current evidence supports the role of modifiable dietary and lifestyle risk factors in reducing the risk of dementia. In South-East Asia, changes in eating and lifestyle patterns under the influence of westernised habits, have resulted in significant increases in the prevalence of metabolic, cardiovascular and neurodegenerative non-communicable diseases (NCDs). Low vegetable consumption and high sodium intake have been identified as key contributors to the increased prevalence of NCDs in these countries. Therefore, nutritional and lifestyle strategies targeting these dietary risk factors are warranted. The overall objective of this randomised feasibility trial is to demonstrate the acceptability of a dietary intervention to increase the consumption of high-nitrate green leafy vegetables and reduce salt intake over 6 months among Malaysian adults with raised blood pressure.

Methods and Analysis

Primary outcomes focus on feasibility measures of recruitment, retention, implementation and acceptability of the intervention. Secondary outcomes will include blood pressure, cognitive function, body composition and physical function (including muscle strength and gait speed). Adherence to the dietary intervention will be assessed through collection of biological samples, 24 hour recall and food frequency questionnaire. A subgroup of participants will also complete post-intervention focus groups to further explore the feasibility considerations of executing a larger trial, the ability of these individuals to make dietary changes and the barriers and facilitators associated with implementing these changes.

Ethics and Dissemination

Ethical approval has been obtained from MUHREC (Monash University Human Research Ethics Committee) and MREC (Medical Research and Ethics Committee) of Malaysia. Results of the study will be disseminated via peer-reviewed publications and presentations at national and international conferences. Trial Registration Number: ISRCTN47562685

For peer review only - http://bmjopen.bmj.com/site/about/guidelines.xhtml

ARTICLE SUMMARY

Strengths and limitations of the study

1. Feasibility of the first 2*2 trial testing single and combined effects of dietary nitrate and salt reduction for the prevention of cognitive decline in developing countries
2. Longest high-nitrate based intervention ever conducted
3. Use of objective biomarkers for the assessment of adherence to the dietary intervention and internal validation of the dietary assessment methods used (Urinary sodium – 24 hr urine *vs* spot urine / Nitrate levels in blood - dried blood spots *vs* plasma, and ELISA *vs* gold standard / Nitrate in saliva – salivary strip (point of care test) *vs* ELISA using saliva samples)
4. Mixed-method approach to obtain key information on the feasibility of the dietary intervention
5. Owing to the small sample size and the feasibility nature of this study, the planned comparative analysis is only exploratory, and therefore, the efficacy of the intervention between groups cannot be determined. However, the results of this study will be instrumental in the design and calculation of the sample size of a larger, follow-on efficacy trial

INTRODUCTION

Dementia is characterised by multiple cognitive deficits and loss of independence, and its pathogenesis is linked to multiple distinct neuropathological processes including Alzheimer's disease (AD) and/or vascular pathology.[1] Several of the pathological processes underlying dementia might be delayed, or prevented by, interventions focussed on dietary and behavioural changes[2] and, in the absence of definite pharmacological treatments, these options represent a key strategy to alleviate its individual and social impact.[3] Optimal control of modifiable cardiovascular factors such as blood pressure, and adherence to lifestyle recommendations, have been consistently associated with reduced dementia risk in observational studies.[2-4] The role of nutrition for dementia prevention has been extensively investigated by testing the role of single nutrients (i.e. n-3 PUFA or B vitamins[5]) or dietary patterns (i.e. Dietary Approaches to Stop Hypertension (DASH) diet[6] or Mediterranean diet[7 8]). There are a small number of RCTs available to support the effect of diet on cognition in Mild Cognitive Impairment[9] and among those at risk of cognitive decline or cognitively healthy populations[10]; however the trials have a small sample size and overall modest quality.

Malaysia is experiencing rapid socio-economic and nutritional transitions[11]. Changes in individual eating and lifestyle patterns and rise in food availability and financial prosperity of the population, have resulted in significant increases in the prevalence of chronic metabolic, cardiovascular and

For peer review only - http://bmjopen.bmj.com/site/about/guidelines.xhtml

neurodegenerative non-communicable diseases [NCD][12]. Effective public health interventions for the prevention of dementia are challenging in countries with limited social and health care resources which are unable to meet the growing demands of ageing populations. A systematic review of 22 randomised controlled trials, investigated the effect of nutritional interventions on cognitive performance in developing economies of East Asia and the majority of the studies showed some significant beneficial effects of the nutritional interventions on cognitive performance. However, these effects were not consistent across all of the neuropsychological tests used in each study[10]. Therefore, there is a need for well-designed nutritional intervention studies for the early prevention of cognitive decline and dementia.

Recent analyses have identified inorganic nitrate as a potential nutrient improving vascular and metabolic health.[13] Beetroot and green leafy vegetables, such as spinach, lettuce and rocket, are a key source of naturally available inorganic nitrate, containing over 250 mg of nitrate per 100 g of fresh vegetable weight.[14] Inorganic nitrate is closely linked to the metabolism of Nitric Oxide (NO) which is known for its multiple effects on physiological functions such blood pressure, brain function and immunity.[15] An increase in inorganic nitrate consumption has been associated with an increase in NO production via complex mechanisms involving oral microbiota, gastric environment and activation of reducing enzymes[16]. Recent epidemiological studies and clinical trials have demonstrated the association of increased nitrate intake with improved blood pressure, metabolic health and cognitive function.[17 18] A 15-year prospective cohort study investigated the association of consumption of high nitrate vegetables and risk of atherosclerotic vascular disease (ASVD) mortality. Results showed that a consumption of approximately 30 mg/day higher nitrate intake from vegetables was associated with a 21% lower risk of ASVD mortality. This nitrate amount equates to approximately 10–30 g/day of nitrate-rich vegetables.[19] However, the impact on cognition is still unknown with studies of short intervention durations and among small samples, and limited studies conducted in developing countries.[14 17] Nevertheless, with hypertension highlighted as an important modifiable risk factor for dementia,[3 20] more studies that test the implementation of dietary interventions that aim to improve blood pressure are warranted.

Sodium is the main electrolyte contained in extracellular fluids and involved in regulation of blood pressure and cellular activities. An excess sodium intake has been linked to extracellular volume expansion and increased blood pressure.[21] The DASH diet has been specifically developed to promote consumption of healthy dietary choices alongside strictly controlled sodium intake.[22] Secondary analyses derived from seminal DASH studies showed additional benefits on blood pressure and cardio-

For peer review only - http://bmjopen.bmj.com/site/about/guidelines.xhtml

metabolic health in individuals with lower salt intake and adherence to a healthy dietary pattern[23]. These analyses have been extended to cognitive function with promising results,[24 25] but the evidence has mostly come from studies conducted in the United States and no studies to our knowledge have been conducted in Low and Middle Income Countries (LMICs). Epidemiological studies have repeatedly proven the association of high salt diets with impaired blood pressure control and increased cardiovascular risk;[26 27] this evidence has been confirmed in several clinical trials testing the effects of salt-reducing interventions and demonstrating the protective effects of salt reduction on cardiovascular health.[28 29] A prospective study looked at the relationship between a reduction in salt intake with blood pressure, mortality from stroke and ischaemic heart disease (IHD). [30] A decrease in salt intake by 1.4 g/day, as measured by 24 hour urinary sodium, contributed to a reduction in stroke and IHD mortality, and in addition, among individuals who were not on antihypertensive medication, there was significant a fall in systolic blood pressure of 2.7±0.34mmHg.[30] Excessive sodium consumption is recognised as a global health issue[31] with efforts aiming to reduce salt intake at the population level by 30% by 2025. Seventy-five countries have already implemented national sodium reduction programmes to help meet this target.[32]

In sum, an increased consumption of dietary nitrate and lower salt intake may have additional benefits on health outcomes such as blood pressure and cognition, mediated by an increase in NO generation (i.e., nitrate) and control of vascular tone and cellular metabolism (i.e., sodium). However, this hypothesis has never been tested in previous interventions which represents the main aim of the feasibility study proposed in this protocol. The Global Health Dementia Prevention and Enhanced Care (DePEC) project is a National Institute for Health Research (NIHR) funded study with a key goal to develop approaches for dementia prevention and develop more efficient post-diagnostic care in LMICs. The DePEC-Nutrition feasibility study (presented here) is one of five work streams within the overall DePEC project.

Purpose and Aims

The overall objective of the DePEC-Nutrition feasibility study is to demonstrate the acceptability of a dietary intervention to increase the consumption in high-nitrate green leafy vegetables and reduce salt intake over a 6-month period among Malaysian adults with raised blood pressure. The objectives focus on the core areas of a trial that need to work for the study to succeed procedurally, such as recruitment ability and participant retention, data collection procedures and assessment methods used, potential to deliver the dietary intervention and resource requirement. Information on the

For peer review only - http://bmjopen.bmj.com/site/about/guidelines.xhtml

effect size of the intervention on cognition and blood pressure will also be determined, which will be instrumental in the design and calculation of the sample size of a follow-on efficacy trial.

METHODS AND ANALYSIS

Study design

The DePEC-Nutrition feasibility study is a 6 month randomised 2*2 factorial trial including four parallel arms: (1) control, (2) high-nitrate vegetable consumption, (3) low salt consumption and (4) combined high-nitrate vegetable consumption plus low salt consumption. The trial will be conducted among 120 middle-aged and older Malaysian adults. Sponsor and grant number: GHR Group: 16/137/62 – NIHR Global Health Research Group on Dementia Prevention and Enhanced Care (DePEC), Newcastle University, United Kingdom. This trial has been registered with ISRCTN (ISRCTN47562685). The SPIRIT reporting guidelines were used to guide the preparation of this protocol.[33]

Participants

This study is embedded within the South East Asia Community Observatory (SEACO) population (http://www.seaco.asia/). SEACO is a health and demographic surveillance site (HDSS); a unique research platform in population health and wellbeing focused on a middle-income community in Segamat, Johor in the southern tip of the Peninsular Malaysia. The district of Segamat comprises urban, rural and plantation areas with an ethnic mix including Malays, Chinese and Indian, in similar proportions to the national population. SEACO runs its HDSS research platform in five sub-districts (mukim) of the Segamat district: Bekok, Chaah, Gemereh, Jabi and Sungai Segamat. The SEACO population was established using a baseline census (March 2012 to February 2013) that included information on sociodemographic status and self-reported health conditions. Consenting participants underwent detailed health profile assessments in the first health round in 2013-2014. The next census was done in 2017, with a total number of 40,015 participants completing the assessment. Individuals were re-seen after one year (in 2018) for the second health round. Individuals aged 50 years and over, who are living in Segamat, Johor and have been assessed as part of SEACO during the health round in 2018 will form the study population for the DePEC-Nutrition feasibility study. The baseline assessment will be carried out in the Klink Kesihatans (KK) in Sungai Segamat sub–district (a community health clinic in Segamat, Johor). Hence, only participants from within 5 km radius of KK Segamat will be included in the study population. A total of 2433 participants have been identified on the database as potentially eligible for this study by broad application of the study criteria (mean age (standard deviation) - 61.27 (6.77) years and number of males – n=1078 (44.3%). This subset of particiapnts will

For peer review only - http://bmjopen.bmj.com/site/about/guidelines.xhtml

be screened in detail, and then will be informed of the study and invited for a screening visit as outlined in the study procedures.

Recruitment

The community will be informed about this study via a local Community Engagement Committee (CEC) who meet regularly to distribute information on projects planned within SEACO. Participants will be randomly identified by the application of the inclusion and exclusion criteria to the SEACO health surveillance database. Potential participants will be approached via a home visit and will be provided with information about the study. They will be given an opportunity to read detailed information about the study procedures and can ask questions. Thereafter, their interest of participating in the study will be evaluated. If they agree to take part, a screening assessment will be completed. If eligible, participants will be invited to attend a baseline study visit at the health facilities within the community.

Eligibility criteria

To be eligible for inclusion in this study, participants must report as:

1. Male and female participants with an age between 50 and 75 years from the SEACO database
2. Pre-hypertensive, stage 1 hypertensive or diagnosed hypertensive: Any person with a self-reported history of hypertension (on or not on medication) or with elevated blood pressure (SBP 120-159 mmHg or DBP 80-99 mmHg)
3. Not severely cognitively impaired (defined as a Mini Mental Examination Status [MMSE] score of 19 or over)

Participants will be excluded from the study if they:

1. Are participating in other research clinical studies
2. Are unable to provide consent
3. Have systolic blood pressure more than or equal to 160 mmHg and / or diastolic blood pressure more than or equal to 100 mmHg
4. Adhere to any therapeutic diet such as weight loss treatments or a gluten-free diet
5. Are vegan (these individuals are likely to have a high intake of vegetables). Since Indian participants are more likely to follow a vegetarian or semi-vegetarian diet, in order to maintain representativeness across ethnic groups included in the study, only participants who are vegan will be excluded from the study
6. Participants with a body mass index (BMI) less than 18.5 kg/m^2
7. Have a history of active cancer or any diagnosis of cancer in the last 5 years
8. Have a history of excessive alcohol intake (>21 units of alcohol per week)

For peer review only - http://bmjopen.bmj.com/site/about/guidelines.xhtml

9. Have a history of brain damage or significant head trauma (resulting in a loss of consciousness)
10. Have a diagnosis of acute and chronic medical conditions interfering with the study outcomes such as systemic infections (tuberculosis, hepatitis B, HIV/AIDS), severe liver and kidney diseases, inflammatory bowel diseases, coronary heart diseases, cerebrovascular diseases or is a diabetic on insulin therapy
11. Have had any major surgical procedures (in the past six weeks or planned in the next year) that could interfere with the study outcomes
12. Have current diagnosis of moderate or severe depression, or other serious mental or brain disorder and / or are currently on psychiatric medication (antidepressants, sedatives, antipsychotics)
13. Regularly uses sodium-altering drugs (angiotensin-converting-enzyme (ACE) inhibitors, angiotensin receptor blockers (ARB), corticosteroids, diuretics, hormonal therapies (oestrogens, thyroxin, and progesterone), non-steroidal anti-inflammatory drugs (NSAIDs), proton-pump inhibitors), organic nitrates
14. Has changed in anti-hypertensive medication regimen for the previous 3 months
15. Have limited mobility due to any reason
16. Are planning to move house within a year

Study procedure

Data collection will be undertaken directly on an electronic hand held device (Samsung Galaxy Tab 3v – Survey CTO). Questionnaire items will be read out-loud by the data collector and participant responses will be recorded directly onto the electronic tablet. The flow of participants through the DePEC-Nutrition feasibility study is illustrated in Figure 1. At the screening visit, written informed consent will be first obtained, after which participants will complete a screening questionnaire to collect information on demographics, co-morbidities, medication history, cognitive function (MMSE), body weight, height and resting blood pressure. If eligible, participants will undergo baseline assessments, consisting of two parts; a home visit (part 1), followed by an appointment at KK, Segamat (part 2). Baseline assessments will include: obtaining respondent's signed informed consent for study participation, eligibility assessment (medical and medication history), dietary assessment (24-hour recall and food frequency questionnaire (FFQ)), cognitive assessment, collection of biological samples (blood, dried blood spots, 24-urine and spot urine samples, saliva samples, and salivary strips), measurements of hand grip and gait speed, physical activity (IPAQ) and depression (GDS).

For peer review only - http://bmjopen.bmj.com/site/about/guidelines.xhtml

An interim two-month visit will take place for those in the intervention groups only, to provide reinforcement messages relating to the allocated dietary intervention and with a view to enhancing compliance with the intervention. In addition, a brief adherence questionnaire will be administered, body weight and resting blood pressure will be measured and salivary strip and spot urine samples will be collected. The same measurements and further dietary reinforcement messages will be conducted at an interim four-month visit for the participants allocated to the intervention arms. Participants in the control arm will only have one interim visit at month four and will have their weight and blood pressure measured. The end of study visit (at KK, Segamat) will include the conduction of the measurements collected at baseline, including cognitive assessment, dietary assessment, and collection of biological samples (blood, dried blood spots, 24-urine and spot urine samples, saliva samples, salivary strips). In addition, an exit group session (focus group evaluation) and a self-administered feedback questionnaire will be scheduled to evaluate the adherence to the interventions and obtain detailed feedback on the overall study protocol. Finally, the participants will be offered a token of appreciation to acknowledge their participation in the study and to compensate for travel expenses. This is usually to the value of RM 25.

Randomisation and blinding

The SEACO health round database will serve as the sampling frame, from which eligible participants are identified. Block randomisation will be carried out to assign eligible participants into one of the four arms using R software (randomizeR)[34]. Block sizes of four participants will be used in the randomisation and will be generated by a member of the research team not involved in the data collection. The interviewers will be blinded to the participant assignment to intervention arms. Participant blinding to the intervention allocation will not be possible due to the nature of the dietary intervention study. Matching of characteristics for group allocation will not be used in this study. The distribution of characteristics, such as age or gender, will be part of the feasibility assessment to gain information on the characteristics of the population willing to participate without imposing any restrictions on the intention to participate in the study. This information will be used to evaluate whether specific randomisation approaches will need to be used in future studies to ensure even distribution of key characteristics between groups.

Intervention and control arms

This dietary and behavioural intervention will focus on key components that are related to changing local dietary patterns influenced by nutritional transition trends in Malaysia. It aims to target two key components that have protective (dietary nitrate) and negative (salt intake) effects on cardiovascular

For peer review only - http://bmjopen.bmj.com/site/about/guidelines.xhtml

and cognitive health. The intervention will target individuals with a higher CVD risk to adopt a tailored strategy to enhance daily consumption of green leafy vegetables and / or reduce salt intake. Nutritional counselling sessions with a medical doctor will be conducted at baseline to provide sustainable and effective strategies to improve dietary habits as well as monitor and address key barriers to dietary change. These sessions will be delivered in a group setting using PowerPoint slides and practical activities to educate the group on the allocated dietary intervention. All intervention groups will receive written information focussed on health benefits, sources, recommended intakes and practical tips related to one of the following dietary interventions; (1) to increase dietary nitrate (2) to reduce salt intake (3) to increase dietary nitrate and to reduce salt intake. Those who are randomised to the salt intervention (either alone or combined) will receive a measuring spoon as an additional strategy to support individuals to understand portion size and measure salt intake, as successfully tested by an intervention study in China.[35] Furthermore, the Ministry for Health Malaysia set out in their Salt Reduction Strategy 2015-2020 how reducing the amount of salt and sauces consumed in the home is a key priority.[36] This provides justification for inclusion of the salt restriction spoon as an intervention strategy. The intervention groups will also receive bi-weekly text message reminders to provide reinforcement of the key dietary behaviour change messages relevant for their group allocation. Compliance will be monitored during the intermediate visits (2 and 4 months, respectively) where participants will also receive reinforcement video messages to remind participants of the key dietary advice that was discussed during the counselling sessions at baseline. Those allocated to the control group (4) will receive written information on general healthy eating and lifestyle advice based on the Malaysian dietary guidelines.[37]

Outcome measures

Outcome assessments will be conducted at baseline, month four and month six for all groups, with an additional assessment visit at month two for those in the intervention groups. Assessments at baseline and month 6 will be conducted in clinic / at home, with remaining assessments completed in the participants own home. The same instruments will be used for measurements at each time point. Assessors are trained prior to the assessments to follow a standardised protocol. A timeline of the study procedures can be found in Table 1.

For peer review only - http://bmjopen.bmj.com/site/about/guidelines.xhtml

Table 1: Timeline of study procedures

**Procedures**	**Consent/Screening**	**Baseline Week 1**	**Week 2**	**Week 4**	**Week 6**	**Week 8**	**Week 10**	**Week 12**	**Week 16**	**Week 18**	**Week 20**	**Week 22**	**End of study Week 24**
**Inclusion/Exclusion**	✓												
**Consent**	✓												
**Activities for intervention group**													
Dietary education		✓											
Reinforcement at home visit		✓				✓			✓				
Group education counselling session		✓											
Home visit						✓			✓				
SMS messages			✓	✓	✓	✓	✓	✓	✓	✓	✓	✓	✓
**Activities for control group**													
General health promotion message		✓											
Home visit									✓				
**Study Measurements**													
Sociodemographic		✓											
Concomitant medication check list		✓				✓							✓
Any medical events		✓				✓							✓
Physical activity (IPAQ)		✓											✓
Depression (GDS)		✓											✓
Food frequency questionnaire (FFQ)		✓											✓
24hr dietary recall		✓											✓
Nitrate: Urine		✓**a**				✓***b**			✓**b**				✓**a**
Salt: Urine		✓**a**				✓***b**			✓**b**				✓**a**
Cognitive performance		✓											✓
Resting blood pressure		✓				✓*			✓				✓
Body composition		✓				✓***c**			✓**c**				✓
Physical function		✓											✓
Blood Sample and dried blood spot		✓											✓
Saliva sample and salivary strips		✓				✓*‡			✓‡				✓

a: 24-urine and spot urine samples; b: spot urine sample only; c: body weight only; * intervention groups only; ‡ saliva strips only

For peer review only - http://bmjopen.bmj.com/site/about/guidelines.xhtml

Primary outcome

The primary outcome measures are related to feasibility and will be collected via questionnaire, review of the study procedures and by review of data collector field notes. Patient feedback, acceptance and adherence issues will be additionally assessed through qualitative focus groups conducted post-intervention. The specific feasibility considerations that will be assessed in the study have been outlined in Table 2. The CONSORT 2010 statement[38] will be used to guide the reporting of the feasibility results.

Table 2: Primary outcome: Feasibility considerations to be assessed

**Feasibility Considerations**	
**Recruitment**	1. Recruitment rates<br>2. Time taken for the recruitment of participants<br>3. Characteristics of recruited sample as against expected<br>4. Non-response rates<br>5. Reasons for non-response
**Retention and Completion**	1. Retention rates<br>2. Number of drops and reasons for withdrawal<br>3. Follow-up response rates (2 months and 4 months follow-ups)
**Data Collection methods**<br>Feasibility of measurement tools	1. Time taken to fill in questionnaires<br>2. Missing data from questionnaires<br>3. Understanding level of procedures and measurement protocols<br>4. Suitability of outcome measures<br>5. Sensitivity of outcome measures to change<br>6. Internal consistency of outcome measures - validation of data collection methods (24 hours vs spot urine; saliva samples vs salivary strips)
Resource Capacity	1. Data collection and measurement equipment<br>2. Field support (transportation, IT, etc.)<br>3. Personnel (were the right numbers of people with the right skills available when they were needed)<br>4. Duration of each phase
**Acceptability and adherence to intervention**	1. Barriers to participation (collected at screening interview)<br>2. Burden (reasons for not taking part/discontinuation or dropping out)<br>3. Qualitative enquiry/feedback from participants and research staff<br>4. Focus group evaluation - This part of the feasibility trial will separately examine the acceptability and suitability of: intervention materials; intervention timing; intervention procedures; measurement and sample collection; adherence to intervention and barriers and facilitators to dietary and lifestyle change.

For peer review only

For peer review only - http://bmjopen.bmj.com/site/about/guidelines.xhtml

DePEC protocol R2. 02/06/2020

Secondary outcomes

Cognitive assessment

A validated version of Montreal Cognitive Assessment test (MoCA), auditory verbal learning test, categorical verbal fluency and trail making test (TMT) part B will be conducted at baseline and end of study visits.

Blood pressure

Three consecutive measurements of resting blood pressure readings will be recorded in a sitting position using a calibrated OMRON automated monitor (OMRON HEM 907, OMRON Healthcare, Milton Keynes United Kingdom). The median measure will be used to estimate the blood pressure. Blood pressure will be measured at all outcome assessment visits.

Anthropometry and body composition

Body weight will be recorded in kilograms using calibrated, electronic scales to the nearest 0.1kg where possible. Weight will be measured at all outcome assessment visits. Height will be recorded in metres using a stadiometer and measured to the nearest 0.01m where possible. The recordings for weight and height will be subsequently used to calculate BMI by dividing weight (kg) by height (m^2). BMI will be calculated at baseline and end of study visits. Body composition will be measured by bioelectrical impedance. A Tanita Body Composition analyser will be used to measure body fat percentage and classification, segmental subcutaneous fat and skeletal muscle percentage (whole body, trunk, legs and arms), resting metabolism, visceral fat level and classification and body age. Body composition will be measured at baseline and end of study visits.

Physical assessments

The following physical assessments will be conducted at baseline and end of study visits. **(1)** Muscle strength will be measured by a hand grip-strength dynamometer. Using their right arm, the participant will be asked to make three measurements and an average calculated. The same process will be repeated for the left arm. **(2)** A 4 meter walk test will be done to assess the gait speed. Two trials will be conducted and the average measure of time in minutes will be used to assess the gait speed. **(3)** In the Timed Up and Go (TUG) test, the time taken for participants to rise from an armless chair (46 cm height), to walk 3 metres, turn, walk back and sit down will be measured. The TUG test will be performed twice consecutively, and the average of the two scores will be used. **(4)** The Geriatric Depression Scale (GDS) (short form) will be used to assess depressive symptoms[39] – scores range from zero to 15, with a score of zero to five indicating a normal score and a score greater than five

For peer review only - http://bmjopen.bmj.com/site/about/guidelines.xhtml

suggesting depression. The international physical activity questionnaire (IPAQ)[40] will be used to assess physical activity.

Dietary Assessment

Conventional approaches to assessing dietary intake are associated with measurement error. There is a growing consensus that combining the use of self-report instruments (like FFQ and 24 hour recall) together with biomarker analyses, could increase the accuracy of individual intake estimates, especially for episodically consumed foods.[41 42] Thus, dietary assessment data will be collected by two methods: 24 hour recall and FFQ. Participants will complete a one day 24 hour recall facilitated by a trained data collector. The FFQ is a validated questionnaire used in previous dietary research in Malaysia.[43] Information will be entered into Nutrition Pro Software (v7.5) and daily intake will be calculated.

Biological Sample Collection

The following bio-specimen will be collected in this study:

1. Whole venous blood (15 ml)
2. 24 hour urine sample
3. Spot urine sample
4. Saliva
5. Capillary blood

All samples will be used only for the purpose of the study. The venous blood will first be centrifuged, separated into aliquots and then stored at -20°C at the KK Segamat and then transported to Monash University Sunway campus for storage. Other samples will be stored at -20°C storage. The freezer room for the stored samples will be under lock and key with restricted access.

Blood sampling

Whole blood samples will be analysed to measure changes in nitrite concentrations during the intervention. Samples will also be analysed to assess for biomarkers of cardio-vascular risk (C-reactive protein, glycated haemoglobin, nitro-tyrosine) and direct brain measures such as plasma BDNF, plasma amyloid β42 and amyloid β40. A capillary blood sample will be taken and analysed for the concentration of glucose in the blood using a portable glucometer. Dried blood spot samples will be analysed to measure changes in nitrate concentrations during the intervention. Blood spots will be first processed using a standardised elution protocol to obtain a liquid solution of the samples and

For peer review only - http://bmjopen.bmj.com/site/about/guidelines.xhtml

then analysed for nitrate concentrations using ozone-based chemiluminescence, which is the reference method for the analysis on nitrate in biological fluids. All blood sampling assessment methods will be conducted at baseline and end of study visits. Venous blood samples will be performed by a qualified medical attendant or staff nurse practising at KK Segamat and dried blood spot samples will be collected by a trained data collector.

24 hour and spot urine sampling

Both 24 hour urine and spot urine samples will be collected. 24 hour urine collection has been associated with a having high respondent burden owing to the time consuming nature of this method, particularly in a community and LMIC setting, where 24 hour urine sampling may be logistically difficult. Spot urine sampling is potentially a more convenient and affordable alternative. However, there are still a number of questions about the reliability of spot urine collection as a means of monitoring intervention adherence. Therefore, by using these two methods of urine collection, the authors hope to explore the feasibility of implementing these collection methods within the target population.[44]

Eligible participants will be provided with a kit to collect a 24 hour urine sample during their baseline home visit (part 1). This will be provided two days before their clinic visit (part 2). The kit includes: (1) A participant information booklet with written instructions on how to collect the sample; (2) A '24 hour urine collection record' to note essential information about the urine collection; (3) Urine-collecting equipment: (a) 2.5 litre screw-capped plastic container for storing the 24 hour urine sample, (b) 1 litre plastic jug which the urine will be voided into, (c) 1 litre screw-capped plastic container either as backup container when the 2.5 litre storage container is full, or for temporal collections of urine made outside the home; (4) A permanent marker pen to note the start and finish times of urine collection in the 2.5 litre container. Trained data collectors will verbally explain the method of 24 hour urine collection to participants. The 24 hour urine collection will be initiated one day before their clinic visit and end in the morning of their appointment. Participants will also collect a spot urine sample on the morning of their clinic visit. They will be provided with a 60 ml screw-capped plastic bottle for the spot urine collection. Participants will be instructed by a trained data collector to provide a spot urine sample using the midstream clean-catch technique.

The 24 hour urine samples collected will be assessed for completeness using assessment of the duration of urine collection, the total urine volume and 24 hour urinary creatinine excretion. The urine samples will be excluded from analysis if the time of the collection falls outside the range of 22–26 hours, if the total 24 hour urine volume is less than 500 ml or greater than 6000 ml, and if 24 hour creatinine excretion is less than 3 mmol, or greater than 25 mmol in women, or less than 6 mmol, or

For peer review only - http://bmjopen.bmj.com/site/about/guidelines.xhtml

greater than 30 mmol in men. Urinary sodium will be determined using the ion-selective electrode method. For nitrate, samples will be diluted 1:100 and then analysed for nitrate concentrations using ozone-based chemiluminescence, which is the reference method for the analysis on nitrate in biological fluids.

Saliva sampling

Whole saliva will be collected using the passive drool technique. 1 ml of will be collected into one collection tube. Participants will be asked to generate some saliva in their mouth and when ready, the participant should hold the adaptor with collection tube attached to their mouth and pass saliva into the tube. This process is repeated until desired amount is collected. To measure nitrate, samples will be diluted 1:100 and then analysed for nitrate concentrations using ozone-based chemiluminescence. For the salivary strips, a dedicated app freely available to download on mobile devices will be used to provide a quantitative reading of the salivary nitrite concentrations as a surrogate marker of dietary nitrate intake.

Patient and public involvement - Post-intervention qualitative evaluation

Participants will be invited to provide feedback on their participation in the intervention study via focus groups discussions. The focus group topic guide will also be piloted among a random sample of participants prior to implementation to allow feedback, refinement and tailoring prior to implementation. Two focus group discussions will be conducted within each arm of trial (total 8). Purposive sampling will be used to capture broad variation in age, ethnicity, house location and for better understanding of the phenomenon being studied. All focus groups discussions will be audio recorded and the topics covered will include: **(1)** Access to the food items - financial (affordability), physical factors; **(2)** Cultural acceptability of the recommended food items; **(3)** Family support to adhere to the dietary changes; **(4)** Adherence to the instructions - facilitators and barriers, methods to self-monitor; **(5)** Perceived benefit and side effects of intervention; **(6)** Study context - education material, frequency of visit, location of visits, data collection methods and tools; **(7)** Provision of biomedical samples - barriers, cultural acceptability, difficulty; **(8)** Likes and dislikes in relation to the new dietary options and **(9)** Preferred intervention arm. Finally, a behaviour change theoretical framework will be used to guide the qualitative analysis.[45 46] This will allow the feedback and recommendations from participants to directly inform refinements and tailoring of the intervention for a follow-on efficacy trial as well as to understand the barriers and facilitators to dietary change among this target group.

For peer review only - http://bmjopen.bmj.com/site/about/guidelines.xhtml

Sample size

A formal sample size calculation has not been performed considering the pilot, feasibility nature of the study. A sample size of 30 per group was based on the estimated effect size provided by the guidelines by Whitehead et al[47], which outlines the sample size calculation for pilot studies with the aim to maximise resources and avoid occurrence of a type II error. Specifically, a sample size greater than 25 individuals per group would provide in a 90% powered main trial the ability to detect a small effect size between 0.1 and 0.3. More than 100 participants are expected to complete the trial; i.e., an anticipated drop-out rate ≤20%.

Statistical Analysis

As the main aims of this feasibility study relate to the feasibility, acceptability and the potential to deliver a dietary intervention, these data will be reported narratively illustrated with descriptive statistics. The CONSORT 2010 statement will be used to guide the reporting of this information.[38] Secondary aims are largely descriptive, aiming to provide bounds for key parameters to inform the main trial. Intention-to-treat analysis will be used to include all randomised participants and determine the key outcome measures. The recruitment and retention rate in each arm will be described. It is unlikely that statistically significant differences in retention rates will be discovered, but upper and lower bounds will be estimated, and reasons for groups with apparently low recruitment or retention rates will be explored qualitatively. Normality of the distribution of the variables and appropriate transformations (LogX, 1/X, Xn) will be performed if necessary. Summary data will be expressed as mean (SD) or frequency (percent). General linear models for repeated measures will be used to detect significant differences between the intervention groups with and without adjustment for baseline levels. Chi square test will be used for categorical variables. An interaction term (time x group) will be built to assess between-group interactions in changes in the measured outcomes during the interventions. Dietary data will be analysed using the Nutritionist Pro software (v7.5).

Data collection supervision and training

SEACO is an ISO certified research platform for its operations, therefore training and supervision are carried out in accordance with SEACO’s standard operating procedures. All data collectors are required to be fluent in the local language (Bahasa Melayu or Malay language or Mandarin) and have at least a working knowledge of English. To ensure data quality and consistency across interviewers, the field supervisor will undertake random concurrent supervisory visits to observe practice. The data collectors will also be periodically observed by the project leader and the field manager. Based on the

For peer review only - http://bmjopen.bmj.com/site/about/guidelines.xhtml

For peer review only

scoring completed during these observation sessions, onsite training or re-training will be carried out as appropriate. Data collection is undertaken on Samsung Galaxy tablets. The data are encrypted on the tablet to ensure they are secure and inaccessible in the unlikely event that they are mislaid by data collectors. Transfer of data from the tablet to the remote server is done on a weekly basis. The data are also encrypted on the server and backed up by servers at Monash Sunway Campus and at Monash Clayton. Research staff have limited access to anonymised data.

Data monitoring

All expected and unexpected adverse events reported by participants will be recorded in an events register and reported to the Human Research Ethics Committee. Due to the nature of the treatment products, i.e. commercially available food products, no adverse events are expected. However, if participants feel in anyway adversely affected by any foods or the principal investigator feels an adverse event necessitates cessation, the participant would be advised not to continue and the appropriate measures will be taken (i.e. record in field notes, contact research nurse and principal investigator if deemed necessary).

Ethics and Dissemination

The procedures outlined in this protocol, pertaining to the conduct, evaluation, and documentation of this study, are designed to ensure that the sponsor and investigator abide by GCP Guidelines and under the guiding principles detailed in the Declaration of Helsinki. DM and YCS have obtained GCP certification from Ministry of Health, Malaysia. Ethical approval has been granted by Monash University Human Research Ethics Committee (MUHREC) (17864) and the Malaysian Medical Research Ethics Committee (MREC) (#NMRR-19-617-45916). Results of the study will be disseminated via peer-reviewed publications and presentations at national and international conferences.

DISCUSSION

This study will demonstrate the feasibility of a dietary intervention to increase the consumption of high-nitrate green leafy vegetables and reduce salt intake over a 6-month period among Malaysian adults with raised blood pressure. The design of this feasibility study has taken into account the evidence collected in developed countries on the efficacy of complex interventions for the prevention of cognitive decline and evidence on the major transitions in dietary patterns occurring in developing countries as a result of nutrition transition trends. An increased consumption of vegetables and reduction of salt intake are key dietary recommendations for the prevention of cardio-metabolic diseases. Our hypotheses are that a targeted, educational approach, to encourage healthy dietary

For peer review only - http://bmjopen.bmj.com/site/about/guidelines.xhtml

choices focused on these key elements of the diet, would be linked to greater long-term adherence to the interventions and improved cognitive function with prospective, projected reduced risk of dementia in these populations. This mixed-method feasibility study will provide key quantitative and qualitative information on the delivery of a novel dietary intervention and will aim to estimate the effect size of the single and combined interventions on cognitive function and blood pressure, which will be instrumental in the design and calculation of the sample size of a larger, follow-on efficacy trial.

Figure 1: A flow diagram providing an overview of the study procedures

For peer review only - http://bmjopen.bmj.com/site/about/guidelines.xhtml

AUTHOR CONTRIBUTIONS

MS, DDR and DM were involved in conception and trial design. MS, AMG and AMH contributed to the intervention development and design. AMG wrote the initial manuscript draft and MS and DM contributed to writing the final manuscript and provided critical comments during revisions. All authors contributed to a critical review of the paper (AMG, DM, CPW, AMH, YCS, MASA, ZBK, ANBMG, BCMS, PA, DDR, LR and MS). DM and CPW will be responsible for overseeing the recruitment, data collection and intervention delivery.

FUNDING

This study is funded by the National Institute for Health Research (NIHR) [GHR Group: 16/137/62 – NIHR Global Health Research Group on Dementia Prevention and Enhanced Care (DePEC), Newcastle University, United Kingdom using UK aid from the UK Government to support global health research. The views expressed are those of the author(s) and not necessarily those of the NIHR or the Department of Health and Social Care. Sponsor - Monash University, Malaysia Jeffrey Cheah Sch of Med & HS, Selangor, 47500, Malaysia. Tel: +60 (0)7 2190 600 / Population Health Sciences Institute; Newcastle University, Newcastle, United Kingdom].

COMPETING INTEREST STATEMENT

"All authors have completed the ICMJE uniform disclosure form at www.icmje.org/coi_disclosure.pdf and declare: no support from any organisation for the submitted work; LR reports grants from National Institute of Health Research Senior Investigator award during the conduct of the study; no other relationships or activities that could appear to have influenced the submitted work."

For peer review only

DePEC protocol R2. 02/06/2020

REFERENCES

1. Robinson L, Tang E, Taylor J-P. Dementia: timely diagnosis and early intervention. *BMJ : British Medical Journal* 2015;350:h3029. doi: 10.1136/bmj.h3029
2. Scarmeas N, Anastasiou CA, Yannakoulia M. Nutrition and prevention of cognitive impairment. *The Lancet Neurology* 2018;17(11):1006-15. doi: https://doi.org/10.1016/S1474-4422(18)30338-7
3. Livingston G, Sommerlad A, Orgeta V, et al. Dementia prevention, intervention, and care. *The Lancet* 2017;390(10113):2673-734. doi: https://doi.org/10.1016/S0140-6736(17)31363-6
4. Norton S, Matthews FE, Barnes DE, et al. Potential for primary prevention of Alzheimer's disease: an analysis of population-based data. *The Lancet Neurology* 2014;13(8):788-94. doi: https://doi.org/10.1016/S1474-4422(14)70136-X
5. Forbes SC, Holroyd-Leduc JM, Poulin MJ, et al. Effect of Nutrients, Dietary Supplements and Vitamins on Cognition: a Systematic Review and Meta-Analysis of Randomized Controlled Trials. *Can Geriatr J* 2015;18(4):231-45. doi: 10.5770/cgj.18.189
6. Smith PJ, Blumenthal JA, Babyak MA, et al. Effects of the dietary approaches to stop hypertension diet, exercise, and caloric restriction on neurocognition in overweight adults with high blood pressure. *Hypertension (Dallas, Tex : 1979)* 2010;55(6):1331-38. doi: 10.1161/HYPERTENSIONAHA.109.146795 [published Online First: 2010/03/19]
7. McEvoy CT, Guyer H, Langa KM, et al. Neuroprotective Diets Are Associated with Better Cognitive Function: The Health and Retirement Study. *J Am Geriatr Soc* 2017;65(8):1857-62. doi: 10.1111/jgs.14922 [published Online First: 2017/04/25]
8. Valls-Pedret C, Sala-Vila A, Serra-Mir M, et al. Mediterranean Diet and Age-Related Cognitive Decline: A Randomized Clinical TrialMediterranean Diet and Cognitive DeclineMediterranean Diet and Cognitive Decline. *JAMA Internal Medicine* 2015;175(7):1094-103. doi: 10.1001/jamainternmed.2015.1668
9. McGrattan AM, McEvoy CT, McGuinness B, et al. Effect of dietary interventions in mild cognitive impairment: a systematic review. *Br J Nutr* 2018;120(12):1388-405. doi: 10.1017/S0007114518002945 [published Online First: 2018/11/09]
10. McGrattan AM, van Aller C, Narytnyk A, et al. Nutritional Interventions for the Prevention of Cognitive Impairment and Dementia in Developing Economies of East Asia: Systematic Review and Meta-analysis. Federation of European Nutrition Societies (FENS). Dublin, 2019.
11. Matthew K. The Nutrition Transition in Developing Asia: Dietary Change, Drivers and Health Impacts. In: Jackson P, Spiess WEL, Sultana F, eds. Eating, Drinking: Surviving: The International Year of Global Understanding - IYGU. Cham: Springer International Publishing 2016:83-90.
12. Dans A, Ng N, Varghese C, et al. The rise of chronic non-communicable diseases in southeast Asia: time for action. *The Lancet* 2011;377(9766):680-89. doi: https://doi.org/10.1016/S0140-6736(10)61506-1
13. Hord NG, Tang Y, Bryan NS. Food sources of nitrates and nitrites: the physiologic context for potential health benefits. *The American journal of clinical nutrition* 2009;90(1):1-10. doi: 10.3945/ajcn.2008.27131 [published Online First: 2009/05/15]
14. Hord NG, Tang Y, Bryan NS. Food sources of nitrates and nitrites: the physiologic context for potential health benefits. *The American Journal of Clinical Nutrition* 2009;90(1):1-10. doi: 10.3945/ajcn.2008.27131
15. Clifford T, Babateen A, Shannon OM, et al. Effects of inorganic nitrate and nitrite consumption on cognitive function and cerebral blood flow: A systematic review and meta-analysis of randomized clinical trials. *Critical Reviews in Food Science and Nutrition* 2018:1-11. doi: 10.1080/10408398.2018.1453779

For peer review only - http://bmjopen.bmj.com/site/about/guidelines.xhtml

16. Lundberg JO, Carlstrom M, Weitzberg E. Metabolic Effects of Dietary Nitrate in Health and Disease. *Cell metabolism* 2018;28(1):9-22. doi: 10.1016/j.cmet.2018.06.007 [published Online First: 2018/07/05]
17. Clifford T, Babateen A, Shannon OM, et al. Effects of inorganic nitrate and nitrite consumption on cognitive function and cerebral blood flow: A systematic review and meta-analysis of randomized clinical trials. 2018:1-11. doi: 10.1080/10408398.2018.1453779
18. Babateen AM, Fornelli G, Donini LM, et al. Assessment of dietary nitrate intake in humans: a systematic review. *The American journal of clinical nutrition* 2018;108(4):878-88. doi: 10.1093/ajcn/nqy108 [published Online First: 2018/10/16]
19. Blekkenhorst LC, Bondonno CP, Lewis JR, et al. Association of dietary nitrate with atherosclerotic vascular disease mortality: a prospective cohort study of older adult women. *The American Journal of Clinical Nutrition* 2017;106(1):207-16. doi: 10.3945/ajcn.116.146761
20. WHO. Risk reduction of cognitive decline and dementia: WHO guidelines. Geneva. . World Health Organization;, 2019:Licence: CC BY-NC-SA 3.0 IGO.
21. Farquhar WB, Edwards DG, Jurkovitz CT, et al. Dietary sodium and health: more than just blood pressure. *J Am Coll Cardiol* 2015;65(10):1042-50. doi: 10.1016/j.jacc.2014.12.039
22. Vogt TM, Appel LJ, Obarzanek E, et al. Dietary Approaches to Stop Hypertension: rationale, design, and methods. DASH Collaborative Research Group. *Journal of the American Dietetic Association* 1999;99(8 Suppl):S12-8. [published Online First: 1999/08/18]
23. Obarzanek E, Proschan MA, Vollmer WM, et al. Individual blood pressure responses to changes in salt intake: results from the DASH-Sodium trial. *Hypertension (Dallas, Tex : 1979)* 2003;42(4):459-67. doi: 10.1161/01.hyp.0000091267.39066.72 [published Online First: 2003/09/04]
24. Wengreen H, Munger RG, Cutler A, et al. Prospective study of Dietary Approaches to Stop Hypertension– and Mediterranean-style dietary patterns and age-related cognitive change: the Cache County Study on Memory, Health and Aging. *The American Journal of Clinical Nutrition* 2013;98(5):1263-71. doi: 10.3945/ajcn.112.051276
25. Tangney CC. DASH and Mediterranean-type Dietary Patterns to Maintain Cognitive Health. *Curr Nutr Rep* 2014;3(1):51-61. doi: 10.1007/s13668-013-0070-2 [published Online First: 2014/01/05]
26. Mente A, O'Donnell M, Rangarajan S, et al. Urinary sodium excretion, blood pressure, cardiovascular disease, and mortality: a community-level prospective epidemiological cohort study. *The Lancet* 2018;392(10146):496-506. doi: 10.1016/S0140-6736(18)31376-X
27. Strazzullo P, D'Elia L, Kandala NB, et al. Salt intake, stroke, and cardiovascular disease: meta-analysis of prospective studies. *Bmj* 2009;339:b4567. doi: 10.1136/bmj.b4567 [published Online First: 2009/11/26]
28. He FJ, Li J, MacGregor GA. Effect of longer term modest salt reduction on blood pressure: Cochrane systematic review and meta-analysis of randomised trials. *BMJ : British Medical Journal* 2013;346:f1325. doi: 10.1136/bmj.f1325
29. He FJ, MacGregor GA. Role of salt intake in prevention of cardiovascular disease: controversies and challenges. *Nat Rev Cardiol* 2018;15(6):371-77. doi: 10.1038/s41569-018-0004-1
30. He FJ, Pombo-Rodrigues S, MacGregor GA. Salt reduction in England from 2003 to 2011: its relationship to blood pressure, stroke and ischaemic heart disease mortality. *BMJ Open* 2014;4(4):e004549. doi: 10.1136/bmjopen-2013-004549
31. Afshin A, Sur PJ, Fay KA, et al. Health effects of dietary risks in 195 countries, 1990–2017: a systematic analysis for the Global Burden of Disease Study 2017. *The Lancet* 2019;393(10184):1958-72. doi: 10.1016/S0140-6736(19)30041-8
32. Trieu K, Neal B, Hawkes C, et al. Salt Reduction Initiatives around the World – A Systematic Review of Progress towards the Global Target. *PLOS ONE* 2015;10(7):e0130247. doi: 10.1371/journal.pone.0130247

For peer review only - http://bmjopen.bmj.com/site/about/guidelines.xhtml

33. Chan A-W, Tetzlaff JM, Altman DG, et al. SPIRIT 2013 statement: defining standard protocol items for clinical trials. *Ann Intern Med* 2013;158(3):200-07. doi: 10.7326/0003-4819-158-3-201302050-00583
34. Uschner D, Schindler D, Hilgers R-D, et al. randomizeR : An R Package for the Assessment and Implementation of Randomization in Clinical Trials. *Journal of Statistical Software* 2018;85 doi: 10.18637/jss.v085.i08
35. Chen J, Tian Y, Liao Y, et al. Salt-restriction-spoon improved the salt intake among residents in China. *PloS one* 2013;8(11):e78963-e63. doi: 10.1371/journal.pone.0078963
36. Malaysia MoH. Salt Reduction Strategy: To Prevent and Control NCD for Malaysia 2015-2020. In: Division NDC, ed. MOH Malaysia, 2015.
37. Malaysia MoH. Malaysian Dietary Guidelines. In: Malaysia MoH, ed. Malaysia: National Coordinating Committee on Food and Nutrition, 2010:1-261.
38. Eldridge SM, Chan CL, Campbell MJ, et al. CONSORT 2010 statement: extension to randomised pilot and feasibility trials. *BMJ* 2016;355:i5239. doi: 10.1136/bmj.i5239
39. Sheikh JI, Yesavage JA. Geriatric Depression Scale (GDS): Recent evidence and development of a shorter version. US: Haworth Press, 1986:165-73.
40. Craig CL, Marshall AL, Sjöström M, et al. International Physical Activity Questionnaire: 12-Country Reliability and Validity. *Medicine & Science in Sports & Exercise* 2003;35(8):1381-95. doi: 10.1249/01.Mss.0000078924.61453.Fb
41. Freedman LS, Midthune D, Arab L, et al. Combining a Food Frequency Questionnaire With 24-Hour Recalls to Increase the Precision of Estimation of Usual Dietary Intakes—Evidence From the Validation Studies Pooling Project. *American Journal of Epidemiology* 2018;187(10):2227-32. doi: 10.1093/aje/kwy126
42. Gormley IC, Bai Y, Brennan L. Combining biomarker and self-reported dietary intake data: A review of the state of the art and an exposition of concepts. *Statistical methods in medical research* 2020;29(2):617-35. doi: 10.1177/0962280219837698 [published Online First: 2019/04/05]
43. (IPH) IfPH. National Health and Morbidity Survey 2014: Malaysian Adult Nutrition Survey. Vol. 1: Methodology and General Findings. 2014:1-108.
44. Campbell NRC, He FJ, Tan M, et al. The International Consortium for Quality Research on Dietary Sodium/Salt (TRUE) position statement on the use of 24-hour, spot, and short duration (<24 hours) timed urine collections to assess dietary sodium intake. *The Journal of Clinical Hypertension* 2019;21(6):700-09. doi: 10.1111/jch.13551
45. Michie S, van Stralen MM, West R. The behaviour change wheel: a new method for characterising and designing behaviour change interventions. *Implement Sci* 2011;6:42-42. doi: 10.1186/1748-5908-6-42
46. Atkins L, Francis J, Islam R, et al. A guide to using the Theoretical Domains Framework of behaviour change to investigate implementation problems. *Implementation Science* 2017;12(1):77. doi: 10.1186/s13012-017-0605-9
47. Whitehead AL, Julious SA, Cooper CL, et al. Estimating the sample size for a pilot randomised trial to minimise the overall trial sample size for the external pilot and main trial for a continuous outcome variable. *Statistical methods in medical research* 2016;25(3):1057-73. doi: 10.1177/0962280215588241 [published Online First: 2015/06/21]

For peer review only - http://bmjopen.bmj.com/site/about/guidelines.xhtml

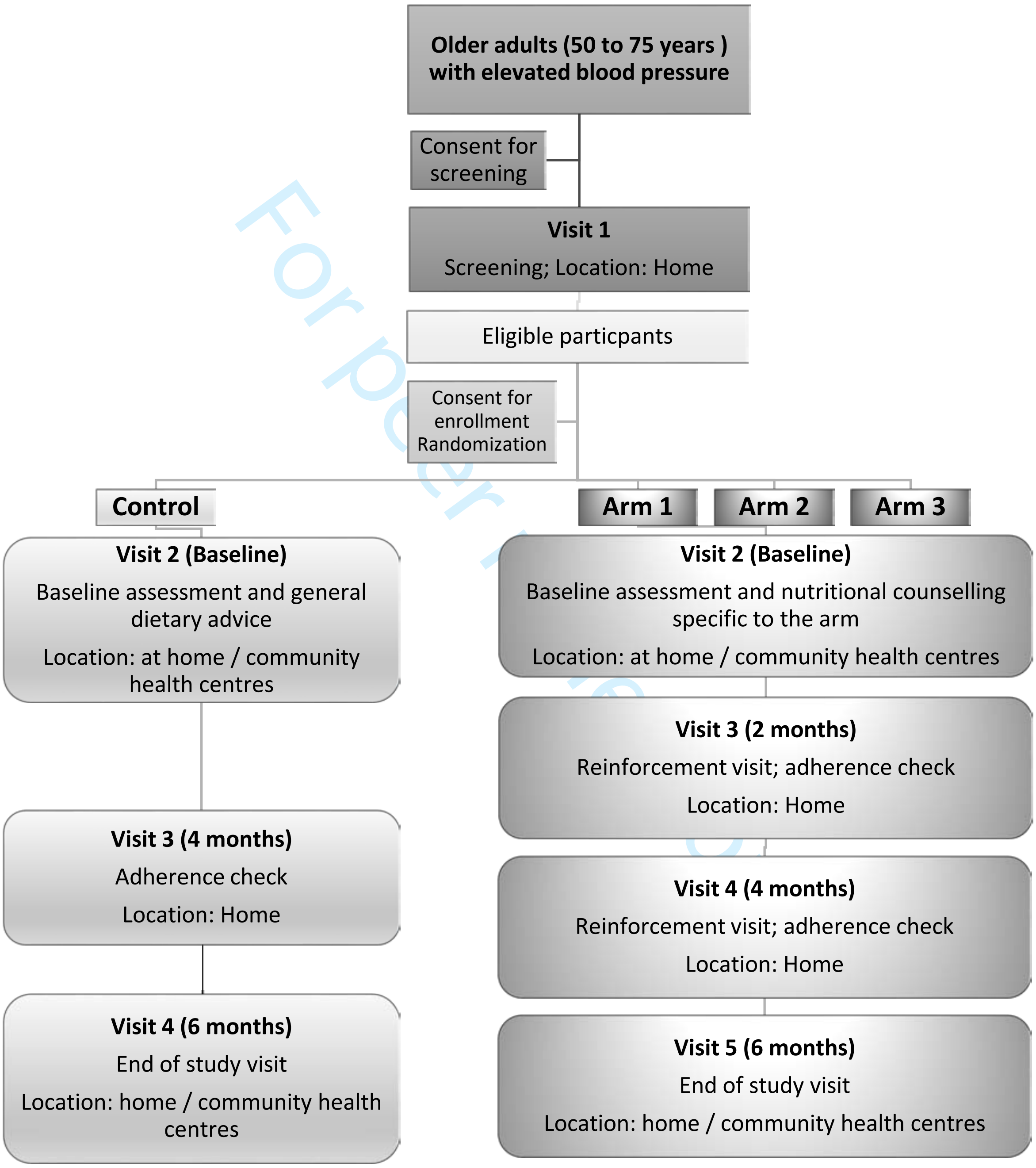

Figure 1: A flow diagram providing an overview of the study procedures

Reporting checklist for protocol of a clinical trial.

Based on the SPIRIT guidelines.

Instructions to authors

Complete this checklist by entering the page numbers from your manuscript where readers will find each of the items listed below.

Your article may not currently address all the items on the checklist. Please modify your text to include the missing information. If you are certain that an item does not apply, please write "n/a" and provide a short explanation.

Upload your completed checklist as an extra file when you submit to a journal.

In your methods section, say that you used the SPIRITreporting guidelines, and cite them as:

Chan A-W, Tetzlaff JM, Altman DG, Laupacis A, Gøtzsche PC, Krleža-Jerić K, Hróbjartsson A, Mann H, Dickersin K, Berlin J, Doré C, Parulekar W, Summerskill W, Groves T, Schulz K, Sox H, Rockhold FW, Rennie D, Moher D. SPIRIT 2013 Statement: Defining standard protocol items for clinical trials. Ann Intern Med. 2013;158(3):200-207

		Reporting Item	Page Number
**Administrative information**			
Title	#1	Descriptive title identifying the study design, population, interventions, and, if applicable, trial acronym	1

For peer review only

Trial registration	#2a	Trial identifier and registry name. If not yet registered, name of intended registry	1
Trial registration: data set	#2b	All items from the World Health Organization Trial Registration Data Set	
Protocol version	#3	Date and version identifier	DePEC protocol v1.0 22/10/2019
Funding	#4	Sources and types of financial, material, and other support	19
Roles and responsibilities: contributorship	#5a	Names, affiliations, and roles of protocol contributors	1 & 19
Roles and responsibilities: sponsor contact information	#5b	Name and contact information for the trial sponsor	19
Roles and responsibilities: sponsor and funder	#5c	Role of study sponsor and funders, if any, in study design; collection, management, analysis, and interpretation of data; writing of the report; and the decision to submit the report for publication, including whether they will have ultimate authority over any of these activities	19

For peer review only

Roles and responsibilities: committees	#5d	Composition, roles, and responsibilities of the coordinating centre, steering committee, endpoint adjudication committee, data management team, and other individuals or groups overseeing the trial, if applicable (see Item 21a for data monitoring committee)	n/a
**Introduction**			
Background and rationale	#6a	Description of research question and justification for undertaking the trial, including summary of relevant studies (published and unpublished) examining benefits and harms for each intervention	4-7
Background and rationale: choice of comparators	#6b	Explanation for choice of comparators	4-7
Objectives	#7	Specific objectives or hypotheses	7
Trial design	#8	Description of trial design including type of trial (eg, parallel group, crossover, factorial, single group), allocation ratio, and framework (eg, superiority, equivalence, non-inferiority, exploratory)	11
**Methods: Participants, interventions, and outcomes**			

For peer review only

Study setting	#9	Description of study settings (eg, community clinic, academic hospital) and list of countries where data will be collected. Reference to where list of study sites can be obtained	8
Eligibility criteria	#10	Inclusion and exclusion criteria for participants. If applicable, eligibility criteria for study centres and individuals who will perform the interventions (eg, surgeons, psychotherapists)	8-9
Interventions: description	#11a	Interventions for each group with sufficient detail to allow replication, including how and when they will be administered	11
Interventions: modifications	#11b	Criteria for discontinuing or modifying allocated interventions for a given trial participant (eg, drug dose change in response to harms, participant request, or improving / worsening disease)	n/a
Interventions: adherance	#11c	Strategies to improve adherence to intervention protocols, and any procedures for monitoring adherence (eg, drug tablet return; laboratory tests)	n/a
Interventions: concomitant care	#11d	Relevant concomitant care and interventions that are permitted or prohibited during the trial	n/a
Outcomes	#12	Primary, secondary, and other outcomes, including the specific measurement variable (eg, systolic blood pressure), analysis metric (eg, change from baseline,	11-16

For peer review only

		final value, time to event), method of aggregation (eg, median, proportion), and time point for each outcome. Explanation of the clinical relevance of chosen efficacy and harm outcomes is strongly recommended	
Participant timeline	#13	Time schedule of enrolment, interventions (including any run-ins and washouts), assessments, and visits for participants. A schematic diagram is highly recommended (see Figure)	10 & 12
Sample size	#14	Estimated number of participants needed to achieve study objectives and how it was determined, including clinical and statistical assumptions supporting any sample size calculations	16
Recruitment	#15	Strategies for achieving adequate participant enrolment to reach target sample size	8
**Methods: Assignment of interventions (for controlled trials)**			
Allocation: sequence generation	#16a	Method of generating the allocation sequence (eg, computer-generated random numbers), and list of any factors for stratification. To reduce predictability of a random sequence, details of any planned restriction (eg, blocking) should be provided in a	11

For peer review only

		separate document that is unavailable to those who enrol participants or assign interventions	
Allocation concealment mechanism	#16b	Mechanism of implementing the allocation sequence (eg, central telephone; sequentially numbered, opaque, sealed envelopes), describing any steps to conceal the sequence until interventions are assigned	11
Allocation: implementation	#16c	Who will generate the allocation sequence, who will enrol participants, and who will assign participants to interventions	11
Blinding (masking)	#17a	Who will be blinded after assignment to interventions (eg, trial participants, care providers, outcome assessors, data analysts), and how	11
Blinding (masking): emergency unblinding	#17b	If blinded, circumstances under which unblinding is permissible, and procedure for revealing a participant's allocated intervention during the trial	11
**Methods: Data collection, management, and analysis**			
Data collection plan	#18a	Plans for assessment and collection of outcome, baseline, and other trial data, including any related processes to promote data quality (eg, duplicate measurements, training of assessors) and a	17

For peer review only

		description of study instruments (eg, questionnaires, laboratory tests) along with their reliability and validity, if known. Reference to where data collection forms can be found, if not in the protocol	
Data collection plan: retention	#18b	Plans to promote participant retention and complete follow-up, including list of any outcome data to be collected for participants who discontinue or deviate from intervention protocols	17
Data management	#19	Plans for data entry, coding, security, and storage, including any related processes to promote data quality (eg, double data entry; range checks for data values). Reference to where details of data management procedures can be found, if not in the protocol	17
Statistics: outcomes	#20a	Statistical methods for analysing primary and secondary outcomes. Reference to where other details of the statistical analysis plan can be found, if not in the protocol	17
Statistics: additional analyses	#20b	Methods for any additional analyses (eg, subgroup and adjusted analyses)	17
Statistics: analysis population and missing data	#20c	Definition of analysis population relating to protocol non-adherence (eg, as randomised analysis), and any statistical methods to handle missing data (eg, multiple imputation)	17

For peer review only

Methods: Monitoring

Data monitoring: formal committee	#21a	Composition of data monitoring committee (DMC); summary of its role and reporting structure; statement of whether it is independent from the sponsor and competing interests; and reference to where further details about its charter can be found, if not in the protocol. Alternatively, an explanation of why a DMC is not needed	n/a
Data monitoring: interim analysis	#21b	Description of any interim analyses and stopping guidelines, including who will have access to these interim results and make the final decision to terminate the trial	n/a
Harms	#22	Plans for collecting, assessing, reporting, and managing solicited and spontaneously reported adverse events and other unintended effects of trial interventions or trial conduct	17
Auditing	#23	Frequency and procedures for auditing trial conduct, if any, and whether the process will be independent from investigators and the sponsor	17

Ethics and dissemination

Research ethics approval	#24	Plans for seeking research ethics committee / institutional review board (REC / IRB) approval	18

For peer review only

Protocol amendments	#25	Plans for communicating important protocol modifications (eg, changes to eligibility criteria, outcomes, analyses) to relevant parties (eg, investigators, REC / IRBs, trial participants, trial registries, journals, regulators)	n/a
Consent or assent	#26a	Who will obtain informed consent or assent from potential trial participants or authorised surrogates, and how (see Item 32)	8
Consent or assent: ancillary studies	#26b	Additional consent provisions for collection and use of participant data and biological specimens in ancillary studies, if applicable	n/a
Confidentiality	#27	How personal information about potential and enrolled participants will be collected, shared, and maintained in order to protect confidentiality before, during, and after the trial	18
Declaration of interests	#28	Financial and other competing interests for principal investigators for the overall trial and each study site	1
Data access	#29	Statement of who will have access to the final trial dataset, and disclosure of contractual agreements that limit such access for investigators	17-18
Ancillary and post trial care	#30	Provisions, if any, for ancillary and post-trial care, and for compensation to those who suffer harm from trial participation	17-18

For peer review only

Dissemination policy: trial results	#31a	Plans for investigators and sponsor to communicate trial results to participants, healthcare professionals, the public, and other relevant groups (eg, via publication, reporting in results databases, or other data sharing arrangements), including any publication restrictions	17-18
Dissemination policy: authorship	#31b	Authorship eligibility guidelines and any intended use of professional writers	n/a
Dissemination policy: reproducible research	#31c	Plans, if any, for granting public access to the full protocol, participant-level dataset, and statistical code	n/a
**Appendices**			
Informed consent materials	#32	Model consent form and other related documentation given to participants and authorised surrogates	n/a
Biological specimens	#33	Plans for collection, laboratory evaluation, and storage of biological specimens for genetic or molecular analysis in the current trial and for future use in ancillary studies, if applicable	n/a

None The SPIRIT checklist is distributed under the terms of the Creative Commons Attribution License CC-BY-ND 3.0. This checklist can be completed online using https://www.goodreports.org/, a tool made by the EQUATOR Network in collaboration with Penelope.ai

For peer review only
